# Supplementary material for: PKN2 deficiency leads both to prenatal ‘congenital’ cardiomyopathy and defective angiotensin II stress responses
Source: Biochem J. 2022 Jul 12;479(13):1467–86. doi: 10.1042/BCJ20220281 (PMC9342899; doi:10.1042/BCJ20220281)
Supplement: Supplementary Material 1 [file BCJ-479-1467-s1.pdf]

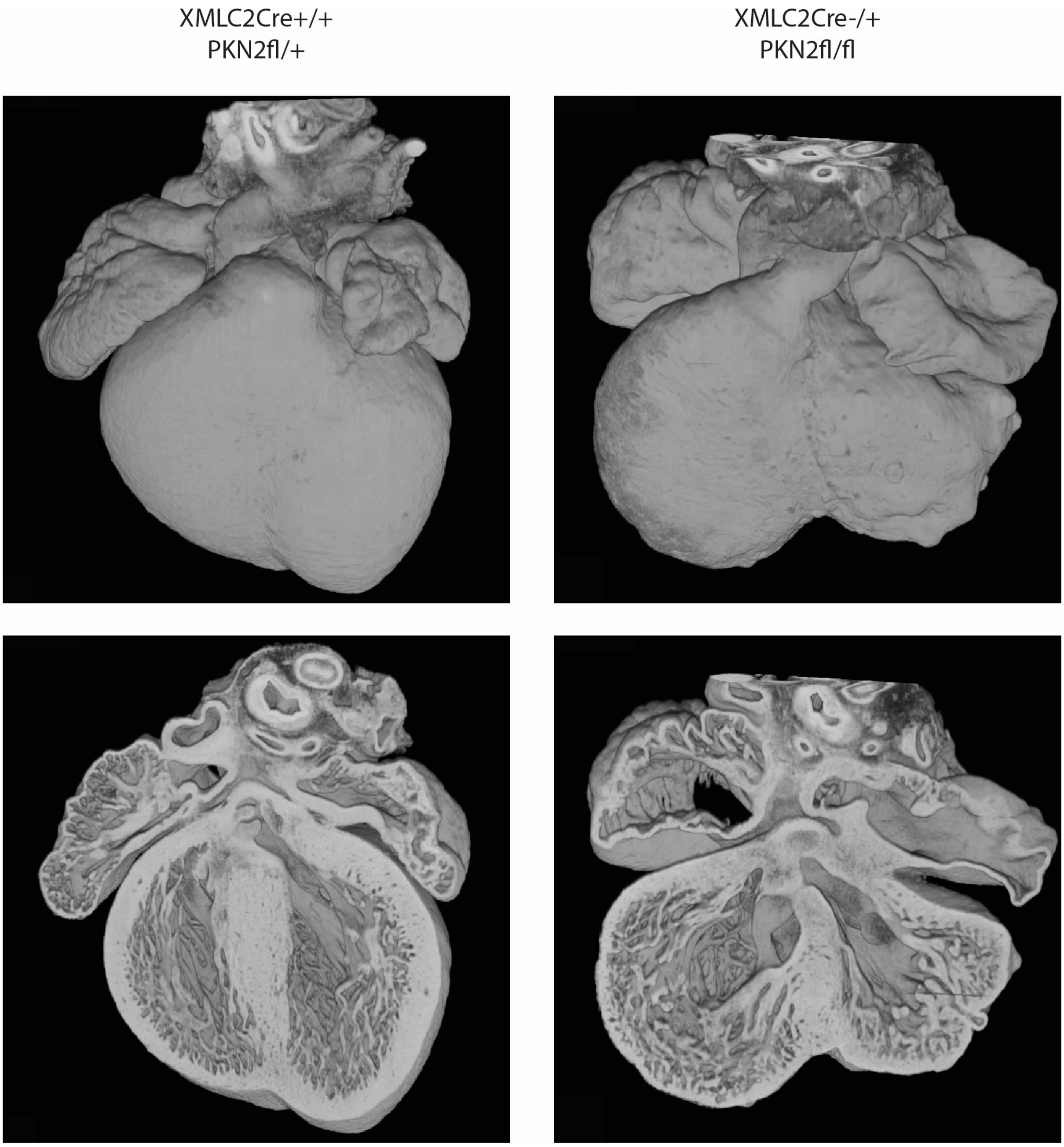

Figure S1. HREM images of XMLC2 knockout of Pkn2

HREM images are from a reconstruction of a 14.5d XMLC2Cre$^{+/-}$ *Pkn2*$^{fl/+}$ embryo (left) and of a XMLC2Cre$^{+/-}$*Pkn2*$^{fl/fl}$ (right) from the same dam. The upper panels are surface images and the lower panels illustrate sections.

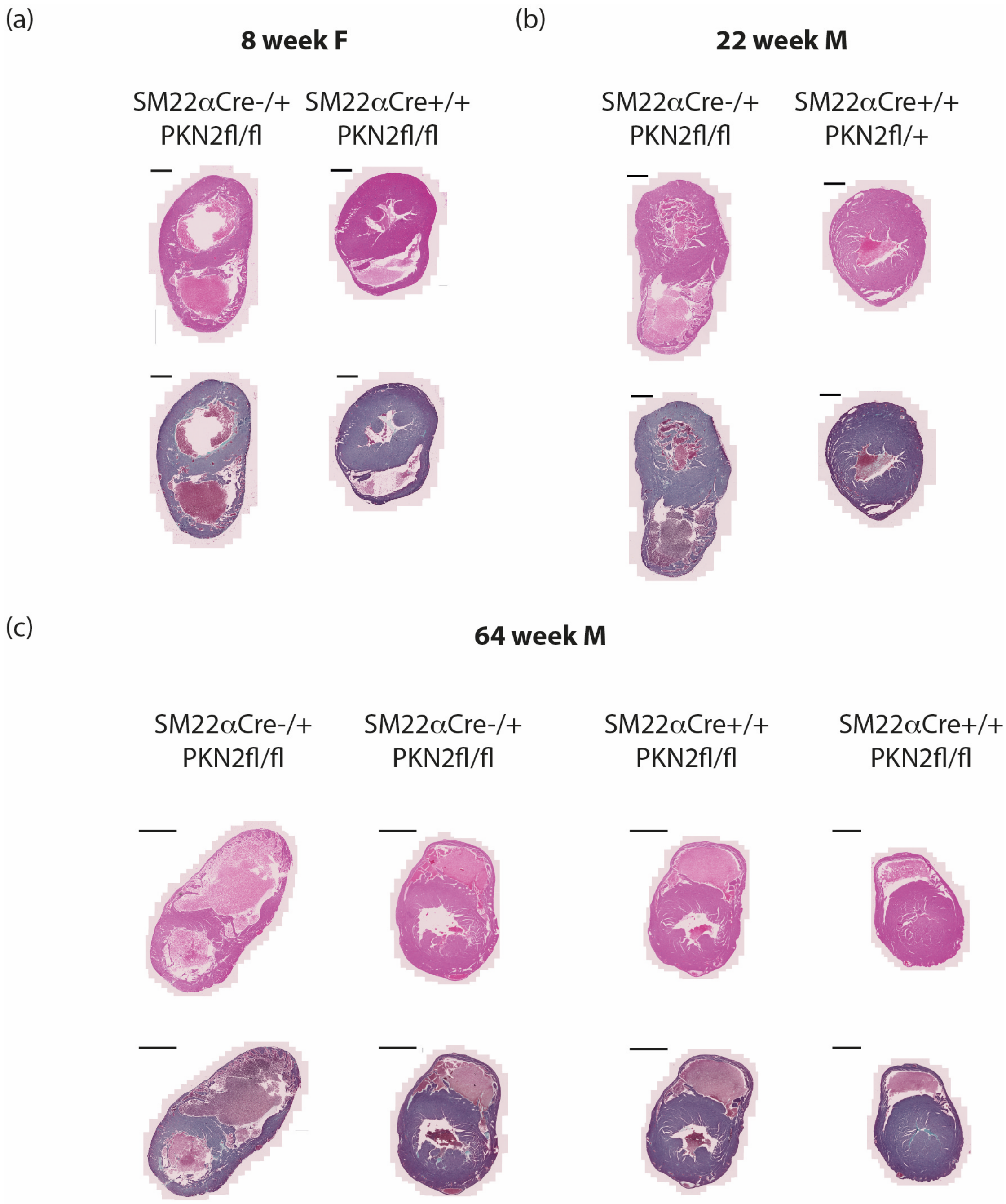

Figure S2. Histology of sections from the SM22α knockout of Pkn2 in adult hearts

H&E (top rows) and Gomori's Trichrome (lower rows) stained sections through the short-axis of the heart, at 2-3 mm from the apex from littermates of (a) females culled at 8 weeks of age, (b) males at 22 weeks, and (c) males at 64 weeks, with genotypes as labelled. For each group, cull was triggered due to loss of condition of one littermate (left in each group). Scale bars are (a,b) 1 mm or (c) 2 mm.

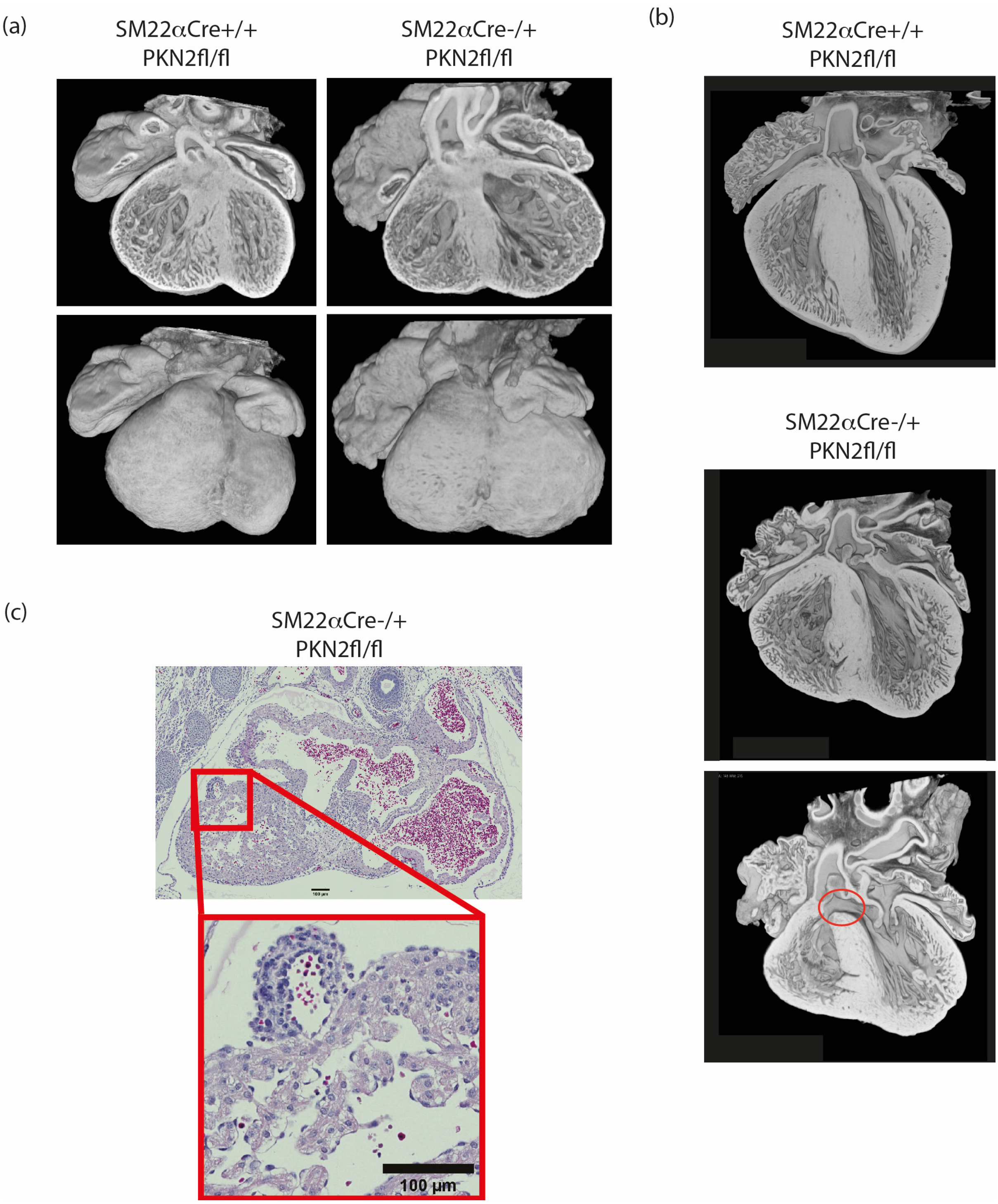

Figure S3 Embryonic defects SM22α Pkn2 mice

HREM images are from reconstructions of embyros collected at (a) E14.5 and (b) E18.5 days gestation. Genotypes are as indicated. (c) H&E stained section of a SM22αCre$^{+/-}$ *Pkn2*$^{fl/fl}$ E14.5 heart with 100 µm scale, as labelled.

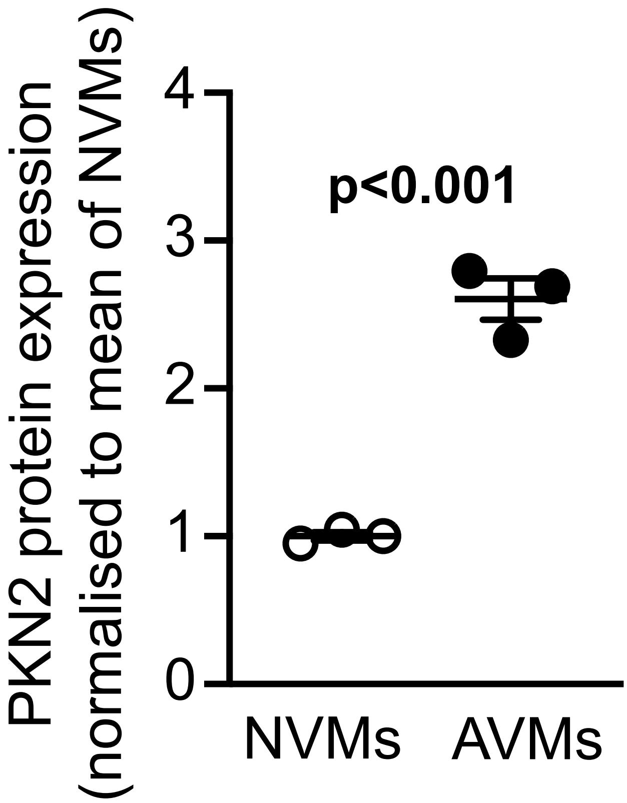

Figure S4. Expression of PKN2 in neonatal rat ventricular myocytes (NVMs) compared with adult rat ventricular myocytes (AVMs) relative to cell size. Expression data were from Fuller SJ et al. (Cardiovasc Res. 2015; 108: 87-98) adjusted for cell size according to membrane capacitance which increases from 13 pF in 1- to 2-day NVMs to 156 pF in AVMs [60, 61].

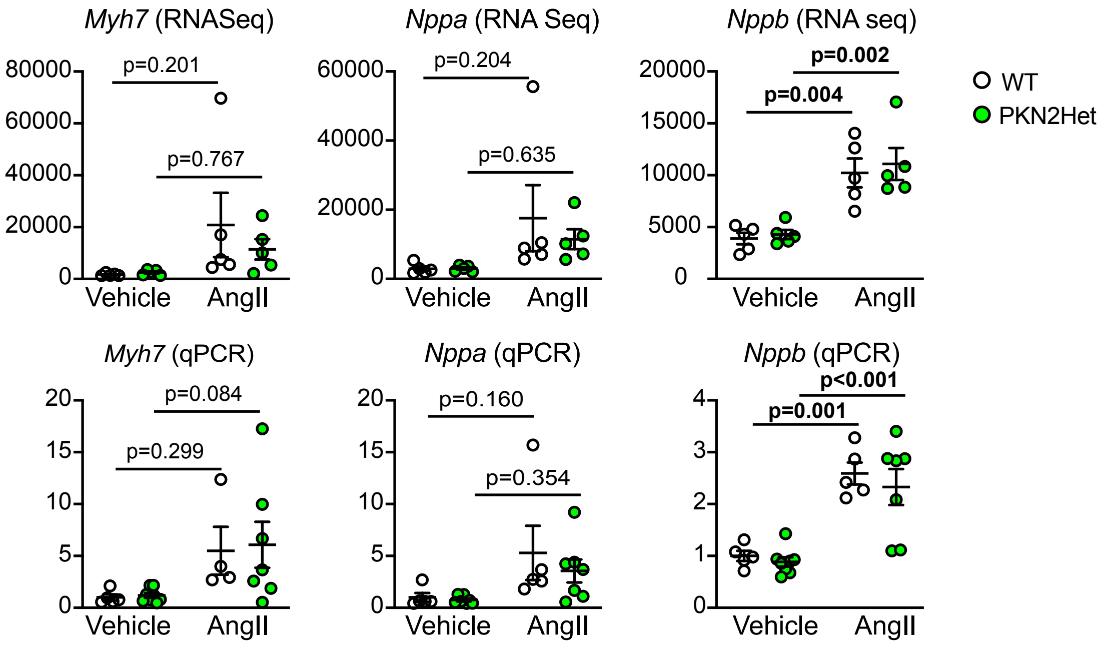

Figure S5. Expression of hypertrophy associated genes in hearts from WT or Pkn2Het mice. WT (white) or Pkn2Het (green) mice were treated with vehicle (left side of each graph) or AngII (0.8 mg/kg/d; right side of each graph) for 7 d. RNA was prepared and used for RNASeq (upper panels) or qPCR (lower panels). Individual data points are shown with means ± SEM. Analysis used 2-way ANOVA with Holm-Sidak's post-test.

Supplementary Table S1. Echocardiography data for 12 and 42 week male mice with heterozygous PKN2 gene deletion (PKN2Het) and wild-type (WT) mice: baseline data. AAT, aortic acceleration time; AET, Aortic expulsion time; VTI, velocity time interval; PAT, pulmonary acceleration time; PET, pulmonary expulsion time; PA, pulmonary artery; LV, left ventricle; ID, internal diameter; AW, anterior wall; PW, posterior wall.

	**WT: 12 wk (n=10)**		**PKN2Het: 12 wk (n=15)**		**WT: 42 wk (n=8)**		**PKN2Het: 42 wk (n=10)**	
	Mean	SEM	Mean	SEM	Mean	SEM	Mean	SEM
**Aortic flow (Pulsed Wave Doppler)**								
AAT (ms)	23.80	0.82	23.59	0.72	18.98	0.72	20.03	1.08
AET (ms)	57.21	0.68	56.55	0.68	48.54	1.13	53.56	1.77
AAT/AET	0.42	0.01	0.42	0.01	0.39	0.01	0.37	0.01
Aorta VTI (mm)	61.95	2.47	60.71	2.86	40.31	3.09	55.47	3.77
Aorta Mean Velocity (mm/s)	821.32	29.33	800.96	29.24	620.71	36.58	800.88	54.81
Aorta Mean Gradient (mmHg)	2.81	0.23	2.65	0.19	1.59	0.18	2.69	0.33
Aorta Peak Velocity (mm/s	1632.92	58.53	1592.77	53.01	1271.29	82.00	1620.63	104.77
Aorta Peak Grad (mmHg)	11.08	0.93	10.46	0.65	6.69	0.83	10.94	1.32
Aorta Peak Pressure (mmHg)	10.85	0.86	10.31	0.65	6.64	0.82	10.81	1.31
**Pulmonary flow (Pulsed Wave Doppler)**								
PAT (ms)	23.71	0.53	24.90	0.37	24.97	0.98	24.71	0.72
PET (ms)	60.47	0.92	61.43	0.65	56.74	2.15	58.94	2.42
PAT/PET	0.39	0.01	0.41	0.00	0.44	0.02	0.42	0.01
PA VTI	32.11	1.12	32.70	0.58	20.66	1.20	21.44	0.97
PA Mean Velocity (mm/s)	-369.58	10.38	-360.73	12.93	-262.10	8.32	-264.84	9.79
PA Mean Gradient (mmHg)	0.56	0.03	0.56	0.02	0.28	0.02	0.29	0.02
PA Peak Velocity (mm/s	-764.05	17.63	-740.28	23.98	-551.01	13.94	-558.99	25.85
PA Peak Grad (mmHg)	2.37	0.11	2.36	0.08	1.22	0.06	1.28	0.12
**Aorta diameter (B-Mode)**								
Widest (mm)	1.51	0.01	1.54	0.02	1.59	0.03	1.60	0.03
Narrowest (mm	1.28	0.02	1.29	0.02	1.47	0.04	1.44	0.04
Wide/narrow	1.20	0.01	1.19	0.01	1.08	0.01	1.11	0.02
**Left ventricle dimensions (short axis M-mode)**								
Heart Rate (bpm	522.13	11.06	508.26	11.26	473.62	17.63	448.95	18.55
LVID;s (mm)	2.97	0.05	2.92	0.05	2.59	0.07	2.71	0.05
LVID;d (mm)	4.18	0.05	4.14	0.05	3.80	0.06	3.95	0.04
LVAW;s (mm)	1.11	0.02	1.05	0.01	1.10	0.04	1.07	0.03
LVAW;d (mm)	0.84	0.02	0.77	0.01	0.84	0.02	0.81	0.02
LVPW;s (mm)	1.03	0.03	1.01	0.02	1.16	0.03	1.11	0.05
LVPW;d (mm)	0.72	0.02	0.69	0.01	0.78	0.04	0.77	0.02
**Cardiac function (speckle-tracking strain analysis; long axis B-mode)**								
Heart rate (bpm	502.23	10.29	487.39	10.63	459.97	22.21	460.58	22.49
Stroke volume (µl)	25.51	1.35	26.23	0.76	24.18	2.71	24.17	2.51
Fractional shortening (%)	26.85	0.76	28.45	0.85	28.04	1.43	29.33	2.73
Ejection fraction (%)	50.11	1.93	53.31	1.20	53.38	2.76	54.07	2.95
Cardiac output (ml/min)	12.76	0.59	12.79	0.49	10.94	1.10	11.01	1.04
End diastolic LV mass	53.48	1.45	49.95	0.88	64.83	4.30	66.52	2.59
End systolic LV mass	56.46	1.57	52.34	0.75	66.08	4.28	67.84	2.70

Supplementary Table S2. Echocardiography data for 12 week male mice with heterozygous PKN2 gene deletion (PKN2Het) and wild-type (WT) mice treated for 7 d with 0.8 mg/kg/d angiotensin 2 or vehicle. AAT, aortic acceleration time; AET, Aortic expulsion time; VTI, velocity time interval; PAT, pulmonary acceleration time; PET, pulmonary expulsion time; PA, pulmonary artery; LV, left ventricle; ID, internal diameter; AW, anterior wall; PW, posterior wall.

	**WT: vehicle (n=5)**		**PKN2Het: vehicle (n=8)**		**WT: AngII (n=5)**		**PKN2Het: AngII (n=7)**	
	Mean	SEM	Mean	SEM	Mean	SEM	Mean	SEM
**Aortic flow (Pulsed Wave Doppler)**								
AAT (ms)	23.50	1.19	22.76	0.86	24.42	1.23	22.53	1.44
AET (ms)	57.71	1.57	55.21	1.39	56.29	1.65	54.49	1.23
AAT/AET	0.41	0.03	0.41	0.01	0.43	0.02	0.41	0.02
Aorta VTI (mm)	61.67	5.44	58.84	4.05	58.82	3.16	61.60	5.13
Aorta Mean Velocity (mm/s)	780.00	63.10	819.21	59.70	811.40	45.61	864.12	57.41
Aorta Mean Gradient (mmHg)	2.53	0.41	2.80	0.37	2.68	0.30	3.08	0.42
Aorta Peak Velocity (mm/s	1583.20	128.93	1607.53	109.14	1498.86	29.58	1689.25	81.15
Aorta Peak Grad (mmHg)	10.32	1.68	10.71	1.32	9.01	0.35	11.60	1.08
Aorta Peak Pressure (mmHg)	10.20	1.68	10.61	1.30	8.91	0.35	11.50	1.09
**Pulmonary flow (Pulsed Wave Doppler)**								
PAT (ms)	26.13	1.02	25.44	1.09	24.54	0.58	22.71	1.25
PET (ms)	62.38	1.29	63.13	1.47	58.68	2.93	56.67	1.72
PAT/PET	0.42	0.01	0.40	0.01	0.42	0.02	0.40	0.02
PA VTI	31.66	1.74	31.73	1.05	28.44	2.24	25.39	1.39
PA Mean Velocity (mm/s)	-362.61	16.48	-363.12	13.26	-333.86	18.50	-310.38	10.82
PA Mean Gradient (mmHg)	0.53	0.05	0.53	0.04	0.45	0.05	0.39	0.03
PA Peak Velocity (mm/s	-755.55	28.94	-744.09	26.15	-704.73	33.94	-682.22	25.60
PA Peak Grad (mmHg)	2.30	0.18	2.24	0.16	2.01	0.20	1.88	0.14
**Aorta diameter (B-Mode)**								
Widest (mm)	1.51	0.02	1.51	0.01	1.63	0.06	1.69	0.08
Narrowest (mm	1.21	0.02	1.27	0.03	1.44	0.09	1.56	0.09
Wide/narrow	1.24	0.02	1.19	0.03	1.14	0.04	1.09	0.01
**Left ventricle dimensions (short axis M-mode)**								
Heart Rate (bpm	501.28	13.71	506.12	12.71	528.57	15.67	521.25	17.10
LVID;s (mm)	2.96	0.11	3.01	0.07	2.59	0.08	2.68	0.11
LVID;d (mm)	4.17	0.10	4.15	0.05	3.86	0.13	3.80	0.14
LVAW;s (mm)	1.08	0.04	1.07	0.01	1.29	0.06	1.15	0.02
LVAW;d (mm)	0.82	0.03	0.81	0.02	1.00	0.05	0.91	0.02
LVPW;s (mm)	1.03	0.06	1.02	0.03	1.31	0.07	1.18	0.02
LVPW;d (mm)	0.72	0.05	0.72	0.03	1.02	0.09	0.85	0.04
**Cardiac function (speckle-tracking strain analysis; long axis B-mode)**								
Heart rate (bpm	462.84	9.65	503.48	16.63	525.26	14.72	512.86	19.44
Stroke volume (µl)	28.52	2.63	28.07	1.03	27.49	3.19	21.12	2.08
Fractional shortening (%)	24.54	1.58	27.42	1.62	33.15	2.06	25.56	1.69
Ejection fraction (%)	49.85	1.59	53.86	1.99	60.29	3.22	52.70	1.28
Cardiac output (ml/min)	13.21	1.31	14.22	0.90	14.41	1.70	10.68	0.90
End diastolic LV mass	54.15	4.23	51.75	2.14	71.11	5.80	54.44	4.21
End systolic LV mass	57.33	5.02	56.87	2.64	76.33	5.51	57.72	4.56

Supplementary Table S3. RNASeq analysis of effects of angiotensin II (AngII) on mRNA expression in hearts from PKN2Het vs WT littermates: mRNAs significantly upregulated by AngII in PKN2Het or WT hearts.

Gene Symbol	Ensembl gene id	WT Vehicle		WT AngII		PKN2Het Vehicle		PKN2Het AngII	
		Mean	SD	Mean	SD	Mean	SD	Mean	SD
Sept5	ENSMUSG00000072214	83	20	175	64	94	15	166	50
Sept9	ENSMUSG00000059248	865	99	1148	72	882	89	1148	182
Sept11	ENSMUSG00000058013	781	129	1170	161	705	99	998	196
1500009L16Rik	ENSMUSG00000087651	50	8	89	32	43	11	85	10
1700120C14Rik	ENSMUSG00000100599	84	4	153	41	76	23	122	23
2010111I01Rik	ENSMUSG00000021458	946	73	1185	106	902	74	1185	115
Abcg1	ENSMUSG00000024030	121	26	192	24	107	25	163	40
Acan	ENSMUSG00000030607	4	3	66	49	3	1	32	34
Ace	ENSMUSG00000020681	1467	152	2473	345	1594	190	2408	305
Acta1	ENSMUSG00000031972	6773	1395	24772	10833	8570	3863	28601	14894
Actn1	ENSMUSG00000015143	522	79	925	273	524	61	761	132
Actr3	ENSMUSG00000026341	2536	120	3001	264	2399	43	2908	199
Adam12	ENSMUSG00000054555	55	9	167	53	41	13	115	49
Adam15	ENSMUSG00000028041	1010	154	1434	80	997	96	1364	151
Adamts12	ENSMUSG00000047497	91	12	261	100	100	8	204	80
Adamts2	ENSMUSG00000036545	499	49	1369	604	526	39	1080	305
Adamts8	ENSMUSG00000031994	16	4	76	21	15	2	57	28
Adamtsl2	ENSMUSG00000036040	230	49	554	141	251	43	511	150
Adcy7	ENSMUSG00000031659	355	49	714	204	342	76	589	111
Adgre1	ENSMUSG00000004730	375	73	763	304	349	35	608	121
AI506816	ENSMUSG00000105987	463	36	655	41	454	52	676	86
Aif1	ENSMUSG00000024397	63	12	133	73	51	11	95	13
Aldh1a2	ENSMUSG00000013584	143	34	307	88	145	27	322	123
Ankrd1	ENSMUSG00000024803	20150	6483	57246	17002	19310	6244	49450	10874
Ankrd23	ENSMUSG00000067653	8223	1670	13521	3447	9104	579	14731	4085
Anln	ENSMUSG00000036777	56	22	225	55	56	7	174	50
Anxa1	ENSMUSG00000024659	596	70	1111	254	555	84	919	152
Anxa2	ENSMUSG00000032231	1328	74	2290	204	1236	149	1957	280
Anxa5	ENSMUSG00000027712	1991	157	2628	137	1995	184	2495	432
Apbb1ip	ENSMUSG00000026786	129	14	240	62	146	18	205	16
Apod	ENSMUSG00000022548	115	26	229	22	122	16	198	30
Apoe	ENSMUSG00000002985	3788	666	6913	2610	4103	455	5864	858
Arhgap1	ENSMUSG00000027247	496	38	622	41	520	32	610	36
Arhgap11a	ENSMUSG00000041219	76	15	183	32	69	9	158	44
Arhgap30	ENSMUSG00000048865	154	24	265	55	147	24	206	37
Arhgdib	ENSMUSG00000030220	506	27	664	56	483	58	627	60
Arhgef40	ENSMUSG00000004562	530	50	747	111	504	59	674	68
Arid5a	ENSMUSG00000037447	177	60	315	53	146	28	315	68
Arl4c	ENSMUSG00000049866	257	44	447	45	299	36	401	52
Arl6ip1	ENSMUSG00000030654	555	29	783	95	531	41	675	58
Arpc1b	ENSMUSG00000029622	814	81	1186	179	737	113	1071	150
Arpc3	ENSMUSG00000029465	1134	39	1490	168	1115	115	1392	88
Arpc5	ENSMUSG00000008475	864	45	1233	156	871	66	1121	40
Asap2	ENSMUSG00000052632	656	118	829	131	621	58	818	121
Aspm	ENSMUSG00000033952	46	20	147	29	43	10	129	39
Atad2	ENSMUSG00000022360	124	14	243	23	132	24	212	55
Atf4	ENSMUSG00000042406	1171	85	1374	69	1169	114	1319	150
Atp10a	ENSMUSG00000025324	58	4	114	20	63	11	98	18
Atp8a2	ENSMUSG00000021983	247	26	365	72	234	36	355	74
Atp8b1	ENSMUSG00000039529	239	30	380	49	213	28	351	75
Aurka	ENSMUSG00000027496	18	5	51	16	19	6	46	15
Aurkb	ENSMUSG00000020897	18	6	47	18	16	5	39	13
Axl	ENSMUSG00000002602	1982	499	2556	293	1813	317	2326	298
B3galnt1	ENSMUSG00000043300	50	8	82	21	50	8	80	7
Basp1	ENSMUSG00000045763	41	7	103	33	42	3	76	15
BC028528	ENSMUSG00000038543	144	14	227	38	130	24	206	24
Bgn	ENSMUSG00000031375	5175	520	12514	5386	5152	464	9491	2127
Birc5	ENSMUSG00000017716	20	10	66	13	18	6	66	24
Bmp1	ENSMUSG00000022098	505	77	897	297	496	46	796	219

Bub1	ENSMUSG00000027379	12	2	48	14	16	9	44	19
Bub1b	ENSMUSG00000040084	41	19	125	41	51	17	103	38
C1qa	ENSMUSG00000036887	895	96	1523	250	883	97	1362	183
C1qb	ENSMUSG00000036905	826	111	1513	429	782	128	1330	245
C1qc	ENSMUSG00000036896	886	106	1454	199	845	131	1290	171
C1qtnf6	ENSMUSG00000022440	122	21	426	250	120	11	348	107
C4b	ENSMUSG00000073418	231	28	604	141	295	41	861	703
Cald1	ENSMUSG00000029761	1649	323	2616	191	1624	256	2205	422
Cap1	ENSMUSG00000028656	1199	155	1633	55	1115	167	1503	127
Capg	ENSMUSG00000056737	183	29	364	36	182	36	328	70
Capza1	ENSMUSG00000070372	1251	78	1608	138	1166	148	1428	124
Carhsp1	ENSMUSG00000008393	592	69	889	70	517	68	796	96
Casp4	ENSMUSG00000033538	79	11	119	19	74	18	113	27
Casp8	ENSMUSG00000026029	191	12	303	44	192	23	274	37
Cavin3	ENSMUSG00000037060	300	26	506	43	295	23	430	88
Ccdc80	ENSMUSG00000022665	1816	295	3479	1261	1843	168	2847	551
Ccl8	ENSMUSG00000009185	11	7	75	31	17	9	73	59
Ccna2	ENSMUSG00000027715	65	39	245	89	48	9	184	81
Ccnb1	ENSMUSG00000041431	24	11	73	20	14	4	71	30
Ccnb2	ENSMUSG00000032218	27	10	82	39	18	5	72	21
Cd109	ENSMUSG00000046186	101	22	257	86	92	18	181	52
Cd14	ENSMUSG00000051439	89	13	165	34	94	6	143	26
Cd248	ENSMUSG00000056481	285	55	497	49	278	22	431	86
Cd300c2	ENSMUSG00000044811	33	5	109	72	32	8	66	20
Cd300ld	ENSMUSG00000034641	151	30	245	51	133	23	238	49
Cd34	ENSMUSG00000016494	3352	231	5107	337	3106	396	4820	637
Cd44	ENSMUSG00000005087	292	53	505	44	273	42	409	82
Cd48	ENSMUSG00000015355	78	10	145	48	67	12	120	19
Cd68	ENSMUSG00000018774	205	25	321	48	181	27	308	61
Cd72	ENSMUSG00000028459	30	9	159	128	27	7	92	56
Cd84	ENSMUSG00000038147	88	19	178	51	80	9	164	32
Cd9	ENSMUSG00000030342	590	47	756	24	581	71	670	65
Cd93	ENSMUSG00000027435	3708	657	5435	821	3856	571	5157	633
Cdc20	ENSMUSG00000006398	28	11	91	19	23	7	80	21
Cdca3	ENSMUSG00000023505	16	5	56	20	12	5	59	28
Cdca5	ENSMUSG00000024791	5	2	21	8	5	3	21	10
Cdca8	ENSMUSG00000028873	14	3	49	5	13	6	38	18
Cdk1	ENSMUSG00000019942	33	11	194	49	35	17	130	47
Cdkn1a	ENSMUSG00000023067	351	153	609	89	367	175	544	152
Cenpe	ENSMUSG00000045328	62	41	172	56	38	21	116	38
Cep55	ENSMUSG00000024989	17	5	63	15	12	2	58	27
Cfb	ENSMUSG00000090231	36	7	140	61	33	15	114	78
Cfl1	ENSMUSG00000056201	2052	87	2670	304	1872	236	2490	204
Ch25h	ENSMUSG00000050370	11	6	32	14	13	5	34	17
Chd9	ENSMUSG00000056608	1089	68	1541	297	1030	53	1413	164
Cilp	ENSMUSG00000042254	294	76	3092	3273	401	134	2249	1515
Ckap2	ENSMUSG00000037725	33	16	131	46	25	5	95	45
Ckap2l	ENSMUSG00000048327	30	10	123	39	25	8	110	29
Ckap4	ENSMUSG00000046841	756	159	1094	75	815	103	1104	165
Cks2	ENSMUSG00000062248	13	1	50	13	9	2	35	11
Clec4d	ENSMUSG00000030144	9	3	26	14	4	2	18	9
Clec4n	ENSMUSG00000023349	32	6	110	48	34	9	86	29
Clec5a	ENSMUSG00000029915	57	11	99	10	54	23	89	10
Clic1	ENSMUSG00000007041	641	18	1053	159	606	66	943	94
Cmtm3	ENSMUSG00000031875	323	45	510	56	301	38	439	77
Cnn3	ENSMUSG00000053931	1009	125	1438	123	955	121	1319	119
Cnot6	ENSMUSG00000020362	832	68	999	41	747	64	909	122
Col12a1	ENSMUSG00000032332	47	12	495	623	42	10	315	288
Col14a1	ENSMUSG00000022371	466	75	1493	1179	377	75	1075	556
Col15a1	ENSMUSG00000028339	2833	383	5668	1135	3175	509	5335	1392
Col18a1	ENSMUSG00000001435	248	28	790	244	293	35	541	181
Col3a1	ENSMUSG00000026043	5494	744	25906	20347	5871	824	16747	9657
Col4a1	ENSMUSG00000031502	12856	2211	23909	1751	13123	855	20837	3837
Col4a2	ENSMUSG00000031503	9490	1105	15270	591	9814	526	14603	2361
Col4a4	ENSMUSG00000067158	345	73	497	114	364	54	494	37
Col5a1	ENSMUSG00000026837	1205	173	3410	1633	1238	85	2682	1054
Col5a2	ENSMUSG00000026042	878	65	4055	3237	927	105	2738	1563

Col6a1	ENSMUSG00000001119	2071	252	3943	1376	2047	155	3242	704
Col6a2	ENSMUSG00000020241	2038	291	3824	981	2062	139	3256	743
Col6a3	ENSMUSG00000048126	1170	165	2685	1137	1119	203	1895	452
Col8a1	ENSMUSG00000068196	704	96	3461	2170	747	153	2473	1081
Cotl1	ENSMUSG00000031827	234	40	369	66	208	50	335	22
Creb5	ENSMUSG00000053007	235	36	347	57	199	28	323	65
Crip1	ENSMUSG00000006360	686	77	907	72	645	76	846	125
Crlf1	ENSMUSG00000007888	11	7	82	61	12	6	50	29
Csf1r	ENSMUSG00000024621	1029	156	1681	243	1120	141	1491	195
Csrp2	ENSMUSG00000020186	180	37	542	354	165	20	315	127
Ctgf	ENSMUSG00000019997	1416	407	4434	1114	1381	216	3773	916
Ctla2a	ENSMUSG00000044258	445	55	611	63	463	136	614	55
Ctsc	ENSMUSG00000030560	1077	100	1506	162	1055	115	1329	214
Ctsz	ENSMUSG00000016256	454	61	730	152	421	29	666	78
Cxcl16	ENSMUSG00000018920	209	30	426	208	210	55	332	47
Dab2	ENSMUSG00000022150	1262	200	1666	115	1122	167	1571	239
Dbn1	ENSMUSG00000034675	170	35	385	71	169	25	330	74
Dchs1	ENSMUSG00000036862	677	75	906	45	647	56	875	104
Depdc1a	ENSMUSG00000028175	7	6	46	33	10	5	34	14
Diaph3	ENSMUSG00000022021	16	5	59	16	23	10	55	26
Dio2	ENSMUSG00000007682	59	18	224	44	57	23	165	64
Dlgap5	ENSMUSG00000037544	17	6	48	15	12	6	41	13
Dpysl3	ENSMUSG00000024501	1022	66	1824	480	1008	79	1535	363
Dtl	ENSMUSG00000037474	13	10	42	13	12	3	42	11
Dynll1	ENSMUSG00000009013	571	82	870	205	474	104	727	130
E2f1	ENSMUSG00000027490	21	5	59	13	23	8	56	20
E2f7	ENSMUSG00000020185	42	13	85	32	34	13	79	29
Ecm1	ENSMUSG00000028108	452	86	696	127	464	82	720	136
Ecscr	ENSMUSG00000073599	254	10	366	51	246	20	337	40
Ect2	ENSMUSG00000027699	21	8	80	27	24	9	75	24
Edem1	ENSMUSG00000030104	377	69	588	97	278	84	510	147
Eef1a1	ENSMUSG00000037742	19836	2225	28730	7994	17884	2426	23968	2075
Efhd2	ENSMUSG00000040659	427	47	656	180	426	31	620	78
Elf4	ENSMUSG00000031103	320	62	496	40	293	54	437	22
Emilin1	ENSMUSG00000029163	473	106	835	204	519	28	812	91
Emp1	ENSMUSG00000030208	1538	159	4210	1587	1504	221	3295	791
Emp3	ENSMUSG00000040212	145	17	216	17	152	17	192	12
Enah	ENSMUSG00000022995	2820	352	4077	860	3138	213	4243	467
Endod1	ENSMUSG00000037419	225	35	387	85	188	20	328	98
Entpd1	ENSMUSG00000048120	473	39	717	165	458	60	637	114
Ereg	ENSMUSG00000029377	2	1	20	9	2	1	13	5
Esco2	ENSMUSG00000022034	12	5	43	10	11	6	36	16
Esyt1	ENSMUSG00000025366	578	37	738	64	571	38	697	35
F2r	ENSMUSG00000048376	819	41	1217	181	861	54	1112	37
F2rl1	ENSMUSG00000021678	27	6	56	22	20	7	44	13
Fads1	ENSMUSG00000010663	476	36	619	11	473	39	586	42
Fam111a	ENSMUSG00000024691	311	25	543	98	278	57	446	66
Fam114a1	ENSMUSG00000029185	316	44	511	131	316	48	398	46
Fam129b	ENSMUSG00000026796	610	83	785	85	572	21	715	78
Fam198b	ENSMUSG00000027955	1065	183	1845	423	1058	202	1912	390
Fbln2	ENSMUSG00000064080	1827	201	3200	493	1966	214	3008	422
Fbn1	ENSMUSG00000027204	2671	543	7690	2514	3011	303	6532	2277
Fcgr2b	ENSMUSG00000026656	275	47	507	137	255	41	437	94
Fcgr3	ENSMUSG00000059498	355	58	659	143	324	56	543	112
Fcgr4	ENSMUSG00000059089	20	9	70	53	17	3	41	16
Fcrls	ENSMUSG00000015852	197	22	463	184	186	29	357	109
Fignl1	ENSMUSG00000035455	26	6	57	22	21	4	51	17
Filip1l	ENSMUSG00000043336	880	82	1259	105	842	147	1084	69
Fkbp10	ENSMUSG00000001555	345	47	461	58	288	35	402	56
Flna	ENSMUSG00000031328	4014	455	5769	673	3783	774	4951	309
Fmnl3	ENSMUSG00000023008	757	91	963	63	730	56	931	86
Fndc1	ENSMUSG00000071984	473	60	1081	567	452	50	911	402
Foxm1	ENSMUSG00000001517	42	12	122	30	47	13	101	53
Frzb	ENSMUSG00000027004	70	18	272	183	82	24	208	55
Fscn1	ENSMUSG00000029581	817	47	1186	92	831	115	1140	241
Fstl1	ENSMUSG00000022816	2281	246	7581	4030	2354	314	5589	1854
Fstl3	ENSMUSG00000020325	58	10	114	21	45	10	101	24

Fuca2	ENSMUSG00000019810	4743	786	5774	464	4738	302	6270	543
Fxyd5	ENSMUSG00000009687	283	49	603	81	271	56	473	80
Gab2	ENSMUSG00000004508	804	49	1077	134	805	51	1017	130
Gas2l3	ENSMUSG00000074802	49	17	117	44	58	17	123	41
Gdf6	ENSMUSG00000051279	21	6	60	18	20	4	52	18
Glipr2	ENSMUSG00000028480	116	26	213	33	120	25	192	33
Gm42417	ENSMUSG00000109510	601	184	1052	250	572	93	1085	285
Gm47302	ENSMUSG00000105211	24	9	110	97	27	2	68	30
Gm4739	ENSMUSG00000112808	128	31	209	69	125	23	223	29
Gng2	ENSMUSG00000043004	106	30	205	31	90	14	161	60
Gpr153	ENSMUSG00000042804	289	39	472	87	284	32	390	64
Gprc5b	ENSMUSG00000008734	417	88	644	60	371	45	622	68
Gpx1	ENSMUSG00000063856	1007	59	1482	189	1015	47	1424	176
Grb10	ENSMUSG00000020176	1827	116	2380	316	1880	141	2271	196
Grn	ENSMUSG00000034708	1349	120	1891	216	1368	123	1853	161
Gtse1	ENSMUSG00000022385	12	4	37	7	11	3	35	14
Gusb	ENSMUSG00000025534	484	36	644	46	462	48	631	40
Haspin	ENSMUSG00000050107	7	3	26	4	7	2	20	10
Hcls1	ENSMUSG00000022831	234	38	352	48	191	20	308	52
Hectd2os	ENSMUSG00000087579	181	28	259	45	192	32	288	75
Hells	ENSMUSG00000025001	29	11	78	18	36	7	79	13
Hhipl1	ENSMUSG00000021260	67	22	127	40	63	22	109	33
Hist1h2ap	ENSMUSG00000094777	37	19	118	39	33	8	97	65
Hmmr	ENSMUSG00000020330	21	9	98	40	21	6	82	33
Hspa1l	ENSMUSG00000007033	134	25	210	54	130	20	216	41
Ifi204	ENSMUSG00000073489	206	34	433	95	196	40	334	43
Ifi27l2a	ENSMUSG00000079017	237	35	439	84	218	39	420	49
Ifitm2	ENSMUSG00000060591	875	131	1176	80	841	117	1097	135
Ifitm3	ENSMUSG00000025492	1226	258	1689	136	1126	147	1515	182
Ifngr1	ENSMUSG00000020009	1109	126	1400	78	1101	128	1324	109
Ift122	ENSMUSG00000030323	278	32	496	94	309	39	488	78
Igfbp7	ENSMUSG00000036256	2248	254	4619	1238	2198	173	3722	608
Igsf6	ENSMUSG00000035004	49	7	92	27	46	7	79	10
Il10ra	ENSMUSG00000032089	138	27	237	86	108	21	194	46
Il1rl2	ENSMUSG00000070942	95	8	139	17	75	7	130	22
Il2rg	ENSMUSG00000031304	249	26	354	25	214	37	325	48
Il4ra	ENSMUSG00000030748	359	66	598	83	333	48	463	59
Incenp	ENSMUSG00000024660	92	17	144	29	81	14	144	40
Inhba	ENSMUSG00000041324	65	15	199	57	61	18	178	55
Iqgap1	ENSMUSG00000030536	1656	342	2416	368	1438	279	2181	405
Iqgap3	ENSMUSG00000028068	31	20	115	32	27	10	119	46
Irf7	ENSMUSG00000025498	220	65	413	114	189	26	332	52
Itga5	ENSMUSG00000000555	986	110	1409	68	945	123	1366	142
Itga9	ENSMUSG00000039115	1864	139	2467	423	1953	131	2644	386
Itgam	ENSMUSG00000030786	190	26	365	31	200	68	269	58
Itih5	ENSMUSG00000025780	368	63	749	273	377	54	597	122
Itpripl2	ENSMUSG00000095115	1256	118	1751	114	1222	89	1508	115
Kcne4	ENSMUSG00000047330	64	17	119	36	59	9	95	7
Kctd11	ENSMUSG00000046731	99	17	174	7	96	16	157	31
Kctd17	ENSMUSG00000033287	312	32	468	35	317	29	437	78
Kif11	ENSMUSG00000012443	54	18	219	84	56	14	169	71
Kif15	ENSMUSG00000036768	14	6	40	11	16	8	57	20
Kif18b	ENSMUSG00000051378	13	3	50	17	14	3	49	16
Kif20a	ENSMUSG00000003779	45	12	135	43	44	5	120	29
Kif22	ENSMUSG00000030677	26	8	61	17	19	10	51	20
Kif23	ENSMUSG00000032254	53	21	153	63	39	12	144	59
Kif2c	ENSMUSG00000028678	15	5	39	8	8	2	33	12
Kif4	ENSMUSG00000034311	35	11	86	21	32	13	86	26
Kif5b	ENSMUSG00000006740	4524	195	5272	357	4610	339	5543	496
Kifc1	ENSMUSG00000079553	21	17	44	7	18	7	48	18
Knl1	ENSMUSG00000027326	29	18	66	14	21	8	59	16
Knstrn	ENSMUSG00000027331	28	8	90	28	24	3	76	21
Kntc1	ENSMUSG00000029414	13	8	61	43	19	11	63	21
Lama4	ENSMUSG00000019846	2464	138	3226	192	2291	264	3050	270
Lamb1	ENSMUSG00000002900	2656	308	3500	191	2690	265	3282	467
Lamc1	ENSMUSG00000026478	4969	490	7157	380	5155	283	6688	486
Laptm5	ENSMUSG00000028581	500	82	885	205	478	56	708	122

Lcp1	ENSMUSG00000021998	803	118	1421	200	781	112	1229	174
Lgals3	ENSMUSG00000050335	56	18	203	128	57	20	148	73
Lgals3bp	ENSMUSG00000033880	711	128	1195	145	721	83	1079	113
Lgals9	ENSMUSG00000001123	575	96	870	73	533	82	766	58
Lhfpl2	ENSMUSG00000045312	121	17	351	198	128	21	269	96
Lilr4b	ENSMUSG00000112023	154	32	309	106	138	41	252	97
Litaf	ENSMUSG00000022500	341	97	525	67	315	74	445	72
Lman1l	ENSMUSG00000056271	6	2	21	5	6	5	23	11
Lockd	ENSMUSG00000098318	4	4	22	11	5	3	21	12
Loxl1	ENSMUSG00000032334	618	97	1447	604	687	57	1181	257
Loxl2	ENSMUSG00000034205	724	133	1635	127	683	84	1403	383
Lrp1	ENSMUSG00000040249	4010	495	5574	985	4250	358	5472	456
Lrp8	ENSMUSG00000028613	9	5	40	13	13	7	51	44
Ly6e	ENSMUSG00000022587	2929	530	3882	293	2782	305	3608	375
Ly86	ENSMUSG00000021423	93	6	221	112	92	20	181	43
Lyz2	ENSMUSG00000069516	4037	355	6768	1318	3852	675	5981	848
Mall	ENSMUSG00000027377	160	25	233	27	157	20	226	33
Map1b	ENSMUSG00000052727	582	102	873	51	444	82	777	113
Map4k4	ENSMUSG00000026074	3034	462	3619	383	2841	174	3784	234
Marcksl1	ENSMUSG00000047945	104	18	211	70	107	9	183	4
Masp1	ENSMUSG00000022887	205	16	307	114	219	36	315	61
Mcam	ENSMUSG00000032135	1064	99	1678	301	970	129	1515	234
Medag	ENSMUSG00000029659	437	58	771	121	486	78	684	41
Melk	ENSMUSG00000035683	19	16	45	19	13	4	40	14
Meox1	ENSMUSG00000001493	296	46	905	284	323	49	767	246
Mest	ENSMUSG00000051855	193	22	598	325	156	26	417	154
Mfap4	ENSMUSG00000042436	202	18	1249	1153	245	36	712	324
Mfap5	ENSMUSG00000030116	411	43	1634	1033	404	45	1132	430
Mis18bp1	ENSMUSG00000047534	15	3	59	20	16	6	44	14
Mki67	ENSMUSG00000031004	305	108	1186	340	284	62	1009	362
Mmp2	ENSMUSG00000031740	991	124	2606	1435	1013	93	2013	656
Mmp23	ENSMUSG00000029061	80	8	179	97	80	19	146	38
Mpp1	ENSMUSG00000031402	302	30	434	51	294	38	387	21
Mrc1	ENSMUSG00000026712	1125	59	1641	55	1195	105	1622	319
Ms4a6b	ENSMUSG00000024677	163	35	265	79	109	27	237	58
Ms4a6c	ENSMUSG00000079419	117	24	273	106	108	30	198	50
Msn	ENSMUSG00000031207	4381	327	6174	266	4286	400	5734	498
Msr1	ENSMUSG00000025044	91	16	165	30	95	12	158	35
Mxd3	ENSMUSG00000021485	3	2	21	8	5	2	23	10
Mxra7	ENSMUSG00000020814	353	43	549	114	334	33	485	81
Mybl2	ENSMUSG00000017861	12	4	32	11	10	3	29	7
Mybpc2	ENSMUSG00000038670	292	36	901	354	321	110	773	309
Myh10	ENSMUSG00000020900	964	132	1462	186	988	96	1366	213
Myl1	ENSMUSG00000061816	777	84	1354	639	674	113	1186	282
Myl6	ENSMUSG00000090841	3039	174	4606	743	2959	365	4098	492
Myo1d	ENSMUSG00000035441	367	44	489	47	360	56	474	39
Myo1f	ENSMUSG00000024300	97	11	170	34	88	10	163	43
Myof	ENSMUSG00000048612	346	89	640	167	312	56	541	78
Nbl1	ENSMUSG00000041120	214	26	349	63	215	19	307	61
Ncapg	ENSMUSG00000015880	17	11	88	26	18	10	55	17
Ncapg2	ENSMUSG00000042029	78	9	184	50	101	31	166	54
Ncaph	ENSMUSG00000034906	32	7	80	27	35	11	78	26
Ncf1	ENSMUSG00000015950	112	33	201	54	124	31	190	42
Nckap1l	ENSMUSG00000022488	260	42	511	159	240	40	412	73
Ndc80	ENSMUSG00000024056	18	8	69	23	19	4	56	15
Necap2	ENSMUSG00000028923	266	29	348	33	232	24	305	28
Nek2	ENSMUSG00000026622	16	10	60	22	15	3	46	11
Nes	ENSMUSG00000004891	1779	216	2677	688	1621	280	2378	347
Nid1	ENSMUSG00000005397	3926	530	6347	1387	3634	332	5323	725
Nid2	ENSMUSG00000021806	561	130	914	118	537	62	895	114
Nkd2	ENSMUSG00000021567	39	11	118	51	36	6	77	24
Nlrc3	ENSMUSG00000049871	58	17	139	45	66	12	140	43
Nppa	ENSMUSG00000041616	2919	1471	17568	21348	2998	848	11493	6461
Nppb	ENSMUSG00000029019	3896	1226	10218	3100	4287	995	11088	3450
Nuf2	ENSMUSG00000026683	21	9	86	40	16	4	77	34
Oaf	ENSMUSG00000032014	264	77	399	146	208	41	331	55
Olfml3	ENSMUSG00000027848	303	52	492	108	323	21	470	29

Otulin	ENSMUSG00000046034	372	28	492	30	374	38	498	73
P2ry6	ENSMUSG00000048779	103	15	191	33	95	15	152	28
P3h3	ENSMUSG00000023191	185	14	319	100	179	28	278	54
Pabpc1	ENSMUSG00000022283	2750	396	4424	589	2940	313	3983	663
Pam	ENSMUSG00000026335	13364	1232	16353	2118	13089	520	17039	1382
Pamr1	ENSMUSG00000027188	50	16	271	244	44	23	183	117
Pbk	ENSMUSG00000022033	18	15	71	30	16	9	69	28
Pcdhgc3	ENSMUSG00000102918	783	97	981	69	752	51	988	95
Pclaf	ENSMUSG00000040204	20	11	70	26	13	4	61	24
Pcolce	ENSMUSG00000029718	778	81	1236	178	675	91	1133	173
Pea15a	ENSMUSG00000013698	2146	275	2690	102	2035	242	2530	274
Pfkp	ENSMUSG00000021196	1083	101	1607	246	1116	108	1508	216
Pfn1	ENSMUSG00000018293	2349	50	2712	75	2362	104	2642	191
Phf11d	ENSMUSG00000068245	263	46	403	78	242	30	341	41
Phlda3	ENSMUSG00000041801	128	18	222	62	128	4	216	43
Pi16	ENSMUSG00000024011	1042	154	1987	337	1030	148	1793	499
Picalm	ENSMUSG00000039361	3682	311	4950	606	3495	243	4537	508
Pimreg	ENSMUSG00000020808	18	10	79	24	18	4	67	18
Pirb	ENSMUSG00000058818	142	22	283	84	134	30	266	92
Pla2g4a	ENSMUSG00000056220	118	46	201	50	118	39	188	22
Pla2g7	ENSMUSG00000023913	171	21	280	45	172	34	252	45
Plat	ENSMUSG00000031538	475	62	725	65	464	41	699	128
Plcg2	ENSMUSG00000034330	248	18	352	61	256	18	349	46
Plek	ENSMUSG00000020120	194	26	382	104	207	35	336	73
Plekhg2	ENSMUSG00000037552	415	79	602	42	355	76	498	81
Plekhh2	ENSMUSG00000040852	172	20	250	25	168	11	228	26
Plekho1	ENSMUSG00000015745	873	48	1229	109	927	54	1262	202
Plekho2	ENSMUSG00000050721	497	98	707	90	525	90	666	108
Pls3	ENSMUSG00000016382	1718	79	2436	255	1640	157	2091	235
Pmepa1	ENSMUSG00000038400	495	93	798	197	520	37	753	189
Pmp22	ENSMUSG00000018217	799	98	1125	49	732	79	1029	87
Postn	ENSMUSG00000027750	1288	354	16369	19036	1325	317	10262	8108
Ppic	ENSMUSG00000024538	424	29	954	416	413	41	706	107
Ppp1r9b	ENSMUSG00000038976	1114	96	1479	122	1143	53	1390	134
Praf2	ENSMUSG00000031149	91	14	155	27	90	17	137	19
Prc1	ENSMUSG00000038943	59	10	277	77	74	20	211	83
Prcp	ENSMUSG00000061119	378	31	583	113	369	43	525	62
Prelid1	ENSMUSG00000021486	540	47	753	58	476	49	647	93
Prnd	ENSMUSG00000027338	170	30	416	121	167	33	335	59
Prr11	ENSMUSG00000020493	26	9	79	21	18	6	65	19
Prrg3	ENSMUSG00000033361	304	49	476	60	309	45	455	53
Psat1	ENSMUSG00000024640	65	15	115	35	66	18	116	28
Psrc1	ENSMUSG00000068744	10	4	29	15	9	4	29	18
Ptgfrn	ENSMUSG00000027864	1116	158	1460	136	1089	77	1442	156
Ptgis	ENSMUSG00000017969	202	28	364	114	200	27	323	64
Ptma	ENSMUSG00000026238	4087	376	5328	376	3885	518	4897	607
Ptprj	ENSMUSG00000025314	404	60	631	75	427	56	571	83
Pxdn	ENSMUSG00000020674	2049	186	2790	214	2026	252	2556	286
Qsox1	ENSMUSG00000033684	551	17	792	82	588	46	765	102
Rab13	ENSMUSG00000027935	64	9	113	32	60	13	95	9
Rab31	ENSMUSG00000056515	515	47	841	272	486	52	743	141
Rab7b	ENSMUSG00000052688	114	17	216	47	113	25	187	28
Racgap1	ENSMUSG00000023015	51	13	168	39	47	22	147	53
Rbl1	ENSMUSG00000027641	92	18	142	31	88	10	137	16
Rcan1	ENSMUSG00000022951	2025	588	3577	1067	2237	187	3873	1294
Rcc2	ENSMUSG00000040945	364	24	453	17	354	21	462	57
Rcn3	ENSMUSG00000019539	304	49	526	177	289	35	431	74
Rgs16	ENSMUSG00000026475	42	13	97	25	41	22	76	17
Rhoc	ENSMUSG00000002233	1016	82	1471	147	963	60	1399	180
Ripk1	ENSMUSG00000021408	280	44	414	37	283	47	366	18
Rnase4	ENSMUSG00000021876	967	55	1292	169	990	104	1212	120
Rnd3	ENSMUSG00000017144	446	50	573	42	410	17	542	43
Rnf213	ENSMUSG00000070327	1921	265	2664	212	1732	232	2287	172
Rnf4	ENSMUSG00000029110	719	66	881	34	682	79	832	58
Rrm2	ENSMUSG00000020649	47	3	106	36	46	11	90	24
Rsad2	ENSMUSG00000020641	558	59	808	127	569	74	833	251
Rsu1	ENSMUSG00000026727	628	30	848	58	638	25	765	30

Runx3	ENSMUSG00000070691	15	6	40	18	11	6	33	8
S100a10	ENSMUSG00000041959	648	48	917	65	558	90	873	124
S100a11	ENSMUSG00000027907	541	79	1009	138	513	59	798	136
S100a4	ENSMUSG00000001020	101	23	185	48	95	20	157	48
S100a6	ENSMUSG00000001025	463	35	675	47	446	74	610	100
Samd9l	ENSMUSG00000047735	766	77	1115	179	714	95	898	111
Scml4	ENSMUSG00000044770	68	22	133	24	73	13	155	38
Sdc3	ENSMUSG00000025743	1679	201	2152	200	1603	123	1955	125
Sec61a1	ENSMUSG00000030082	772	59	1001	99	760	61	922	32
Sema3f	ENSMUSG00000034684	281	62	437	37	248	21	450	63
Serpinb1c	ENSMUSG00000079049	2	1	40	44	2	1	21	10
Serpinf1	ENSMUSG00000000753	424	109	1021	469	419	39	783	209
Sgo1	ENSMUSG00000023940	10	3	30	10	11	4	27	6
Sh3bgrl3	ENSMUSG00000028843	283	45	439	75	294	39	393	63
Sh3bp2	ENSMUSG00000054520	69	26	114	31	61	7	106	12
Sh3pxd2b	ENSMUSG00000040711	249	93	530	157	247	52	411	85
Siglec1	ENSMUSG00000027322	120	21	202	53	128	32	195	28
Slamf9	ENSMUSG00000026548	102	15	214	66	105	27	174	36
Slc7a5	ENSMUSG00000040010	92	11	140	11	101	4	149	21
Slfn2	ENSMUSG00000072620	233	30	361	80	223	50	310	16
Slfn9	ENSMUSG00000069793	117	11	264	62	100	26	248	59
Slmap	ENSMUSG00000021870	4454	592	5686	709	4379	533	6028	274
Smc2	ENSMUSG00000028312	165	38	318	105	129	27	242	61
Snca	ENSMUSG00000025889	79	27	187	57	121	41	270	139
Sntb2	ENSMUSG00000041308	632	76	913	145	638	68	853	99
Socs3	ENSMUSG00000053113	83	19	176	67	83	9	171	89
Sox9	ENSMUSG00000000567	49	4	128	64	53	5	103	17
Spag5	ENSMUSG00000002055	26	31	54	13	19	10	55	17
Sparc	ENSMUSG00000018593	7512	635	18831	6579	7355	933	14409	2942
Spc25	ENSMUSG00000005233	14	8	61	17	18	8	51	25
Spdl1	ENSMUSG00000069910	14	4	33	8	11	3	28	11
Specc1	ENSMUSG00000042331	92	10	188	35	117	18	192	33
Sprr1a	ENSMUSG00000050359	2	1	59	85	1	1	31	38
Sptlc2	ENSMUSG00000021036	532	46	684	85	500	56	646	54
Sri	ENSMUSG00000003161	820	36	947	43	794	58	937	33
Srpx2	ENSMUSG00000031253	164	22	353	215	151	23	300	80
Ssc5d	ENSMUSG00000035279	158	37	405	221	163	32	347	116
Stab1	ENSMUSG00000042286	1603	325	2076	219	1569	318	2072	151
Stc1	ENSMUSG00000014813	72	10	132	17	71	21	124	24
Stil	ENSMUSG00000028718	13	7	37	11	18	15	36	14
Stmn1	ENSMUSG00000028832	255	12	400	110	218	34	386	85
Sulf1	ENSMUSG00000016918	588	72	1266	375	636	106	916	142
Svep1	ENSMUSG00000028369	454	54	1081	419	488	75	1015	246
Syk	ENSMUSG00000021457	661	54	816	97	624	37	818	114
Synpo2l	ENSMUSG00000039376	2429	160	4589	1397	2488	266	4960	1191
Tacc3	ENSMUSG00000037313	55	17	109	27	41	13	101	32
Tagln2	ENSMUSG00000026547	1222	205	1863	181	1131	206	1710	250
Tax1bp3	ENSMUSG00000040158	636	52	758	26	603	49	731	37
Tcf19	ENSMUSG00000050410	39	7	78	17	36	10	69	16
Tead2	ENSMUSG00000030796	87	19	131	19	76	18	119	12
Tgfb1	ENSMUSG00000002603	550	90	766	73	584	72	754	96
Tgfbi	ENSMUSG00000035493	530	77	732	148	489	68	651	78
Tgif1	ENSMUSG00000047407	80	8	172	68	80	12	125	17
Thbs1	ENSMUSG00000040152	409	149	2336	862	430	59	1679	388
Thbs3	ENSMUSG00000028047	103	23	225	128	92	40	196	95
Thbs4	ENSMUSG00000021702	98	24	980	1112	104	23	729	572
Thy1	ENSMUSG00000032011	161	30	284	38	162	24	265	26
Timp1	ENSMUSG00000001131	29	17	298	217	27	8	170	133
Timp2	ENSMUSG00000017466	2020	279	2851	462	2083	233	2707	361
Tk1	ENSMUSG00000025574	27	6	65	23	23	5	56	16
Tln1	ENSMUSG00000028465	3433	355	4129	304	3528	301	4361	274
Tlr13	ENSMUSG00000033777	67	17	156	73	54	17	129	34
Tlr4	ENSMUSG00000039005	491	150	724	86	471	25	747	193
Tmem173	ENSMUSG00000024349	169	26	277	47	150	31	216	31
Tmem176b	ENSMUSG00000029810	447	59	695	169	422	18	612	87
Tmem254b	ENSMUSG00000021867	94	24	152	22	90	34	151	21
Tmsb10	ENSMUSG00000079523	1059	131	1943	416	1045	141	1515	201

Tmsb4x	ENSMUSG00000049775	6492	390	9057	1900	6337	792	8278	983
Tnc	ENSMUSG00000028364	43	6	544	443	40	8	407	402
Tnfaip6	ENSMUSG00000053475	16	7	49	21	15	3	40	15
Tnfaip8l1	ENSMUSG00000044469	59	9	106	14	51	10	88	20
Tnfrsf11b	ENSMUSG00000063727	4	4	30	9	8	3	25	12
Tnfrsf12a	ENSMUSG00000023905	569	211	1115	309	580	83	1245	254
Tnfrsf1a	ENSMUSG00000030341	862	76	1114	56	808	94	1077	63
Top2a	ENSMUSG00000020914	156	64	602	224	141	28	511	197
Tpm2	ENSMUSG00000028464	338	60	565	58	358	58	477	79
Tpm3	ENSMUSG00000027940	1515	192	2133	211	1390	166	1854	103
Tpm4	ENSMUSG00000031799	3181	181	4770	635	3020	369	4360	322
Tpx2	ENSMUSG00000027469	56	21	186	55	49	21	165	46
Trem2	ENSMUSG00000023992	35	6	92	48	34	8	74	19
Trim47	ENSMUSG00000020773	322	56	432	50	294	39	391	53
Trim59	ENSMUSG00000034317	45	13	121	42	39	10	82	19
Tspan6	ENSMUSG00000067377	201	18	351	99	188	36	291	45
Ttc9	ENSMUSG00000042734	48	13	109	12	46	8	91	22
Ttk	ENSMUSG00000038379	11	6	50	21	9	2	35	14
Tuba1a	ENSMUSG00000072235	1839	223	2349	176	1762	189	2229	379
Tubb5	ENSMUSG00000001525	1852	114	2383	316	1680	188	2353	298
Tyms	ENSMUSG00000025747	54	5	125	33	37	11	97	35
Tyrobp	ENSMUSG00000030579	196	25	385	134	191	36	314	57
Ube2c	ENSMUSG00000001403	26	8	84	26	25	10	76	27
Uck2	ENSMUSG00000026558	821	87	1664	260	843	95	1668	393
Ugt1a7c	ENSMUSG00000090124	31	6	81	25	36	7	76	31
Uhrf1	ENSMUSG00000001228	52	9	132	55	40	18	111	47
Ulbp1	ENSMUSG00000079685	96	27	174	24	75	13	122	11
Unc93b1	ENSMUSG00000036908	322	73	550	131	355	31	500	38
Vat1	ENSMUSG00000034993	610	35	884	140	594	35	798	66
Vcan	ENSMUSG00000021614	730	115	1503	151	665	105	1354	355
Vim	ENSMUSG00000026728	3763	407	7380	1381	3521	520	6206	983
Xirp2	ENSMUSG00000027022	23026	1933	46990	12463	24085	3291	47862	16560
Xylt1	ENSMUSG00000030657	76	8	167	19	86	14	139	34
Ywhaz	ENSMUSG00000022285	2464	152	2960	156	2391	141	2749	56
Zyx	ENSMUSG00000029860	896	129	1214	76	876	143	1120	93

Supplementary Table S4. RNASeq analysis of effects of angiotensin II (AngII) on mRNA expression in hearts from PKN2Het vs WT littermates: mRNAs significantly downregulated by AngII in PKN2Het or WT hearts.

Gene Symbol	Ensembl gene id	WT Vehicle		WT AngII		PKN2Het Vehicle		PKN2Het AngII	
		Mean	SD	Mean	SD	Mean	SD	Mean	SD
March6	ENSMUSG00000039100	4345	470	3364	215	4521	533	3717	463
A530016L24Rik	ENSMUSG00000043122	486	59	252	64	537	84	302	85
Abca12	ENSMUSG00000050296	186	25	118	22	190	14	113	26
Abcc9	ENSMUSG00000030249	9613	652	6487	953	9739	961	7503	994
Acad11	ENSMUSG00000090150	4332	376	3012	486	4068	456	3113	804
Acss1	ENSMUSG00000027452	6051	350	3795	623	6116	511	4694	536
Adcy9	ENSMUSG00000005580	523	37	386	56	548	56	435	28
Adi1	ENSMUSG00000020629	777	43	587	41	770	25	625	82
Adra1a	ENSMUSG00000045875	454	44	309	38	449	28	324	43
Adrb1	ENSMUSG00000035283	299	27	186	24	312	44	237	7
Aes	ENSMUSG00000054452	8100	309	6557	669	8573	728	7022	440
Ak4	ENSMUSG00000028527	1110	101	673	88	1095	120	841	121
Aldh2	ENSMUSG00000029455	3628	278	2621	303	3753	239	3023	178
Aldh4a1	ENSMUSG00000028737	2051	114	1277	213	2215	312	1569	163
Aldh6a1	ENSMUSG00000021238	4712	450	3354	316	4858	561	3659	653
Aldob	ENSMUSG00000028307	153	33	37	15	130	33	51	29
Angpt1	ENSMUSG00000022309	983	254	585	130	825	181	510	175
Ano10	ENSMUSG00000037949	698	182	344	58	669	131	405	96
Apbb1	ENSMUSG00000037032	1485	207	973	270	1582	132	1096	145
Arel1	ENSMUSG00000042350	1385	91	997	117	1373	120	1084	87
Arfgef1	ENSMUSG00000067851	4031	401	3145	313	4160	428	3482	356
Asb10	ENSMUSG00000038204	1171	63	812	130	1209	144	936	72
Asb14	ENSMUSG00000021898	1693	190	1270	203	1859	69	1299	90
Asb15	ENSMUSG00000029685	1620	111	997	132	1719	167	1139	111
Atp2a2	ENSMUSG00000029467	276075	9196	182185	30616	276406	28699	212684	20219
Bckdha	ENSMUSG00000060376	3198	271	2027	281	3491	476	2369	149
Bckdhb	ENSMUSG00000032263	843	53	580	105	820	64	644	127
Blcap	ENSMUSG00000067787	674	60	518	51	708	57	566	19
Cacna1s	ENSMUSG00000026407	289	41	167	50	318	106	207	31
Calcoco1	ENSMUSG00000023055	2244	90	1682	135	2260	208	1798	84
Camk2a	ENSMUSG00000024617	894	42	584	78	907	103	635	63
Cbx7	ENSMUSG00000053411	375	36	283	55	434	66	306	28
Cdnf	ENSMUSG00000039496	693	83	462	69	638	86	470	25
Clasp1	ENSMUSG00000064302	10042	1348	7016	1532	10721	1184	8158	674
Clasp2	ENSMUSG00000033392	1555	68	1255	49	1588	122	1315	122
Clcn1	ENSMUSG00000029862	122	31	56	16	126	19	52	17
Clpx	ENSMUSG00000015357	2210	180	1776	257	2247	186	1932	167
Cmtm8	ENSMUSG00000041012	193	16	122	26	198	19	133	20
Cmya5	ENSMUSG00000047419	30807	1640	21498	2766	31721	3064	25206	2497
Cngb3	ENSMUSG00000056494	85	21	34	5	96	10	48	18
Cnst	ENSMUSG00000038949	1417	109	1052	101	1496	138	1149	127
Coq8a	ENSMUSG00000026489	8867	811	6241	1021	9606	1491	7168	332
Cpeb3	ENSMUSG00000039652	1712	172	1204	231	1874	247	1337	250
Creg1	ENSMUSG00000040713	2146	83	1671	135	2006	90	1667	106
Crip2	ENSMUSG00000006356	12559	568	9239	1165	12739	1064	10202	689
D10Jhu81e	ENSMUSG00000053329	4564	257	3218	428	4591	348	3636	306
Dcaf11	ENSMUSG00000022214	2685	174	1996	224	2643	170	2142	182
Dcaf8	ENSMUSG00000026554	3228	148	2835	139	3162	236	2954	198
Dcun1d2	ENSMUSG00000038506	1528	101	1151	175	1592	86	1245	86
Dglucy	ENSMUSG00000021185	1025	27	630	95	1021	154	706	146
Dhdh	ENSMUSG00000011382	539	37	412	40	541	58	441	60
Dsg2	ENSMUSG00000044393	2214	147	1452	293	2105	275	1573	277
Ehhadh	ENSMUSG00000022853	319	54	215	36	367	52	263	31
Entpd5	ENSMUSG00000021236	4587	555	2853	380	4318	388	3001	409
Epha4	ENSMUSG00000026235	1430	183	778	139	1532	195	924	149
Esrra	ENSMUSG00000024955	1498	69	1122	160	1612	352	1242	55
Fam174b	ENSMUSG00000078670	5146	309	3201	609	5011	412	3808	373
Fblim1	ENSMUSG00000006219	4020	400	2963	478	4442	528	3364	287

Fgf1	ENSMUSG00000036585	4273	125	2800	289	4103	316	3148	422
Fgf13	ENSMUSG00000031137	912	28	624	84	957	100	687	122
Fgf16	ENSMUSG00000031230	350	35	197	36	325	28	212	47
Fitm2	ENSMUSG00000048486	4343	253	2687	652	4389	466	3182	252
Fktn	ENSMUSG00000028414	919	77	713	82	966	86	734	38
Fyco1	ENSMUSG00000025241	8256	626	6068	819	8149	1328	6602	1438
Gadd45a	ENSMUSG00000036390	237	30	173	8	280	38	199	29
Gal3st3	ENSMUSG00000047658	235	33	123	31	253	42	156	27
Gcat	ENSMUSG00000006378	139	15	88	16	122	23	81	20
Gcdh	ENSMUSG00000003809	1192	92	826	152	1218	47	907	64
Ghr	ENSMUSG00000055737	3256	90	2495	135	3239	69	2574	165
Gid4	ENSMUSG00000018415	1429	66	1151	186	1528	101	1208	39
Gm10435	ENSMUSG00000072902	350	47	240	57	374	40	256	85
Gm10635	ENSMUSG00000111765	62	15	26	11	57	10	29	5
Gm37691	ENSMUSG00000104348	120	14	62	18	123	24	83	16
Gpd1l	ENSMUSG00000050627	1532	131	1271	72	1610	184	1401	84
Gpt2	ENSMUSG00000031700	649	55	444	64	689	85	483	73
Gramd1b	ENSMUSG00000040111	802	117	546	52	929	115	696	158
Grcc10	ENSMUSG00000072772	1142	101	905	96	1137	51	918	108
Grm1	ENSMUSG00000019828	796	113	605	58	918	161	761	121
Gstm2	ENSMUSG00000040562	1088	63	815	54	1016	120	792	38
Hadha	ENSMUSG00000025745	29451	2086	19576	3952	28687	2036	22309	1220
Hdac11	ENSMUSG00000034245	385	30	247	18	404	32	306	52
Hdlbp	ENSMUSG00000034088	16562	1228	12760	1097	16946	1402	13742	294
Herpud1	ENSMUSG00000031770	2271	311	1495	173	2164	218	1572	242
Idh3g	ENSMUSG00000002010	6376	459	4785	580	6418	422	4954	479
Ift81	ENSMUSG00000029469	1002	129	666	78	994	49	708	78
Il15	ENSMUSG00000031712	439	60	265	38	422	29	277	62
Inmt	ENSMUSG00000003477	120	30	62	9	125	27	60	31
Iqsec1	ENSMUSG00000034312	2343	237	1649	239	2475	375	1887	147
Isoc1	ENSMUSG00000024601	1090	72	835	76	1154	85	886	108
Ivd	ENSMUSG00000027332	5467	219	3645	686	5738	467	4074	283
Kcnd2	ENSMUSG00000060882	633	53	397	84	534	96	384	46
Kcnj11	ENSMUSG00000096146	2367	103	1635	312	2506	312	1913	137
Kcnj12	ENSMUSG00000042529	362	32	215	47	369	42	263	36
Kcnj3	ENSMUSG00000026824	1558	124	892	227	1560	194	966	194
Kcnj5	ENSMUSG00000032034	1526	103	978	122	1446	143	1137	106
Kcnv2	ENSMUSG00000047298	217	40	101	27	221	15	94	19
Klf15	ENSMUSG00000030087	629	39	379	62	616	39	429	68
Klhdc1	ENSMUSG00000051890	679	63	505	66	737	123	482	72
Klhdc7a	ENSMUSG00000078234	208	40	115	26	215	51	132	33
Klhl24	ENSMUSG00000062901	8344	685	6893	406	8712	1112	7374	755
Klhl30	ENSMUSG00000026308	803	90	596	82	845	153	665	56
Klhl38	ENSMUSG00000022357	594	49	396	86	598	80	398	64
Ldhd	ENSMUSG00000031958	690	79	434	51	734	70	479	49
Lgals4	ENSMUSG00000053964	258	38	159	45	261	30	142	30
Lrrc14b	ENSMUSG00000021579	1627	72	1181	214	1726	119	1267	58
Lrtm1	ENSMUSG00000045776	10990	1420	7948	2173	9808	949	7556	656
Macrod1	ENSMUSG00000036278	1985	82	1475	206	2115	228	1644	157
Maob	ENSMUSG00000040147	1029	117	683	136	1003	67	758	145
Mccc2	ENSMUSG00000021646	1044	126	719	104	1066	137	833	122
Me3	ENSMUSG00000030621	1244	36	938	86	1271	81	1046	52
Mfap3l	ENSMUSG00000031647	512	38	351	27	511	51	378	15
Mgea5	ENSMUSG00000025220	2744	156	2258	103	2825	202	2463	245
Mitf	ENSMUSG00000035158	1007	108	715	103	983	124	817	111
Mlycd	ENSMUSG00000074064	1449	99	1002	186	1535	222	1119	122
Mmab	ENSMUSG00000029575	681	48	453	44	638	56	508	62
Mrgprh	ENSMUSG00000059408	120	15	59	19	121	13	67	15
mt-Rnr2	ENSMUSG00000064339	356240	61989	247940	69255	362883	53161	276407	38517
Mut	ENSMUSG00000023921	2916	284	2169	324	2826	245	2373	210
Mylk3	ENSMUSG00000031698	13367	1180	7949	948	12901	1032	9303	1403
Nadk2	ENSMUSG00000022253	1247	102	792	168	1254	222	893	160
Nceh1	ENSMUSG00000027698	4775	531	3631	517	4935	459	4158	196
Nipsnap2	ENSMUSG00000029432	12499	468	8522	1242	13021	796	9974	916
Osbp2	ENSMUSG00000020435	538	31	388	52	578	65	451	35
Oxr1	ENSMUSG00000022307	1785	149	1475	130	1747	115	1456	95
Oxsm	ENSMUSG00000021786	592	47	470	26	633	71	520	20

Oxsr1	ENSMUSG00000036737	1469	64	1253	72	1563	110	1374	101
P2ry1	ENSMUSG00000027765	592	33	336	93	614	60	420	44
Pank1	ENSMUSG00000033610	1036	54	700	77	1098	87	753	63
Paqr9	ENSMUSG00000064225	1387	188	959	139	1468	178	1110	132
Pcca	ENSMUSG00000041650	1754	109	1194	210	1808	136	1417	61
Pcnt	ENSMUSG00000001151	1516	71	1137	205	1490	140	1194	68
Pde4a	ENSMUSG00000032177	1479	69	1032	200	1596	227	1163	137
Pde4d	ENSMUSG00000021699	566	55	373	47	575	76	416	43
Pdha1	ENSMUSG00000031299	24973	2883	19882	1983	24844	2183	21298	1610
Pdp1	ENSMUSG00000049225	1052	157	827	97	1038	87	873	99
Pdp2	ENSMUSG00000048371	822	182	433	87	839	116	560	111
Pdpr	ENSMUSG00000033624	2409	176	1771	254	2523	330	2118	147
Pex11a	ENSMUSG00000030545	362	28	251	44	360	53	277	16
Pfkfb1	ENSMUSG00000025271	181	54	76	15	125	29	76	31
Pink1	ENSMUSG00000028756	7908	460	5095	582	8315	930	5887	645
Pkia	ENSMUSG00000027499	6489	587	5105	667	6499	450	5564	198
Pkig	ENSMUSG00000035268	1911	58	1637	67	1943	106	1679	127
Pkm	ENSMUSG00000032294	19295	1479	15108	1318	19558	1824	16780	652
Pla2g5	ENSMUSG00000041193	703	60	323	118	617	100	370	76
Pln	ENSMUSG00000038583	103669	9371	75161	8133	106163	7666	81558	8775
Plxnb1	ENSMUSG00000053646	817	113	486	68	762	119	534	62
Pm20d2	ENSMUSG00000054659	458	21	337	55	500	64	387	32
Pnpla8	ENSMUSG00000036257	3505	336	2968	271	3603	216	3140	212
Ppargc1a	ENSMUSG00000029167	1975	310	1550	238	2310	255	1647	126
Ppfibp2	ENSMUSG00000036528	632	102	494	48	689	69	541	29
Ppip5k2	ENSMUSG00000040648	2823	288	1947	183	2670	258	1967	582
Ppm1l	ENSMUSG00000027784	1625	159	1031	184	1684	206	1260	149
Ppp1r14c	ENSMUSG00000040653	3104	380	2506	85	3332	336	2807	364
Pptc7	ENSMUSG00000038582	3687	299	2559	451	3939	504	3081	324
Prkab1	ENSMUSG00000029513	625	39	486	57	684	60	543	69
Rap1gap2	ENSMUSG00000038807	2208	274	1592	353	2292	294	1806	108
Rbfox1	ENSMUSG00000008658	634	47	360	59	657	70	426	78
Reep1	ENSMUSG00000052852	440	28	345	23	454	53	354	26
Reep5	ENSMUSG00000005873	6212	260	4792	333	6407	427	5212	316
Rgs2	ENSMUSG00000026360	831	133	501	64	860	81	573	166
Ric8b	ENSMUSG00000035620	1056	84	764	88	1036	119	844	32
Rilpl1	ENSMUSG00000029392	2712	176	2148	322	2829	181	2289	127
Rmnd5a	ENSMUSG00000002222	2488	67	2108	149	2641	123	2117	196
Rpl3l	ENSMUSG00000002500	3330	271	1888	478	3250	187	2275	264
Rtn2	ENSMUSG00000030401	979	45	649	92	997	112	733	89
Sdha	ENSMUSG00000021577	26565	1713	17836	2991	26775	2129	20539	1624
Sec31b	ENSMUSG00000051984	190	12	140	15	206	17	142	18
Selenbp1	ENSMUSG00000068874	1432	58	903	170	1401	157	1028	139
Sgcb	ENSMUSG00000029156	2725	146	2351	41	2817	190	2512	150
Slc20a2	ENSMUSG00000037656	3299	120	2345	335	3156	313	2538	136
Slc22a5	ENSMUSG00000018900	515	48	392	19	513	45	415	24
Slc25a34	ENSMUSG00000040740	2458	124	1716	276	2459	202	1859	71
Slc25a42	ENSMUSG00000002346	919	100	557	97	966	156	583	82
Slc27a1	ENSMUSG00000031808	1220	145	877	126	1202	129	879	108
Slc4a3	ENSMUSG00000006576	2856	245	2018	244	3026	328	2319	297
Smim20	ENSMUSG00000061461	843	73	641	62	853	63	664	59
Stom	ENSMUSG00000026880	2813	112	2237	204	2994	287	2470	159
Stum	ENSMUSG00000053963	160	19	64	17	122	38	54	18
Syde2	ENSMUSG00000036863	475	95	306	46	437	39	305	68
Synj2	ENSMUSG00000023805	1330	103	1004	60	1390	140	1030	43
Taf1a	ENSMUSG00000072258	316	29	238	17	323	11	239	23
Tbc1d10c	ENSMUSG00000040247	79	13	45	9	80	16	46	8
Tbc1d16	ENSMUSG00000039976	1680	162	1163	183	1773	217	1329	136
Tbc1d4	ENSMUSG00000033083	2228	153	1576	167	2172	256	1634	160
Tbx5	ENSMUSG00000018263	456	56	299	60	527	87	347	57
Tcea3	ENSMUSG00000001604	1429	35	1076	111	1549	116	1154	106
Tmem150c	ENSMUSG00000050640	201	25	109	24	249	43	146	26
Tmem182	ENSMUSG00000079588	4532	444	3422	366	4747	305	3856	392
Tmem245	ENSMUSG00000055296	3203	419	2375	169	3028	247	2485	165
Tmem63b	ENSMUSG00000036026	1741	102	1317	164	1858	169	1449	124
Tmem65	ENSMUSG00000062373	3732	474	2882	185	3814	575	3099	401
Tnfrsf19	ENSMUSG00000060548	97	27	54	15	91	22	56	12

Tnni3k	ENSMUSG00000040086	2347	262	1468	283	2247	145	1743	187
Trap1	ENSMUSG00000005981	2287	176	1864	73	2431	195	1969	70
Trim7	ENSMUSG00000040350	466	53	297	42	440	107	331	53
Trip10	ENSMUSG00000019487	1361	60	1155	94	1369	30	1142	93
Ttll1	ENSMUSG00000022442	921	104	531	110	965	54	680	86
Txlnb	ENSMUSG00000039891	15534	643	11828	883	16172	1476	12752	915
Uckl1os	ENSMUSG00000010492	61	16	21	7	56	24	31	10
Vldlr	ENSMUSG00000024924	9623	566	7598	786	10008	777	8275	415
Ybx2	ENSMUSG00000018554	158	19	86	33	158	21	98	24
Zfp612	ENSMUSG00000044676	301	27	215	18	304	9	201	39
Zyg11b	ENSMUSG00000034636	2665	223	2061	190	2682	291	2292	252

Supplementary Table S5. RNASeq analysis of effects of angiotensin II (AngII) on mRNA expression in hearts from PKN2Het vs WT littermates: mRNAs significantly upregulated by AngII in WT hearts.

Gene Symbol	Ensembl gene id	WT Vehicle		WT AngII		PKN2Het Vehicle		PKN2Het AngII	
		Mean	SD	Mean	SD	Mean	SD	Mean	SD
March1	ENSMUSG00000036469	56	11	114	66	64	15	90	21
1500011B03Rik	ENSMUSG00000072694	30	9	54	13	33	8	48	9
1500015O10Rik	ENSMUSG00000026051	3	2	49	80	5	3	18	14
4930503L19Rik	ENSMUSG00000044906	133	18	213	40	136	22	156	26
9930111J21Rik2	ENSMUSG00000069892	725	103	926	89	721	107	783	91
Abca9	ENSMUSG00000041797	849	55	1144	187	818	92	953	67
Abhd2	ENSMUSG00000039202	898	103	1060	61	933	139	1000	123
AC105304.1	ENSMUSG00000117110	1	2	19	19	2	2	11	8
Acp5	ENSMUSG00000001348	5	2	22	17	6	4	15	9
Actb	ENSMUSG00000029580	7471	1804	10178	1677	7763	1384	9365	1200
Actg1	ENSMUSG00000062825	6824	1047	8916	1194	7413	1015	8405	1266
Actg2	ENSMUSG00000059430	11	6	37	14	12	6	20	12
Actn4	ENSMUSG00000054808	2213	272	2742	211	2012	203	2417	171
Actr2	ENSMUSG00000020152	2099	200	2493	267	2101	71	2352	201
Adam10	ENSMUSG00000054693	1295	82	1540	84	1289	31	1416	75
Adamts4	ENSMUSG00000006403	26	22	120	29	38	22	83	38
Adgra2	ENSMUSG00000031486	286	38	404	66	291	29	400	70
Adss	ENSMUSG00000015961	296	25	391	63	284	33	321	15
Aebp1	ENSMUSG00000020473	297	22	678	352	334	100	516	123
Agrn	ENSMUSG00000041936	958	125	1295	154	947	79	1136	70
Ahnak2	ENSMUSG00000072812	300	55	611	330	327	47	473	130
Aida	ENSMUSG00000042901	767	113	1024	80	877	107	902	132
Akr1b8	ENSMUSG00000029762	67	13	106	31	68	10	93	26
Akt3	ENSMUSG00000019699	651	50	856	71	640	112	760	47
Aldh1a3	ENSMUSG00000015134	25	2	51	12	25	7	49	8
Amot	ENSMUSG00000041688	452	43	612	111	514	116	652	97
Antxr1	ENSMUSG00000033420	343	48	747	479	332	36	524	184
Anxa3	ENSMUSG00000029484	637	92	881	124	558	119	714	80
Anxa4	ENSMUSG00000029994	390	35	552	99	380	26	495	39
Anxa8	ENSMUSG00000021950	10	5	45	20	13	5	25	12
Aoah	ENSMUSG00000021322	51	20	105	50	48	14	83	23
Ap2b1	ENSMUSG00000035152	1179	64	1470	164	1223	59	1427	130
Ap3s1	ENSMUSG00000024480	226	22	364	126	251	27	316	37
Apaf1	ENSMUSG00000019979	202	42	332	75	213	32	293	28
Apobec1	ENSMUSG00000040613	119	28	241	144	120	8	163	31
Apobr	ENSMUSG00000042759	32	3	70	17	32	9	51	18
Apol11b	ENSMUSG00000091694	7	5	34	9	17	6	37	25
App	ENSMUSG00000022892	3981	381	4939	605	3820	129	4513	126
Aqp8	ENSMUSG00000030762	83	23	156	68	106	26	182	68
Arf3	ENSMUSG00000051853	700	31	853	47	714	16	777	34
Arfip1	ENSMUSG00000074513	425	63	585	108	425	15	463	26
Arhgap23	ENSMUSG00000049807	496	48	632	37	504	37	586	47
Arhgap45	ENSMUSG00000035697	124	18	203	68	131	23	172	35
Arhgdia	ENSMUSG00000025132	2274	183	2741	90	2286	149	2537	205
Arhgef2	ENSMUSG00000028059	885	125	1137	110	951	83	1051	107
Arhgef39	ENSMUSG00000051517	7	3	25	5	7	4	18	6
Armcx2	ENSMUSG00000033436	204	22	300	52	209	17	270	18
Arpc2	ENSMUSG00000006304	2337	43	2757	185	2338	47	2652	121
Arpin	ENSMUSG00000039043	239	19	299	19	258	25	280	26
Arrb2	ENSMUSG00000060216	182	31	313	100	186	36	253	55
Arsb	ENSMUSG00000042082	304	35	427	70	298	13	358	32
Aspn	ENSMUSG00000021388	750	159	3970	5012	798	171	1825	1043
Ass1	ENSMUSG00000076441	79	24	147	71	80	5	118	24
Atl3	ENSMUSG00000024759	741	53	1024	135	748	50	882	82
Atp6v0a4	ENSMUSG00000038600	9	7	42	23	11	7	25	11
Atp6v1h	ENSMUSG00000033793	486	36	596	56	470	28	561	47
Atp7a	ENSMUSG00000033792	198	11	253	13	197	21	209	17
Atp9a	ENSMUSG00000027546	1025	69	1233	124	1046	42	1120	130
AW551984	ENSMUSG00000038112	5	2	17	8	7	4	17	10

B2m	ENSMUSG00000060802	4421	1037	6550	1100	4422	785	4929	246
B3galt2	ENSMUSG00000033849	372	49	542	57	383	37	471	157
B4galnt1	ENSMUSG00000006731	31	7	66	39	32	6	48	11
B4galt1	ENSMUSG00000028413	1799	176	2095	181	1836	106	2087	131
Baalc	ENSMUSG00000022296	20	5	38	10	18	4	29	7
Bax	ENSMUSG00000003873	210	31	278	16	218	17	271	24
BC037034	ENSMUSG00000036948	67	7	102	13	66	9	74	11
Bcl10	ENSMUSG00000028191	379	38	473	26	405	34	468	34
Bcl2	ENSMUSG00000057329	294	11	378	46	307	30	367	43
Bcl2a1b	ENSMUSG00000089929	18	7	68	61	17	5	44	19
Bcl3	ENSMUSG00000053175	60	35	113	24	70	15	98	24
Bcl6b	ENSMUSG00000000317	820	250	1194	317	923	223	1077	280
Bicc1	ENSMUSG00000014329	681	54	1034	279	743	66	924	94
Bin1	ENSMUSG00000024381	163	26	241	35	165	22	215	15
Bmp2k	ENSMUSG00000034663	222	15	328	62	234	20	306	14
Bora	ENSMUSG00000022070	17	4	37	7	18	8	30	4
C1qtnf3	ENSMUSG00000058914	3	2	341	632	3	2	118	157
C1qtnf5	ENSMUSG00000079592	53	6	100	38	61	17	87	20
C1qtnf7	ENSMUSG00000061535	178	26	294	78	174	31	227	32
C3ar1	ENSMUSG00000040552	234	31	502	241	242	41	380	97
Cacnb3	ENSMUSG00000003352	53	8	108	38	53	6	75	17
Calhm5	ENSMUSG00000049872	49	5	97	18	60	11	89	26
Calm2	ENSMUSG00000036438	2322	263	2921	526	2281	193	2735	103
Camk1d	ENSMUSG00000039145	125	28	197	13	146	26	154	32
Camkk1	ENSMUSG00000020785	29	5	54	12	30	5	42	7
Capn6	ENSMUSG00000067276	8	3	42	52	12	8	24	13
Casp12	ENSMUSG00000025887	208	53	318	96	186	49	260	34
Casp3	ENSMUSG00000031628	113	29	191	45	108	17	171	41
Cbfb	ENSMUSG00000031885	509	26	664	130	518	37	561	32
Ccdc88a	ENSMUSG00000032740	355	54	511	63	310	60	391	78
Ccdc88b	ENSMUSG00000047810	26	4	52	17	31	7	43	4
Ccl12	ENSMUSG00000035352	28	12	76	30	20	5	61	36
Ccr2	ENSMUSG00000049103	85	30	408	389	93	25	233	135
Ccr5	ENSMUSG00000079227	186	40	341	137	159	68	244	42
Cd180	ENSMUSG00000021624	33	12	77	31	53	18	71	11
Cd24a	ENSMUSG00000047139	79	19	145	26	90	14	161	75
Cd2ap	ENSMUSG00000061665	690	32	831	97	667	57	750	87
Cd33	ENSMUSG00000004609	150	35	242	42	149	35	189	29
Cd52	ENSMUSG00000000682	61	22	171	113	54	14	95	23
Cd53	ENSMUSG00000040747	129	18	286	154	144	34	221	59
Cd55	ENSMUSG00000026399	345	57	518	88	369	49	468	57
Cd63	ENSMUSG00000025351	1383	97	1809	371	1430	52	1693	169
Cd80	ENSMUSG00000075122	19	7	60	17	24	9	47	22
Cd83	ENSMUSG00000015396	132	30	202	45	132	31	168	13
Cd86	ENSMUSG00000022901	75	19	139	14	88	32	109	23
Cdc25b	ENSMUSG00000027330	92	22	163	19	92	22	150	40
Cdc42se1	ENSMUSG00000046722	468	71	617	41	442	53	554	68
Cdc6	ENSMUSG00000017499	18	6	40	11	16	6	33	8
Cdc7	ENSMUSG00000029283	20	3	41	10	29	11	30	8
Cdca2	ENSMUSG00000048922	13	3	38	8	27	15	36	10
Cdca4	ENSMUSG00000047832	122	18	180	26	120	24	154	24
Cdk14	ENSMUSG00000028926	200	28	273	53	192	35	226	37
Cdkn3	ENSMUSG00000037628	13	6	27	8	12	2	21	5
Cdr2	ENSMUSG00000030878	234	28	362	52	247	34	334	25
Cdr2l	ENSMUSG00000050910	97	21	164	22	96	12	136	9
Cdt1	ENSMUSG00000006585	24	1	51	16	26	8	47	12
Cemip	ENSMUSG00000052353	1	1	29	37	0	0	17	18
Cenpn	ENSMUSG00000031756	14	5	38	13	16	3	27	10
Cep192	ENSMUSG00000024542	181	30	290	42	205	41	226	32
Cercam	ENSMUSG00000039787	51	4	142	102	51	14	90	27
Cggbp1	ENSMUSG00000054604	811	96	987	62	796	114	900	95
Chaf1a	ENSMUSG00000002835	62	28	147	37	69	14	117	37
Chaf1b	ENSMUSG00000022945	12	2	37	18	18	4	36	9
Chst2	ENSMUSG00000033350	33	6	59	8	44	9	45	10
Chsy3	ENSMUSG00000058152	5	1	14	5	5	2	9	3
Cip2a	ENSMUSG00000033031	56	15	113	30	63	19	81	15
Cks1b	ENSMUSG00000028044	53	13	94	14	55	9	83	13

Clca3a1	ENSMUSG00000056025	11	2	33	21	15	8	17	9
Cldn15	ENSMUSG00000001739	49	9	95	9	66	28	67	17
Clec11a	ENSMUSG00000004473	16	2	53	48	14	4	31	14
Clec12a	ENSMUSG00000053063	76	11	211	106	102	28	134	18
Clec4a1	ENSMUSG00000049037	90	14	212	105	96	15	147	36
Clec4a2	ENSMUSG00000030148	46	12	104	63	40	6	79	23
Clec4a3	ENSMUSG00000043832	54	12	126	46	52	9	86	21
Clspn	ENSMUSG00000042489	17	7	42	12	17	3	38	23
Cnn1	ENSMUSG00000001349	37	23	130	75	42	17	53	24
Cnn2	ENSMUSG00000004665	793	93	1039	52	856	80	1002	98
Cnrip1	ENSMUSG00000044629	67	5	110	32	77	10	96	9
Cntln	ENSMUSG00000038070	189	18	279	45	181	18	235	37
Cntrl	ENSMUSG00000057110	277	35	379	18	284	40	270	21
Col11a1	ENSMUSG00000027966	2	3	52	76	2	2	12	11
Col16a1	ENSMUSG00000040690	187	39	603	471	192	60	420	186
Col1a1	ENSMUSG00000001506	1941	334	10243	9888	2115	63	5954	3420
Col1a2	ENSMUSG00000029661	2657	250	11812	10867	2786	231	7551	4300
Col4a5	ENSMUSG00000031274	763	91	1159	304	773	91	971	147
Col5a3	ENSMUSG00000004098	576	178	985	63	666	103	847	222
Col7a1	ENSMUSG00000025650	4	6	26	21	5	4	11	6
Col8a2	ENSMUSG00000056174	9	5	180	298	14	4	86	85
Col9a2	ENSMUSG00000028626	6	1	31	38	7	4	21	11
Comp	ENSMUSG00000031849	39	8	325	426	69	9	179	129
Copb1	ENSMUSG00000030754	983	86	1212	193	1045	57	1076	62
Coro1a	ENSMUSG00000030707	175	29	270	73	171	24	228	26
Coro1b	ENSMUSG00000024835	688	47	858	53	695	47	802	76
Cplx2	ENSMUSG00000025867	149	21	210	31	160	16	218	24
Cpne8	ENSMUSG00000052560	161	16	246	70	161	15	217	22
Creb3l2	ENSMUSG00000038648	1002	88	1355	234	1018	47	1231	121
Crtap	ENSMUSG00000032431	384	57	511	72	379	32	471	67
Csf2ra	ENSMUSG00000059326	56	11	101	28	65	12	82	17
Csrp1	ENSMUSG00000026421	868	80	1281	109	889	121	1075	145
Cstb	ENSMUSG00000005054	171	14	238	43	175	15	218	32
Cthrc1	ENSMUSG00000054196	3	3	221	380	5	2	92	112
Ctsk	ENSMUSG00000028111	95	23	247	204	90	21	156	59
Ctss	ENSMUSG00000038642	458	93	1279	617	459	103	843	274
Cttn	ENSMUSG00000031078	793	94	1056	113	811	56	962	95
Cttnbp2nl	ENSMUSG00000062127	644	157	873	93	653	151	770	81
Cx3cr1	ENSMUSG00000052336	153	23	369	148	170	25	253	86
Cxcl10	ENSMUSG00000034855	12	4	57	37	12	8	27	6
Cybb	ENSMUSG00000015340	327	42	648	221	360	106	406	69
Cysltr1	ENSMUSG00000052821	54	23	122	68	67	22	119	47
Cyth3	ENSMUSG00000018001	939	142	1235	98	1006	115	1159	89
Cyth4	ENSMUSG00000018008	207	45	374	116	216	29	315	54
D1Ertd622e	ENSMUSG00000044768	81	14	134	22	88	9	109	16
Dap	ENSMUSG00000039168	262	38	433	140	293	35	371	55
Dbf4	ENSMUSG00000002297	39	11	81	24	39	8	63	4
Dbnl	ENSMUSG00000020476	472	46	575	30	501	31	573	37
Dck	ENSMUSG00000029366	104	18	188	15	109	4	138	16
Dhx58	ENSMUSG00000017830	37	15	76	24	54	15	63	11
Dkk3	ENSMUSG00000030772	52	16	247	258	56	20	169	78
Dnm1	ENSMUSG00000026825	162	38	244	43	172	24	214	33
Dock11	ENSMUSG00000031093	198	35	312	74	204	30	252	29
Dock7	ENSMUSG00000028556	383	56	520	72	415	36	422	61
Dok3	ENSMUSG00000035711	38	8	70	27	33	8	52	10
Dpep2	ENSMUSG00000053687	21	8	44	15	16	3	33	12
Dpp7	ENSMUSG00000026958	48	11	85	22	56	8	80	17
Dpy19l1	ENSMUSG00000043067	214	23	340	91	229	13	276	30
Dram1	ENSMUSG00000020057	109	11	158	29	104	11	139	11
Dse	ENSMUSG00000039497	182	24	294	74	230	55	247	27
Dsel	ENSMUSG00000038702	152	30	302	163	181	17	233	34
E2f2	ENSMUSG00000018983	15	9	47	2	35	28	45	12
E2f3	ENSMUSG00000016477	259	22	315	27	280	21	306	28
E2f8	ENSMUSG00000046179	31	20	49	20	32	12	50	22
Egr2	ENSMUSG00000037868	32	17	104	54	67	11	86	35
Egr3	ENSMUSG00000033730	55	46	127	33	126	43	192	110
Eif2ak2	ENSMUSG00000024079	457	54	612	78	452	48	564	48

Eif4ebp1	ENSMUSG00000031490	600	42	768	49	633	27	785	117
Elf1	ENSMUSG00000036461	427	66	547	56	479	70	499	46
Eln	ENSMUSG00000029675	512	176	2249	1534	591	55	1327	606
Enpp1	ENSMUSG00000037370	187	9	495	317	181	24	350	178
Epb41l2	ENSMUSG00000019978	1216	183	1533	194	1128	76	1296	65
Epsti1	ENSMUSG00000022014	40	10	90	32	45	8	66	11
Ercc6l	ENSMUSG00000051220	19	8	48	17	17	6	34	13
Esm1	ENSMUSG00000042379	32	4	64	10	34	9	54	17
Etv4	ENSMUSG00000017724	8	5	29	5	8	3	26	18
Etv6	ENSMUSG00000030199	527	36	643	63	544	47	566	37
Evi2a	ENSMUSG00000078771	85	15	172	58	103	15	147	30
Evi2b	ENSMUSG00000093938	37	8	106	37	54	8	77	17
Ezh2	ENSMUSG00000029687	114	28	176	40	98	20	147	19
Fads2	ENSMUSG00000024665	75	13	108	13	83	14	97	12
Fam129a	ENSMUSG00000026483	681	70	925	196	630	59	727	64
Fam167b	ENSMUSG00000050493	7	3	20	8	8	4	17	5
Fam171b	ENSMUSG00000048388	42	7	93	55	48	6	66	20
Fam177a	ENSMUSG00000095595	702	134	843	123	776	69	888	27
Fam83d	ENSMUSG00000027654	6	3	27	7	5	2	17	9
Fam91a1	ENSMUSG00000037119	520	42	707	99	525	28	606	35
Fancd2	ENSMUSG00000034023	13	4	33	8	13	6	28	18
Fap	ENSMUSG00000000392	100	8	185	87	98	34	134	42
Farp1	ENSMUSG00000025555	294	13	417	92	304	53	390	72
Fat1	ENSMUSG00000070047	995	230	1619	361	886	123	1302	330
Fbn2	ENSMUSG00000024598	25	8	112	120	38	10	67	32
Fcer1g	ENSMUSG00000058715	141	25	259	105	135	20	195	28
Fcgr1	ENSMUSG00000015947	58	17	140	69	68	8	103	30
Fermt3	ENSMUSG00000024965	71	21	121	22	77	11	108	24
Fes	ENSMUSG00000053158	170	34	266	19	189	43	230	55
Fgd3	ENSMUSG00000037946	46	14	84	22	51	10	76	20
Fgl2	ENSMUSG00000039899	863	65	1693	575	912	106	1331	391
Fgr	ENSMUSG00000028874	10	5	30	20	9	4	17	11
Fhl1	ENSMUSG00000023092	1773	169	2590	350	1838	138	2464	537
Fibin	ENSMUSG00000074971	181	32	511	420	193	39	346	121
Flt3	ENSMUSG00000042817	2	2	16	13	4	3	8	7
Fmod	ENSMUSG00000041559	30	16	384	621	81	65	169	122
Fmr1	ENSMUSG00000000838	269	36	431	176	300	35	342	37
Fn1	ENSMUSG00000026193	1579	300	7830	6886	1494	199	4711	3367
Foxs1	ENSMUSG00000074676	34	11	64	13	46	5	62	22
Frem1	ENSMUSG00000059049	8	10	51	68	8	4	31	24
Fut11	ENSMUSG00000039357	204	20	286	68	209	19	246	19
Fxyd6	ENSMUSG00000066705	485	36	809	394	515	53	620	96
Fyb	ENSMUSG00000022148	104	44	269	144	111	29	216	57
Fzd1	ENSMUSG00000044674	164	18	259	65	173	23	223	25
G2e3	ENSMUSG00000035293	143	24	208	59	161	20	204	41
Gak	ENSMUSG00000062234	517	39	618	25	529	64	553	30
Garem2	ENSMUSG00000044576	3	3	13	9	3	2	7	4
Gas7	ENSMUSG00000033066	461	108	644	104	455	58	590	51
Gatm	ENSMUSG00000027199	79	9	129	34	80	14	103	20
Gcnt1	ENSMUSG00000038843	85	7	138	43	89	8	117	12
Gcnt4	ENSMUSG00000091387	3	2	26	31	3	1	14	10
Gem	ENSMUSG00000028214	71	22	119	13	95	18	126	28
Gen1	ENSMUSG00000051235	17	8	43	15	30	19	41	16
Gjc1	ENSMUSG00000034520	443	31	538	31	438	34	472	43
Glipr1	ENSMUSG00000056888	19	3	61	50	17	5	34	19
Gm15675	ENSMUSG00000086825	23	4	56	23	34	12	43	15
Gm1966	ENSMUSG00000073902	34	9	90	39	50	9	72	22
Gm2026	ENSMUSG00000078886	29	10	71	41	64	45	50	32
Gm20559	ENSMUSG00000106734	158	25	249	51	169	19	173	37
Gm30873	ENSMUSG00000109341	6	4	25	10	11	7	20	4
Gm36161	ENSMUSG00000114608	18	6	51	47	19	6	34	17
Gm3636	ENSMUSG00000091754	16	5	43	19	24	10	23	5
Gm39214	ENSMUSG00000109754	252	32	351	45	256	25	363	83
Gm42047	ENSMUSG00000110631	86	19	248	70	75	22	169	103
Gm45705	ENSMUSG00000110481	13	3	29	7	14	4	21	3
Gm47761	ENSMUSG00000112478	9	2	26	8	7	2	17	10
Gm49342	ENSMUSG00000021871	79	19	135	20	95	11	98	23

Gm5431	ENSMUSG00000058163	36	7	65	15	44	6	50	7
Gm6377	ENSMUSG00000048621	4	2	18	12	13	16	12	6
Gm8995	ENSMUSG00000063286	770	80	1033	133	749	121	889	142
Gmip	ENSMUSG00000036246	79	23	131	23	79	20	106	20
Gnai2	ENSMUSG00000032562	4185	325	4943	197	4287	161	4768	360
Gnai3	ENSMUSG00000000001	721	11	942	133	676	66	788	53
Gnao1	ENSMUSG00000031748	446	40	615	131	524	114	584	73
Golim4	ENSMUSG00000034109	732	55	1009	144	790	46	896	57
Gpc6	ENSMUSG00000058571	240	29	347	74	228	14	308	59
Gpnmb	ENSMUSG00000029816	23	2	58	33	25	12	38	17
Gpr176	ENSMUSG00000040133	6	3	37	26	7	1	23	18
Gpr34	ENSMUSG00000040229	63	25	126	68	77	29	101	26
Gpr39	ENSMUSG00000026343	4	2	28	20	6	3	21	14
Gpr65	ENSMUSG00000021886	44	9	108	76	42	12	70	21
Gpr68	ENSMUSG00000047415	3	3	18	4	8	2	10	7
Gpx8	ENSMUSG00000021760	491	35	682	134	493	63	644	76
Gria3	ENSMUSG00000001986	27	6	74	55	40	11	71	37
Gsap	ENSMUSG00000039934	66	8	121	24	74	13	95	10
Gxylt2	ENSMUSG00000030074	171	37	519	541	157	23	308	184
Hacd4	ENSMUSG00000028497	224	6	334	92	257	33	288	32
Has2	ENSMUSG00000022367	27	6	50	13	28	8	42	6
Haus8	ENSMUSG00000035439	242	44	381	64	261	50	360	59
Havcr2	ENSMUSG00000020399	17	6	42	21	12	4	23	11
Hbb-bt	ENSMUSG00000073940	3565	1643	6280	1861	4671	1644	8009	3238
Hck	ENSMUSG00000003283	35	10	88	47	39	12	58	8
Hdac1	ENSMUSG00000028800	455	34	557	48	447	37	529	38
Hexa	ENSMUSG00000025232	1121	111	1417	213	1139	67	1382	81
Hexb	ENSMUSG00000021665	487	58	816	323	492	50	666	110
Hjurp	ENSMUSG00000044783	577	122	775	43	589	90	674	87
Hmgb2	ENSMUSG00000054717	115	21	211	30	135	37	202	60
Hmgn3	ENSMUSG00000066456	78	20	130	43	71	15	108	14
Hpgd	ENSMUSG00000031613	182	26	300	44	240	31	258	32
Hpgds	ENSMUSG00000029919	67	16	152	74	91	33	92	21
Hspg2	ENSMUSG00000028763	12506	1017	15279	1628	12610	707	14990	1190
Iffo2	ENSMUSG00000041025	210	56	296	34	208	41	260	55
Ifi203	ENSMUSG00000039997	891	161	1230	255	880	117	940	84
Ifi209	ENSMUSG00000043263	52	15	126	69	50	17	92	45
Ifi211	ENSMUSG00000026536	185	36	351	104	178	18	256	21
Ifi30	ENSMUSG00000031838	101	27	193	76	87	20	127	31
Ifih1	ENSMUSG00000026896	299	32	446	46	329	56	378	53
Ifit1	ENSMUSG00000034459	187	43	329	102	161	24	234	21
Ifit2	ENSMUSG00000045932	547	92	934	140	539	58	669	52
Ifit3	ENSMUSG00000074896	336	93	626	210	311	45	430	29
Ifit3b	ENSMUSG00000062488	122	30	208	57	123	22	150	9
Igf1	ENSMUSG00000020053	384	78	1064	595	422	57	770	126
Igf2bp2	ENSMUSG00000033581	72	26	122	41	102	14	135	38
Igfbp2	ENSMUSG00000039323	1	1	24	20	0	1	4	4
Igfbp5	ENSMUSG00000026185	2981	305	5165	1633	3493	830	4402	921
Ighm	ENSMUSG00000076617	206	22	302	49	234	29	292	34
Igsf10	ENSMUSG00000036334	145	33	315	123	162	38	247	69
Ikzf1	ENSMUSG00000018654	64	21	119	32	75	10	86	10
Il13ra1	ENSMUSG00000017057	1206	209	1526	234	1225	65	1443	213
Il18rap	ENSMUSG00000026068	5	4	17	7	3	2	6	4
Il1b	ENSMUSG00000027398	20	8	71	68	17	7	29	8
Il21r	ENSMUSG00000030745	15	3	46	25	17	2	35	6
Ildr2	ENSMUSG00000040612	25	9	63	21	39	23	51	20
Irf5	ENSMUSG00000029771	73	13	148	22	78	12	119	35
Irf8	ENSMUSG00000041515	118	13	229	76	128	21	193	26
Isg15	ENSMUSG00000035692	59	12	120	28	67	16	93	12
Isg20	ENSMUSG00000039236	86	30	130	16	102	27	144	32
Islr	ENSMUSG00000037206	560	95	865	285	608	50	774	128
Itga4	ENSMUSG00000027009	88	24	170	60	100	20	120	28
Itgav	ENSMUSG00000027087	584	81	920	293	645	110	863	204
Itgax	ENSMUSG00000030789	14	13	49	40	11	3	41	38
Itgb1	ENSMUSG00000025809	10648	748	12926	1642	10469	431	12265	1179
Itgb2	ENSMUSG00000000290	175	40	280	60	156	29	225	46
Itgb3	ENSMUSG00000020689	55	19	135	26	74	35	101	24

Itgb8	ENSMUSG00000025321	44	17	94	45	58	14	57	7
Itgbl1	ENSMUSG00000032925	238	42	795	674	281	46	513	150
Itih2	ENSMUSG00000037254	10	2	36	22	10	1	20	5
Itm2a	ENSMUSG00000031239	204	26	483	405	214	32	305	66
Kcne1	ENSMUSG00000039639	91	46	147	42	92	14	127	27
Kctd10	ENSMUSG00000001098	1166	95	1425	67	1193	154	1325	119
Kctd12	ENSMUSG00000098557	889	110	1244	276	915	43	1073	34
Kctd12b	ENSMUSG00000041633	620	130	959	330	830	137	891	240
Kctd15	ENSMUSG00000030499	38	5	65	13	39	8	54	12
Kdelr3	ENSMUSG00000010830	122	17	231	83	128	25	188	48
Kif18a	ENSMUSG00000027115	33	7	55	18	33	3	53	12
Kif20b	ENSMUSG00000024795	44	17	104	56	35	19	80	25
Kirrel	ENSMUSG00000041734	354	32	515	106	351	32	432	45
Klhl29	ENSMUSG00000020627	12	4	36	26	16	6	21	5
Krt18	ENSMUSG00000023043	11	4	41	28	17	8	20	7
Lacc1	ENSMUSG00000044350	110	10	170	40	111	18	129	16
Lair1	ENSMUSG00000055541	93	25	193	59	88	18	152	37
Lbp	ENSMUSG00000016024	114	14	211	51	141	27	174	37
Ldlrad4	ENSMUSG00000024544	207	23	293	57	198	24	230	26
Lhfp	ENSMUSG00000048332	883	63	1348	372	958	40	1113	122
Lilrb4a	ENSMUSG00000112148	184	42	406	123	195	44	266	91
Lix1l	ENSMUSG00000049288	862	45	1020	63	903	22	948	26
Lmna	ENSMUSG00000028063	1287	73	1552	83	1330	102	1508	161
Lmnb1	ENSMUSG00000024590	122	21	210	37	129	15	185	47
Lox	ENSMUSG00000024529	89	9	1050	1321	94	21	476	436
Loxl3	ENSMUSG00000000693	104	36	311	233	87	12	196	70
Lpcat2	ENSMUSG00000033192	36	10	94	53	31	6	64	11
Lpp	ENSMUSG00000033306	1398	218	1794	95	1378	115	1689	226
Lpxn	ENSMUSG00000024696	18	5	55	29	22	7	34	9
Lrmp	ENSMUSG00000030263	36	7	68	14	45	10	53	5
Lrrc32	ENSMUSG00000090958	328	91	455	36	361	64	432	52
Lrrc59	ENSMUSG00000020869	766	27	883	28	772	44	888	71
Lsp1	ENSMUSG00000018819	614	136	984	350	600	112	808	176
Lst1	ENSMUSG00000073412	16	5	43	19	20	5	34	7
Ltbp2	ENSMUSG00000002020	94	45	964	1120	126	62	578	514
Ltbp3	ENSMUSG00000024940	530	28	924	412	618	50	772	112
Lum	ENSMUSG00000036446	1447	238	3824	2686	1553	192	2795	564
Lxn	ENSMUSG00000047557	65	9	139	25	70	19	104	26
Lyl1	ENSMUSG00000034041	75	11	111	12	85	3	91	4
Maf	ENSMUSG00000055435	389	56	552	118	424	20	460	80
Maff	ENSMUSG00000042622	133	18	220	64	128	36	187	43
Malt1	ENSMUSG00000032688	196	29	270	27	193	17	212	27
Mapre1	ENSMUSG00000027479	1639	151	1876	92	1651	91	1859	64
Marcks	ENSMUSG00000069662	819	115	1485	784	999	154	1070	121
Mastl	ENSMUSG00000026779	21	19	58	17	22	7	58	28
Matn2	ENSMUSG00000022324	417	57	667	192	429	35	559	39
Mcm5	ENSMUSG00000005410	84	12	162	45	83	19	169	35
Mcm6	ENSMUSG00000026355	230	32	397	77	216	18	363	70
Mcub	ENSMUSG00000027994	41	14	77	15	37	4	50	10
Mdk	ENSMUSG00000027239	26	4	95	130	24	6	43	34
Meg3	ENSMUSG00000021268	123	46	231	107	121	17	140	38
Megf10	ENSMUSG00000024593	19	6	49	31	30	3	40	15
Mex3c	ENSMUSG00000037253	486	50	607	52	503	15	557	37
Mfap2	ENSMUSG00000060572	20	2	69	61	23	4	41	14
Mfap3	ENSMUSG00000020522	454	25	541	33	431	20	503	22
Mgam	ENSMUSG00000068587	9	4	32	24	15	4	30	21
Mgat2	ENSMUSG00000043998	423	27	547	64	443	34	503	31
Mgp	ENSMUSG00000030218	1671	257	3299	1832	1627	149	2424	267
Milr1	ENSMUSG00000040528	16	6	43	18	16	4	35	10
Mis18a	ENSMUSG00000022978	46	8	79	17	61	14	68	8
Mkrn1	ENSMUSG00000029922	598	102	779	95	681	119	829	128
Mmp14	ENSMUSG00000000957	291	39	862	471	332	50	582	198
Mmp16	ENSMUSG00000028226	14	6	34	13	18	6	22	9
Mns1	ENSMUSG00000032221	9	2	49	8	14	7	28	16
Mob1a	ENSMUSG00000043131	1089	121	1399	155	1053	73	1248	99
Mpeg1	ENSMUSG00000046805	387	93	1323	966	395	77	809	361
Mpzl1	ENSMUSG00000026566	252	38	353	54	258	43	287	41

Mrc2	ENSMUSG00000020695	462	41	913	394	473	31	716	159
Ms4a14	ENSMUSG00000099398	18	2	74	52	16	6	34	17
Ms4a4b	ENSMUSG00000056290	4	2	18	15	8	4	9	4
Ms4a4c	ENSMUSG00000024675	8	4	38	33	10	3	16	8
Ms4a6d	ENSMUSG00000024679	61	21	136	54	54	15	109	45
Ms4a7	ENSMUSG00000024672	65	11	296	303	51	5	166	95
Mtfr2	ENSMUSG00000019992	4	2	15	5	3	1	10	2
Mthfd2	ENSMUSG00000005667	41	11	81	14	49	16	81	18
Mtmr11	ENSMUSG00000045934	69	26	114	28	68	30	107	32
Mtpn	ENSMUSG00000029840	1161	76	1505	277	1222	89	1380	115
Mx1	ENSMUSG00000000386	20	10	55	32	17	6	22	11
Mxra8	ENSMUSG00000029070	431	61	782	329	511	145	714	119
Myc	ENSMUSG00000022346	48	8	98	19	58	7	95	39
Myef2	ENSMUSG00000027201	185	24	262	27	174	6	207	19
Myh7	ENSMUSG00000053093	1672	510	20844	27743	2490	1006	11438	8811
Myh9	ENSMUSG00000022443	4467	917	6229	813	4465	679	5733	609
Myl9	ENSMUSG00000067818	507	113	744	105	504	152	536	41
Myo1e	ENSMUSG00000032220	382	52	541	82	377	40	487	59
Myo1g	ENSMUSG00000020437	50	9	89	21	55	12	90	33
Myo5a	ENSMUSG00000034593	359	55	615	165	371	102	518	152
Naalad2	ENSMUSG00000043943	352	43	575	192	346	54	447	64
Nab2	ENSMUSG00000025402	178	29	277	77	177	31	248	30
Nav1	ENSMUSG00000009418	1498	290	1893	171	1457	90	1737	179
Ncapd2	ENSMUSG00000038252	298	45	437	112	363	44	394	55
Nedd9	ENSMUSG00000021365	728	336	1059	86	794	203	936	202
Neil3	ENSMUSG00000039396	10	3	35	11	13	11	23	8
Neurl3	ENSMUSG00000047180	159	33	303	74	176	25	246	35
Nfam1	ENSMUSG00000058099	110	22	202	30	118	21	160	20
Nfkbie	ENSMUSG00000023947	37	6	81	15	38	5	56	8
Nfkbiz	ENSMUSG00000035356	92	25	164	59	103	9	151	32
Nhsl2	ENSMUSG00000079481	276	31	399	49	272	39	339	24
Nkd1	ENSMUSG00000031661	22	8	42	10	25	6	30	7
Nlgn2	ENSMUSG00000051790	167	30	260	63	167	33	228	39
Nmrk2	ENSMUSG00000004939	73	9	158	71	68	15	132	76
Nmt2	ENSMUSG00000026643	286	28	378	59	277	25	339	48
Nnmt	ENSMUSG00000032271	42	5	88	24	47	10	60	8
Nox4	ENSMUSG00000030562	31	5	162	173	29	9	64	30
Npdc1	ENSMUSG00000015094	238	45	344	63	227	27	280	28
Npl	ENSMUSG00000042684	26	8	47	8	28	8	51	12
Npnt	ENSMUSG00000040998	22	7	57	25	31	14	28	9
Nptxr	ENSMUSG00000022421	69	12	105	10	64	14	94	14
Nrep	ENSMUSG00000042834	535	52	997	503	559	60	711	91
Nrros	ENSMUSG00000052384	157	25	231	32	162	27	220	28
Nts	ENSMUSG00000019890	21	8	42	6	25	4	33	13
Nucb2	ENSMUSG00000030659	137	19	239	55	144	20	174	24
Nupr1	ENSMUSG00000030717	60	10	196	120	74	8	130	33
Nusap1	ENSMUSG00000027306	48	17	132	40	45	9	116	36
Nxpe4	ENSMUSG00000044229	315	33	430	45	320	35	379	48
Nxpe5	ENSMUSG00000047592	11	6	35	8	9	4	28	15
Oas1a	ENSMUSG00000052776	82	18	149	24	88	14	121	8
Oas3	ENSMUSG00000032661	6	1	34	18	9	3	18	9
Oasl1	ENSMUSG00000041827	32	9	56	13	38	7	48	5
Oasl2	ENSMUSG00000029561	269	62	498	109	332	69	402	43
Olfr558	ENSMUSG00000070423	167	26	236	25	154	13	204	33
Olfr56	ENSMUSG00000040328	6	3	19	7	8	2	10	5
Omd	ENSMUSG00000048368	16	4	58	62	32	17	29	10
Otulinl	ENSMUSG00000056069	53	9	133	67	61	15	119	66
P2rx7	ENSMUSG00000029468	140	30	223	46	141	24	188	49
P3h1	ENSMUSG00000028641	217	25	313	43	242	18	308	55
P4ha3	ENSMUSG00000051048	2	2	23	32	1	1	11	10
Pak1	ENSMUSG00000030774	34	10	89	41	49	20	57	18
Panx1	ENSMUSG00000031934	35	11	98	49	42	21	76	30
Parp9	ENSMUSG00000022906	467	40	651	71	484	24	548	44
Parpbp	ENSMUSG00000035365	8	4	24	9	7	2	18	11
Pcdh17	ENSMUSG00000035566	299	64	458	76	316	37	426	89
Pcsk5	ENSMUSG00000024713	67	10	129	24	84	4	119	16
Pdgfrl	ENSMUSG00000031595	48	13	182	194	44	4	108	62

Pdk3	ENSMUSG00000035232	44	14	83	17	53	21	69	7
Pdlim2	ENSMUSG00000022090	106	9	175	59	121	17	153	29
Pdlim3	ENSMUSG00000031636	187	22	301	87	190	20	249	48
Pdpn	ENSMUSG00000028583	91	16	188	37	105	17	159	30
Pgm2	ENSMUSG00000029171	116	24	174	30	127	15	159	19
Phf11b	ENSMUSG00000091649	41	10	91	36	39	9	65	19
Phldb2	ENSMUSG00000033149	633	66	824	141	626	28	795	54
Pi15	ENSMUSG00000067780	74	15	180	121	89	17	105	28
Piezo1	ENSMUSG00000014444	764	311	1009	184	762	82	868	67
Piezo2	ENSMUSG00000041482	24	11	103	100	27	10	60	26
Pif1	ENSMUSG00000041064	7	7	20	9	7	4	13	5
Pik3ap1	ENSMUSG00000025017	55	12	132	58	64	10	102	14
Pik3c2a	ENSMUSG00000030660	644	90	821	49	571	41	614	49
Pik3cd	ENSMUSG00000039936	104	25	184	49	123	16	148	46
Pik3cg	ENSMUSG00000020573	96	11	139	24	110	14	125	19
Pik3r5	ENSMUSG00000020901	24	7	70	31	28	9	52	21
Pkd2	ENSMUSG00000034462	934	68	1341	308	928	43	1049	85
Pkhd1l1	ENSMUSG00000038725	113	45	211	84	195	90	172	61
Pkn3	ENSMUSG00000026785	143	28	220	23	138	15	195	21
Plac8	ENSMUSG00000029322	10	2	54	49	9	5	21	10
Plau	ENSMUSG00000021822	120	25	172	15	122	29	160	33
Plaur	ENSMUSG00000046223	36	7	71	21	37	23	64	15
Pld4	ENSMUSG00000052160	158	21	294	102	174	44	228	58
Plekha4	ENSMUSG00000040428	34	16	87	40	51	13	73	38
Plk1	ENSMUSG00000030867	19	10	48	16	17	7	40	14
Plk4	ENSMUSG00000025758	70	9	110	26	68	16	112	44
Plod3	ENSMUSG00000004846	475	118	658	87	472	51	605	62
Plp2	ENSMUSG00000031146	570	31	793	128	592	14	724	133
Plpp1	ENSMUSG00000021759	1000	50	1160	56	1013	21	1087	43
Plxdc2	ENSMUSG00000026748	599	71	840	221	565	51	696	96
Pou2f2	ENSMUSG00000008496	37	5	109	53	61	19	105	35
Ppfia1	ENSMUSG00000037519	773	148	999	166	867	163	931	191
Ppfibp1	ENSMUSG00000016487	1707	135	2004	276	1903	142	1868	195
Ppib	ENSMUSG00000032383	1085	51	1440	242	1046	78	1281	107
Ppp1r15b	ENSMUSG00000046062	750	74	889	76	775	49	880	44
Ppp1r18	ENSMUSG00000034595	415	70	651	61	435	37	557	57
Pqlc3	ENSMUSG00000045679	95	9	171	87	101	15	126	18
Prelp	ENSMUSG00000041577	1273	232	1575	178	1325	81	1511	142
Prex1	ENSMUSG00000039621	261	20	427	60	278	27	341	38
Prim1	ENSMUSG00000025395	42	9	75	26	45	8	65	14
Prkcd	ENSMUSG00000021948	246	36	398	115	245	27	325	36
Prr5l	ENSMUSG00000032841	24	3	49	21	25	8	44	16
Prrt4	ENSMUSG00000079654	41	13	75	15	44	3	68	12
Prss23	ENSMUSG00000039405	314	52	488	126	314	35	361	78
Pstpip1	ENSMUSG00000032322	21	5	54	31	21	3	37	15
Ptafr	ENSMUSG00000056529	107	31	167	16	115	20	154	29
Ptbp1	ENSMUSG00000006498	741	116	1045	159	740	51	886	127
Pthlh	ENSMUSG00000048776	10	3	24	10	10	4	20	5
Ptk2b	ENSMUSG00000059456	161	11	248	52	179	24	246	35
Ptk7	ENSMUSG00000023972	64	11	121	51	79	10	101	16
Ptms	ENSMUSG00000030122	1096	180	1556	255	1175	69	1354	123
Ptn	ENSMUSG00000029838	35	10	527	950	47	13	109	85
Ptpn1	ENSMUSG00000027540	383	52	516	71	406	46	484	90
Ptpn12	ENSMUSG00000028771	720	48	925	82	719	64	779	35
Ptpn18	ENSMUSG00000026126	35	12	64	9	54	15	62	12
Ptpn6	ENSMUSG00000004266	114	27	202	70	107	24	164	25
Ptprc	ENSMUSG00000026395	246	49	533	285	234	45	382	129
Ptpre	ENSMUSG00000041836	163	23	260	20	183	21	214	24
Ptprf	ENSMUSG00000033295	111	21	196	66	107	19	177	20
Rab23	ENSMUSG00000004768	193	52	284	32	226	32	252	58
Rab32	ENSMUSG00000019832	40	12	85	42	46	3	70	12
Rab3il1	ENSMUSG00000024663	211	18	296	51	216	26	283	32
Rab5c	ENSMUSG00000019173	838	81	1059	21	918	29	1032	128
Rab8b	ENSMUSG00000036943	406	32	589	93	439	43	531	48
Rad50	ENSMUSG00000020380	310	33	381	31	339	21	385	26
Rai14	ENSMUSG00000022246	173	18	300	114	173	15	241	57
Rap1b	ENSMUSG00000052681	1590	124	2099	385	1642	155	1892	156

Rasa4	ENSMUSG00000004952	143	26	261	113	138	14	162	33
Rbbp8	ENSMUSG00000041238	177	8	233	32	161	22	196	17
Rbm3	ENSMUSG00000031167	1065	243	1408	328	1097	137	1294	160
Rbp1	ENSMUSG00000046402	203	16	395	267	185	21	311	56
Reps2	ENSMUSG00000040855	67	11	117	35	105	41	101	23
Rflnb	ENSMUSG00000020846	1333	290	1883	230	1446	172	1704	352
Rfx7	ENSMUSG00000037674	332	31	421	33	374	29	376	17
Rgs10	ENSMUSG00000030844	114	9	181	62	108	10	146	24
Rhoa	ENSMUSG00000007815	4789	93	5216	118	4862	194	5112	92
Rhod	ENSMUSG00000041845	59	10	92	15	68	16	66	10
Rhoj	ENSMUSG00000046768	564	95	704	75	573	74	656	48
Rhou	ENSMUSG00000039960	110	28	173	59	135	9	173	41
Rnase6	ENSMUSG00000021880	9	5	29	15	13	7	14	2
Robo1	ENSMUSG00000022883	56	11	110	44	68	14	97	13
Rpl10-ps3	ENSMUSG00000058443	1037	413	1554	348	1563	299	1415	424
Rpl3	ENSMUSG00000060036	3377	296	4471	607	3263	335	4320	344
Rps6ka1	ENSMUSG00000003644	155	10	219	28	140	11	177	26
Rtn4	ENSMUSG00000020458	1597	115	2738	963	1691	68	2291	384
Rtp4	ENSMUSG00000033355	158	33	260	39	177	33	212	32
Runx1	ENSMUSG00000022952	63	29	176	93	54	10	115	46
S1pr2	ENSMUSG00000043895	122	33	225	28	161	13	210	36
Samd14	ENSMUSG00000047181	37	7	72	30	41	5	56	15
Samsn1	ENSMUSG00000022876	14	3	34	12	17	4	26	8
Sat1	ENSMUSG00000025283	655	35	1037	357	699	101	854	124
Sbno2	ENSMUSG00000035673	285	44	452	89	290	31	409	72
Scara3	ENSMUSG00000034463	56	13	114	45	58	2	87	26
Scd2	ENSMUSG00000025203	637	102	838	65	763	157	803	46
Scn1b	ENSMUSG00000019194	235	13	365	140	268	19	330	60
Scpep1	ENSMUSG00000000278	287	48	480	174	308	44	404	65
Scrn1	ENSMUSG00000019124	212	35	302	33	202	45	226	61
Scube3	ENSMUSG00000038677	5	4	20	12	8	3	16	11
Sdc1	ENSMUSG00000020592	116	15	220	98	124	18	164	39
Sdcbp	ENSMUSG00000028249	2167	214	2679	438	2118	92	2530	189
Sdk1	ENSMUSG00000039683	24	3	57	13	40	21	57	22
Sec16b	ENSMUSG00000026589	57	7	120	43	63	10	73	13
Sele	ENSMUSG00000026582	35	8	70	23	43	4	63	17
Selplg	ENSMUSG00000048163	63	15	127	38	67	10	99	16
Sema6d	ENSMUSG00000027200	1026	136	1377	210	1090	137	1238	97
Serp1	ENSMUSG00000027808	586	74	881	294	591	79	738	92
Serpina3g	ENSMUSG00000041481	9	5	29	16	12	5	21	10
Serpina3i	ENSMUSG00000079014	3	2	17	15	2	2	10	8
Serpina3n	ENSMUSG00000021091	176	100	698	430	134	41	495	374
Serpinb1a	ENSMUSG00000044734	77	13	216	200	93	25	137	59
Serpine1	ENSMUSG00000037411	794	250	1688	848	832	284	1309	495
Serpine2	ENSMUSG00000026249	478	38	814	242	497	57	623	60
Serping1	ENSMUSG00000023224	1822	273	2473	457	1827	186	2279	296
Sertad4	ENSMUSG00000016262	105	17	221	144	112	18	169	32
Sfrp1	ENSMUSG00000031548	345	30	981	861	365	40	683	270
Sfrp2	ENSMUSG00000027996	43	4	313	445	47	8	154	132
Sfxn3	ENSMUSG00000025212	216	19	310	29	224	5	272	31
Sgce	ENSMUSG00000004631	294	28	387	53	287	38	342	18
Sh3bgrl	ENSMUSG00000031246	918	89	1283	246	887	122	1015	78
Shc2	ENSMUSG00000020312	50	9	88	30	52	6	71	5
Shcbp1	ENSMUSG00000022322	19	11	66	49	19	6	42	16
Shisa4	ENSMUSG00000041889	45	8	85	28	49	10	83	18
Shisa5	ENSMUSG00000025647	705	100	1028	56	749	29	862	118
Shtn1	ENSMUSG00000041362	111	11	174	21	133	10	158	33
Siglece	ENSMUSG00000030474	31	11	61	22	29	7	49	8
Sirpa	ENSMUSG00000037902	816	78	1138	211	857	29	986	159
Ska1	ENSMUSG00000036223	3	3	16	11	2	2	11	8
Ska3	ENSMUSG00000021965	7	5	20	4	7	1	19	6
Skil	ENSMUSG00000027660	581	92	881	203	556	78	727	130
Skp2	ENSMUSG00000054115	26	2	53	9	32	9	43	12
Sla	ENSMUSG00000022372	69	14	123	46	60	7	74	15
Slamf7	ENSMUSG00000038179	16	7	59	44	20	9	31	22
Slbp	ENSMUSG00000004642	270	27	364	58	264	33	311	22
Slc15a3	ENSMUSG00000024737	89	19	150	23	90	18	130	28

Slc20a1	ENSMUSG00000027397	373	83	570	141	383	75	430	73
Slc25a24	ENSMUSG00000040322	184	16	278	63	197	22	246	35
Slc25a45	ENSMUSG00000024818	85	11	134	30	87	26	111	13
Slc39a6	ENSMUSG00000024270	135	18	213	64	147	13	187	32
Slc7a8	ENSMUSG00000022180	85	8	120	20	95	6	114	11
Slc9a1	ENSMUSG00000028854	394	28	509	56	454	36	435	54
Slc9a9	ENSMUSG00000031129	122	18	169	14	122	7	161	18
Slco2a1	ENSMUSG00000032548	79	12	158	76	109	32	151	52
Slfn1	ENSMUSG00000078763	6	3	30	22	7	4	14	5
Slfn4	ENSMUSG00000000204	4	3	37	45	3	3	7	6
Slfn8	ENSMUSG00000035208	92	15	162	48	81	9	133	41
Smc4	ENSMUSG00000034349	507	95	871	221	482	64	678	208
Smg1	ENSMUSG00000030655	1722	260	2176	77	1762	176	1909	93
Snhg18	ENSMUSG00000096956	105	9	186	61	111	26	157	23
Snx18	ENSMUSG00000042364	553	47	696	92	562	61	610	52
Snx20	ENSMUSG00000031662	19	11	47	15	23	2	34	9
Snx5	ENSMUSG00000027423	1439	125	1707	156	1541	86	1646	122
Soat1	ENSMUSG00000026600	139	29	253	90	127	33	216	79
Sp3	ENSMUSG00000027109	1028	55	1219	141	1088	81	1139	66
Spi1	ENSMUSG00000002111	75	11	144	49	95	22	131	8
Spidr	ENSMUSG00000041974	67	16	111	18	68	12	82	12
Spin4	ENSMUSG00000071722	30	7	51	5	34	7	38	7
Spn	ENSMUSG00000051457	31	12	75	26	34	10	54	21
Spp1	ENSMUSG00000029304	7	3	219	250	5	2	107	189
Spred1	ENSMUSG00000027351	771	71	991	172	810	79	960	76
Sprr2a2	ENSMUSG00000068893	5	4	45	45	7	3	19	6
Spty2d1	ENSMUSG00000049516	303	36	432	55	340	21	357	39
Sqle	ENSMUSG00000022351	22	13	44	9	32	12	32	12
Srgap1	ENSMUSG00000020121	122	17	179	21	119	24	155	30
Ssh1	ENSMUSG00000042121	389	56	482	27	429	54	403	41
St14	ENSMUSG00000031995	4	1	21	10	6	4	18	19
Star	ENSMUSG00000031574	18	11	140	152	32	27	93	89
Stk26	ENSMUSG00000031112	14	4	39	26	33	20	26	15
Stk32c	ENSMUSG00000015981	20	5	41	5	22	5	29	4
Stt3a	ENSMUSG00000032116	850	44	1211	182	933	75	948	77
Stxbp2	ENSMUSG00000004626	39	12	87	17	33	5	50	10
Sulf2	ENSMUSG00000006800	1067	166	1425	253	1164	107	1386	119
Susd2	ENSMUSG00000006342	39	13	86	24	45	10	49	10
Susd5	ENSMUSG00000086596	20	9	62	31	26	9	31	13
Synpo	ENSMUSG00000043079	2231	119	2747	352	2295	170	2766	301
Syt12	ENSMUSG00000049303	54	21	128	28	50	24	89	45
Tagln	ENSMUSG00000032085	483	160	931	216	458	122	542	128
Taok3	ENSMUSG00000061288	238	28	320	48	253	36	307	26
Tbpl1	ENSMUSG00000071359	277	18	347	9	269	16	294	34
Tbx15	ENSMUSG00000027868	14	5	36	21	18	11	36	10
Tbxas1	ENSMUSG00000029925	62	14	104	27	75	23	89	12
Tcaf1	ENSMUSG00000036667	298	59	423	88	292	16	368	39
Tceal9	ENSMUSG00000042712	276	42	454	184	290	28	356	21
Tcirg1	ENSMUSG00000001750	143	21	252	50	181	41	211	53
Tent5a	ENSMUSG00000032265	352	77	521	110	348	40	469	106
Tent5c	ENSMUSG00000044468	180	62	415	151	316	121	530	280
Tep1	ENSMUSG00000006281	281	10	425	52	275	19	343	44
Tgfb1i1	ENSMUSG00000030782	331	73	487	33	326	41	417	38
Tgfb3	ENSMUSG00000021253	358	51	819	586	360	30	577	163
Tgfbr1	ENSMUSG00000007613	473	61	783	295	536	62	608	144
Tgfbr2	ENSMUSG00000032440	1323	221	1904	229	1360	84	1637	48
Tgif2	ENSMUSG00000062175	15	4	33	9	22	11	27	10
Themis2	ENSMUSG00000037731	94	14	167	25	103	12	120	24
Thoc1	ENSMUSG00000024287	393	27	507	81	425	40	457	36
Tifab	ENSMUSG00000049625	51	11	93	26	49	6	72	19
Tlr1	ENSMUSG00000044827	25	12	79	59	23	4	49	22
Tlr2	ENSMUSG00000027995	103	10	196	69	107	15	152	23
Tlr6	ENSMUSG00000051498	15	4	36	10	18	9	18	10
Tlr7	ENSMUSG00000044583	100	10	218	51	119	44	182	57
Tm6sf1	ENSMUSG00000038623	189	36	283	38	193	35	228	22
Tmem119	ENSMUSG00000054675	65	21	180	126	65	6	118	38
Tmem132a	ENSMUSG00000024736	173	25	238	29	190	15	225	33

Tmem165	ENSMUSG00000029234	367	42	504	119	398	39	467	29
Tmem184c	ENSMUSG00000031617	277	31	380	62	313	36	352	23
Tmem198b	ENSMUSG00000047090	101	18	156	32	99	12	129	16
Tmem273	ENSMUSG00000041707	12	5	33	15	17	6	24	6
Tmem45a	ENSMUSG00000022754	60	25	158	66	68	11	112	16
Tmem51	ENSMUSG00000040616	54	4	82	7	68	8	69	12
Tnfrsf1b	ENSMUSG00000028599	240	51	378	73	263	41	340	52
Tnnt3	ENSMUSG00000061723	6	2	29	39	5	3	17	17
Tns3	ENSMUSG00000020422	616	54	885	194	633	54	721	71
Tor3a	ENSMUSG00000060519	274	45	395	49	295	22	340	25
Tram2	ENSMUSG00000041779	231	36	333	42	225	59	272	55
Trf	ENSMUSG00000032554	860	217	1129	103	862	83	1027	78
Trim30a	ENSMUSG00000030921	325	27	481	93	321	43	427	50
Trip13	ENSMUSG00000021569	9	5	30	12	10	2	26	8
Trp53i11	ENSMUSG00000068735	646	173	926	105	688	75	760	211
Tshz3	ENSMUSG00000021217	83	14	129	7	86	15	111	16
Tsku	ENSMUSG00000049580	67	12	104	26	53	7	91	27
Tspan18	ENSMUSG00000027217	290	49	403	41	317	28	366	89
Tspo	ENSMUSG00000041736	297	21	484	201	308	28	397	63
Ttyh3	ENSMUSG00000036565	273	38	397	63	320	15	336	32
Tubb2b	ENSMUSG00000045136	49	15	127	28	62	34	105	43
Txndc5	ENSMUSG00000038991	889	51	1133	196	889	39	1068	85
Ube2l6	ENSMUSG00000027078	317	72	521	98	342	63	524	145
Uchl1	ENSMUSG00000029223	115	13	232	135	124	16	194	68
Ugdh	ENSMUSG00000029201	332	33	438	36	370	92	419	88
Upp1	ENSMUSG00000020407	78	13	127	37	87	15	115	29
Usp18	ENSMUSG00000030107	82	19	133	27	74	13	106	20
Usp6nl	ENSMUSG00000039046	217	31	297	27	260	46	269	17
Vash1	ENSMUSG00000021256	340	36	487	83	322	15	460	115
Vasn	ENSMUSG00000039646	131	17	195	17	135	19	160	13
Vasp	ENSMUSG00000030403	602	62	764	80	624	92	708	80
Vgll3	ENSMUSG00000091243	127	49	249	84	167	31	215	72
Vsir	ENSMUSG00000020101	544	130	797	85	643	20	694	88
Was	ENSMUSG00000031165	34	9	71	17	40	6	48	12
Wipf1	ENSMUSG00000075284	572	54	783	47	562	70	635	43
Wisp1	ENSMUSG00000005124	27	8	174	241	30	12	82	66
Wnt9b	ENSMUSG00000018486	9	5	34	16	8	4	21	10
Wsb1	ENSMUSG00000017677	413	65	588	117	430	59	499	86
Wwtr1	ENSMUSG00000027803	1788	70	2201	189	1899	86	2118	63
Xaf1	ENSMUSG00000040483	194	44	280	32	185	32	215	10
Xcr1	ENSMUSG00000060509	4	4	16	13	3	2	5	4
Yes1	ENSMUSG00000014932	493	48	631	79	517	38	549	53
Yipf5	ENSMUSG00000024487	306	19	418	92	321	20	373	55
Ywhaq	ENSMUSG00000076432	1324	72	1556	97	1256	62	1410	136
Zbp1	ENSMUSG00000027514	37	19	92	33	37	4	63	13
Zdhhc20	ENSMUSG00000021969	297	35	512	168	404	125	438	107
Zfas1	ENSMUSG00000074578	115	9	200	96	116	17	147	30
Zfp185	ENSMUSG00000031351	15	3	69	100	28	22	34	12
Zfp36l2	ENSMUSG00000045817	834	75	1082	70	960	66	1042	116
Zfp385b	ENSMUSG00000027016	105	21	153	38	106	21	148	29
Zfp948	ENSMUSG00000067931	153	16	226	55	139	21	197	20
Zmat3	ENSMUSG00000027663	267	23	355	37	287	4	337	50

Supplementary Table S6. RNASeq analysis of effects of angiotensin II (AngII) on mRNA expression in hearts from PKN2Het vs WT littermates: mRNAs significantly downregulated by AngII in WT hearts.

Gene Symbol	Ensembl gene id	WT Vehicle		WT AngII		PKN2Het Vehicle		PKN2Het AngII	
		Mean	SD	Mean	SD	Mean	SD	Mean	SD
0610040J01Rik	ENSMUSG00000060512	86	12	50	19	78	10	62	9
1600014C10Rik	ENSMUSG00000054676	741	15	595	54	669	41	652	77
1700123M08Rik	ENSMUSG00000085614	65	5	40	9	59	6	47	7
2010001K21Rik	ENSMUSG00000051606	55	21	18	7	54	24	32	15
2310015K22Rik	ENSMUSG00000101257	17	5	5	2	14	3	6	4
2700097O09Rik	ENSMUSG00000062198	195	23	141	15	172	25	159	20
2900097C17Rik	ENSMUSG00000102869	6799	384	5461	338	6885	478	6050	659
5830417I10Rik	ENSMUSG00000078684	1015	96	743	87	964	152	776	61
A330023F24Rik	ENSMUSG00000096929	201	38	119	33	138	27	115	44
Abcb10	ENSMUSG00000031974	999	42	743	124	1019	76	871	93
Abcb8	ENSMUSG00000028973	1023	86	729	145	1062	134	871	63
Abcd3	ENSMUSG00000028127	2749	274	2096	283	2698	193	2334	334
Abhd10	ENSMUSG00000033157	265	34	203	14	258	14	237	14
AC131339.2	ENSMUSG00000116656	8254	1201	5715	1421	7792	541	6468	972
Acaa2	ENSMUSG00000036880	13252	1380	8259	2391	12324	1059	9258	1215
Acacb	ENSMUSG00000042010	7443	571	5153	1077	7777	973	6190	276
Acad12	ENSMUSG00000042647	1869	179	1262	208	1828	141	1493	156
Acad8	ENSMUSG00000031969	768	60	549	63	690	83	672	41
Acadm	ENSMUSG00000062908	23340	1838	16479	3425	22611	922	17864	1871
Acads	ENSMUSG00000029545	2580	67	1841	326	2630	310	2033	223
Acadsb	ENSMUSG00000030861	2976	193	2400	192	2851	242	2623	299
Acadvl	ENSMUSG00000018574	16703	1281	11802	3040	16035	839	13194	640
Acat1	ENSMUSG00000032047	11595	1306	8601	2045	11394	1310	9863	770
Aco2	ENSMUSG00000022477	41047	2512	30674	4778	41075	2487	35785	2269
Acot2	ENSMUSG00000021226	1314	144	912	240	1349	225	975	53
Acot7	ENSMUSG00000028937	819	45	640	57	804	63	691	52
Acox1	ENSMUSG00000020777	5964	375	4807	434	5983	629	5289	513
Acp6	ENSMUSG00000028093	375	19	285	28	360	30	302	31
Acsl1	ENSMUSG00000018796	21401	1598	15516	3710	21505	1628	18159	1993
Acsl6	ENSMUSG00000020333	322	47	199	38	318	50	264	61
Acsm5	ENSMUSG00000030972	83	17	31	11	61	9	36	8
Acy3	ENSMUSG00000024866	300	15	192	35	310	25	238	34
Adamts7	ENSMUSG00000032363	320	21	222	35	323	29	246	59
Adck1	ENSMUSG00000021044	486	19	381	49	484	24	447	35
Adcy1	ENSMUSG00000020431	128	33	66	23	142	11	108	33
Adhfe1	ENSMUSG00000025911	1463	160	926	241	1480	91	1152	46
Adipor2	ENSMUSG00000030168	2471	47	2151	151	2493	164	2345	116
Adra1b	ENSMUSG00000050541	489	73	272	64	473	98	320	63
Afg1l	ENSMUSG00000038302	1113	135	844	176	1027	95	958	51
Afg3l1	ENSMUSG00000031967	1302	67	991	162	1222	123	1128	81
Afg3l2	ENSMUSG00000024527	3400	269	2686	386	3367	188	3160	98
Agl	ENSMUSG00000033400	6620	574	5262	429	6615	587	5784	471
Agtpbp1	ENSMUSG00000021557	3401	239	2676	267	3243	152	2747	370
Agtr1a	ENSMUSG00000049115	735	120	525	56	733	94	602	75
AI464131	ENSMUSG00000046312	274	30	170	37	300	45	212	57
Ak1	ENSMUSG00000026817	8786	736	6676	1141	8306	245	7478	570
Ak3	ENSMUSG00000024782	2865	206	2428	194	2737	50	2646	143
Akap1	ENSMUSG00000018428	3302	350	2545	516	3554	412	3012	130
Akr1b3	ENSMUSG00000001642	4099	333	3107	569	4024	229	3471	308
Akr1e1	ENSMUSG00000045410	600	74	471	42	501	55	559	71
Akr7a5	ENSMUSG00000028743	466	29	337	42	459	49	383	48
Akt2	ENSMUSG00000004056	3610	431	2539	315	3460	299	2896	149
Aktip	ENSMUSG00000031667	1230	73	958	87	1211	51	1043	104
Aldh5a1	ENSMUSG00000035936	1096	126	715	113	1087	105	893	121
Aldoa	ENSMUSG00000030695	35858	1289	30804	2263	36705	2352	34386	2290
Alkbh5	ENSMUSG00000042650	2176	139	1835	156	2250	270	1962	35
Alkbh7	ENSMUSG00000002661	389	36	284	50	398	51	341	39
Amd1	ENSMUSG00000075232	2921	695	1957	248	2352	376	2046	448
Anapc13	ENSMUSG00000035048	991	52	835	73	936	44	898	50

Ank	ENSMUSG00000022265	4065	84	3017	442	4145	294	3446	202
Ank2	ENSMUSG00000032826	3236	377	2426	262	3245	406	2934	430
Anks1	ENSMUSG00000024219	1418	123	1011	201	1347	177	1118	141
Anxa11	ENSMUSG00000021866	1492	129	1141	185	1489	130	1259	107
Apba3	ENSMUSG00000004931	676	58	507	64	671	72	610	77
Apobec2	ENSMUSG00000040694	3232	195	2680	271	3255	84	2930	192
Arfgap2	ENSMUSG00000027255	1072	50	921	18	1095	72	979	38
Arhgap26	ENSMUSG00000036452	1156	166	889	88	1003	90	905	186
Arhgef17	ENSMUSG00000032875	1622	105	1309	145	1648	171	1396	65
Arhgef19	ENSMUSG00000028919	631	130	395	97	630	134	502	132
Arl2	ENSMUSG00000024944	429	20	345	25	432	32	371	18
Armc2	ENSMUSG00000071324	597	48	445	104	615	73	517	100
Art3	ENSMUSG00000034842	4846	519	3712	598	4603	497	4111	546
Art5	ENSMUSG00000070424	186	40	126	25	137	17	109	25
As3mt	ENSMUSG00000003559	1028	40	757	113	983	51	838	64
Asb18	ENSMUSG00000067081	499	48	355	63	568	74	485	101
Asb8	ENSMUSG00000048175	1440	34	1106	130	1405	108	1243	39
Atp5a1	ENSMUSG00000025428	73890	5007	52519	8597	73310	4969	61155	3605
Atp5b	ENSMUSG00000025393	81983	4027	60525	7907	80175	3926	68956	5579
Atp5d	ENSMUSG00000003072	5810	313	4470	769	5730	539	5074	535
Atp5e	ENSMUSG00000016252	4682	460	3624	408	4707	229	4130	508
Atp5g3	ENSMUSG00000018770	18004	1331	13448	2291	18271	996	15327	1271
Atp5o	ENSMUSG00000022956	14204	841	11103	1882	13615	536	12332	665
Atrip	ENSMUSG00000025646	240	48	178	13	214	7	169	22
Auh	ENSMUSG00000021460	1320	84	937	128	1302	50	1114	103
B4gat1	ENSMUSG00000047379	686	31	550	51	714	60	593	58
Banf2os	ENSMUSG00000086384	37	10	18	9	34	8	23	6
Bap1	ENSMUSG00000021901	906	50	726	88	873	45	830	87
BB218582	ENSMUSG00000085218	104	20	66	19	93	12	74	18
BC025920	ENSMUSG00000074862	63	12	38	10	58	6	40	6
Bcas2	ENSMUSG00000005687	603	47	499	33	607	16	526	34
Bcat2	ENSMUSG00000030826	1208	96	917	126	1160	104	1012	77
Bcl2l13	ENSMUSG00000009112	1949	142	1520	239	2093	293	1695	182
Bsg	ENSMUSG00000023175	18883	1349	15208	1807	18987	778	17057	1251
Btbd2	ENSMUSG00000003344	522	41	395	30	524	83	414	33
Bzw2	ENSMUSG00000020547	2728	225	2062	260	2680	103	2352	189
C030006K11Rik	ENSMUSG00000116138	646	57	430	81	638	91	512	103
C530005A16Rik	ENSMUSG00000085408	50	10	25	6	47	2	34	5
Cacfd1	ENSMUSG00000015488	733	54	563	73	706	29	560	60
Cacnb2	ENSMUSG00000057914	1546	266	1014	149	1369	192	1040	154
Cacng6	ENSMUSG00000078815	34	9	13	6	28	11	13	6
Cadm4	ENSMUSG00000054793	295	26	180	39	288	32	216	22
Calr3	ENSMUSG00000019732	237	30	185	7	218	18	193	10
Cars2	ENSMUSG00000056228	540	40	408	58	563	48	458	53
Ccdc85c	ENSMUSG00000084883	758	52	536	118	780	117	613	80
Ccl11	ENSMUSG00000020676	47	16	18	6	28	8	22	12
Cd59a	ENSMUSG00000032679	1574	127	1303	120	1547	105	1280	69
Cd99l2	ENSMUSG00000035776	1839	81	1579	95	1863	109	1697	59
Cdh2	ENSMUSG00000024304	7013	593	5762	276	7154	806	6155	511
Cdip1	ENSMUSG00000004071	1222	78	994	157	1254	99	1121	69
Cdkl5	ENSMUSG00000031292	275	46	190	15	246	19	225	37
Cdkn1c	ENSMUSG00000037664	381	66	236	40	329	26	256	83
Cds2	ENSMUSG00000058793	4266	154	3435	371	4238	261	3575	214
Cep128	ENSMUSG00000061533	288	24	199	59	257	34	220	53
Cep63	ENSMUSG00000032534	767	47	629	48	738	59	684	37
Ces1d	ENSMUSG00000056973	2184	133	1205	405	1975	180	1372	236
Chchd10	ENSMUSG00000049422	5597	303	4264	732	5732	395	4864	565
Chchd2	ENSMUSG00000070493	5866	649	4709	645	5829	370	5166	564
Chrm2	ENSMUSG00000045613	2913	381	2078	129	2822	350	2290	539
Chrna2	ENSMUSG00000022041	51	20	23	5	42	10	23	6
Cirbp	ENSMUSG00000045193	506	100	381	38	528	31	425	70
Cisd1	ENSMUSG00000037710	2524	194	2038	264	2499	82	2259	149
Clcn3	ENSMUSG00000004319	1219	147	945	108	1163	87	993	110
Clpp	ENSMUSG00000002660	608	48	457	72	598	7	499	95
Clstn1	ENSMUSG00000039953	1769	192	1482	75	1642	58	1584	93
Cluh	ENSMUSG00000020741	5745	385	4121	818	6093	799	5032	263
Cmbl	ENSMUSG00000022235	621	59	470	81	594	54	495	52

Cobll1	ENSMUSG00000034903	1935	295	1391	206	1921	162	1717	245
Cog7	ENSMUSG00000034951	461	24	365	35	450	38	403	33
Colq	ENSMUSG00000057606	238	48	142	30	274	25	195	33
Coq10a	ENSMUSG00000039914	3593	171	2600	450	3601	150	3083	271
Coq2	ENSMUSG00000029319	1358	71	1066	109	1353	65	1154	62
Coq7	ENSMUSG00000030652	1122	100	786	155	1106	99	884	88
Coq9	ENSMUSG00000031782	6190	445	4627	906	6154	410	5544	503
Corin	ENSMUSG00000005220	3746	79	2720	217	3571	374	2889	594
Cox4i1	ENSMUSG00000031818	22284	812	17754	1611	22070	1141	19696	1055
Cox5a	ENSMUSG00000000088	11595	608	8951	1587	11127	414	10060	740
Cox5b	ENSMUSG00000061518	10424	672	8385	1343	10221	301	9039	577
Cox7a1	ENSMUSG00000074218	8954	697	6119	1540	8610	451	7040	827
Cox8b	ENSMUSG00000025488	6504	621	4961	1023	6394	477	5556	903
Cpt2	ENSMUSG00000028607	3334	182	2311	520	3272	298	2688	241
Crat	ENSMUSG00000026853	8544	365	5905	1336	8462	672	6705	567
Cs	ENSMUSG00000005683	24899	2214	17482	3393	24790	1691	20831	1885
Ctnna1	ENSMUSG00000037815	10146	341	7973	731	9700	499	8679	771
Ctsf	ENSMUSG00000083282	632	56	508	41	584	26	503	28
Cul4a	ENSMUSG00000031446	2213	84	1944	75	2181	132	2162	67
Cuta	ENSMUSG00000024194	357	31	286	29	351	28	325	26
Cux2	ENSMUSG00000042589	305	54	194	43	272	63	232	37
Cxadr	ENSMUSG00000022865	914	145	688	47	929	123	924	91
Cyb5d2	ENSMUSG00000057778	378	41	287	32	380	40	322	22
Cyfip2	ENSMUSG00000020340	4903	247	3825	494	5110	622	4425	316
Cyhr1	ENSMUSG00000053929	1686	63	1426	122	1706	105	1462	118
Cyp1a1	ENSMUSG00000032315	14	10	3	1	10	6	3	1
Cyth1	ENSMUSG00000017132	1083	120	785	146	1045	114	943	140
D17H6S53E	ENSMUSG00000043311	205	17	148	28	206	24	200	19
D2hgdh	ENSMUSG00000073609	648	30	491	28	640	53	550	62
D5Ertd579e	ENSMUSG00000029190	3086	284	2426	238	2994	183	2459	146
Dap3	ENSMUSG00000068921	1533	99	1172	166	1441	33	1291	61
Ddt	ENSMUSG00000001666	477	14	371	59	468	31	410	47
Decr1	ENSMUSG00000028223	8479	863	5924	1566	7835	344	6512	520
Dele1	ENSMUSG00000024442	2437	95	1683	363	2478	270	2004	115
Dgat2	ENSMUSG00000030747	4953	461	3276	860	4835	519	3984	545
Dhodh	ENSMUSG00000031730	247	18	159	24	232	20	214	23
Dhrs11	ENSMUSG00000034449	676	56	455	62	690	48	551	73
Dhrs4	ENSMUSG00000022210	1099	57	894	97	1070	35	930	39
Diablo	ENSMUSG00000029433	1178	83	980	91	1146	35	1105	48
Dip2c	ENSMUSG00000048264	1657	88	1255	180	1673	186	1363	98
Dirc2	ENSMUSG00000022848	917	63	720	74	897	73	778	36
Dis3l	ENSMUSG00000032396	666	19	538	31	657	38	594	19
Dlst	ENSMUSG00000004789	11654	750	8617	1895	11270	555	10153	788
Dmpk	ENSMUSG00000030409	5934	219	4888	565	5867	505	5423	498
Dnaaf3	ENSMUSG00000055809	112	12	66	8	98	18	67	9
Dnajb2	ENSMUSG00000026203	1197	57	1005	70	1211	30	1066	80
Dnajb9	ENSMUSG00000014905	945	82	775	43	973	79	828	109
Dnajc28	ENSMUSG00000039763	1292	140	949	125	1268	112	1025	90
Doc2g	ENSMUSG00000024871	2878	347	2069	419	3127	217	2451	320
Drosha	ENSMUSG00000022191	1389	86	1151	83	1380	106	1191	65
Dsc2	ENSMUSG00000024331	1098	132	758	132	1038	80	880	140
Dusp18	ENSMUSG00000047205	1193	123	695	186	1165	123	828	151
Dusp23	ENSMUSG00000026544	177	25	131	14	183	13	153	7
Dym	ENSMUSG00000035765	1390	74	1135	99	1419	90	1253	76
Dynll2	ENSMUSG00000020483	8450	781	6147	789	8457	316	7374	749
E2f6	ENSMUSG00000057469	1947	96	1553	144	1933	82	1731	69
Ech1	ENSMUSG00000053898	18987	2037	11742	3859	18009	1245	12749	1792
Echdc3	ENSMUSG00000039063	427	19	300	49	428	30	350	42
Echs1	ENSMUSG00000025465	4828	268	3779	465	4896	284	4167	357
Eci1	ENSMUSG00000024132	3819	262	2675	691	3634	65	3027	306
Ecpas	ENSMUSG00000050812	5520	139	4646	405	5619	641	4989	290
Ecsit	ENSMUSG00000066839	1352	92	1050	152	1386	47	1218	79
Eef1a2	ENSMUSG00000016349	19816	1103	16353	1766	20590	904	18385	1605
Eef1d	ENSMUSG00000055762	2212	97	1867	139	2077	103	2044	101
Efcab2	ENSMUSG00000026495	2803	355	2211	241	2798	209	2383	245
Efnb3	ENSMUSG00000003934	1231	119	643	238	1266	236	811	246
Egflam	ENSMUSG00000042961	374	53	240	63	279	55	262	40

Egln1	ENSMUSG00000031987	9248	229	6578	1044	9122	656	7541	559
Eid2b	ENSMUSG00000070705	191	16	132	14	196	26	154	37
Eml2	ENSMUSG00000040811	379	40	285	35	408	44	337	30
Endog	ENSMUSG00000015337	455	64	312	72	475	50	367	54
Eno3	ENSMUSG00000060600	20868	2272	14909	3162	20388	959	17554	1469
Enpp5	ENSMUSG00000023960	482	40	367	31	452	24	401	59
Entpd4b	ENSMUSG00000022066	1245	62	1009	104	1249	57	1115	37
Ephx2	ENSMUSG00000022040	5045	182	3683	568	4643	192	4079	372
Epm2a	ENSMUSG00000055493	816	72	609	39	825	73	714	58
Erc1	ENSMUSG00000030172	1343	106	1011	106	1205	149	1073	45
Esrrb	ENSMUSG00000021255	570	46	424	77	607	56	500	36
Esrrg	ENSMUSG00000026610	1115	169	852	111	1188	192	986	138
Etfa	ENSMUSG00000032314	11167	800	7804	1484	10775	533	8929	812
Etfb	ENSMUSG00000004610	8827	431	6069	1637	8450	419	6927	725
Etfdh	ENSMUSG00000027809	13588	811	9757	2314	12812	772	10703	771
Extl1	ENSMUSG00000028838	505	28	354	91	508	68	427	38
Fam131a	ENSMUSG00000050821	452	30	325	65	445	59	370	46
Fam20b	ENSMUSG00000033557	2462	33	2046	249	2441	169	2285	137
Fam210a	ENSMUSG00000038121	5630	318	3910	672	5594	632	4548	152
Farp2	ENSMUSG00000034066	407	27	323	41	400	28	340	23
Fastkd2	ENSMUSG00000025962	921	102	730	94	816	39	814	51
Fbp2	ENSMUSG00000021456	422	72	243	83	363	23	250	9
Fbxo21	ENSMUSG00000032898	802	70	590	81	829	129	684	55
Fbxo31	ENSMUSG00000052934	1386	125	967	187	1389	148	1079	71
Fbxo32	ENSMUSG00000022358	4438	248	3382	496	4609	317	3689	462
Fdft1	ENSMUSG00000021273	812	58	545	97	811	49	633	82
Fem1a	ENSMUSG00000043683	4314	280	3250	616	4473	492	3867	370
Fh1	ENSMUSG00000026526	7500	576	5659	1050	7018	180	6388	586
Fhod3	ENSMUSG00000034295	6968	897	4787	812	6949	852	5943	560
Fign	ENSMUSG00000075324	264	50	152	48	282	77	222	62
Fkbp4	ENSMUSG00000030357	7033	609	4753	1205	6950	342	5630	654
Flad1	ENSMUSG00000042642	928	42	703	107	840	64	785	91
Fmc1	ENSMUSG00000019689	622	55	458	87	580	55	484	71
Fn3k	ENSMUSG00000025175	262	26	173	19	252	25	208	27
Fndc5	ENSMUSG00000001334	7755	469	5429	1330	7876	453	6619	559
Foxo3	ENSMUSG00000048756	1906	138	1489	235	1807	100	1507	232
Foxo4	ENSMUSG00000042903	1026	58	818	81	1020	106	883	76
Foxo6os	ENSMUSG00000084929	123	20	70	22	91	13	97	30
Fsd2	ENSMUSG00000038663	7461	469	5809	1017	7438	469	6588	359
Fth1	ENSMUSG00000024661	24620	1681	21525	749	25292	1474	22765	1350
Fuz	ENSMUSG00000011658	154	17	113	4	143	29	114	25
Fxr2	ENSMUSG00000018765	2087	79	1668	103	2112	177	1949	154
Gart	ENSMUSG00000022962	791	63	621	72	729	62	724	31
Gcsh	ENSMUSG00000034424	1021	36	874	74	1000	34	954	42
Gfm1	ENSMUSG00000027774	4053	240	3035	442	3973	430	3372	259
Gfm2	ENSMUSG00000021666	1266	144	1001	118	1229	127	1128	100
Gfra1	ENSMUSG00000025089	261	28	179	47	276	25	195	40
Ghitm	ENSMUSG00000041028	10888	1003	9249	687	10712	845	9689	126
Glo1	ENSMUSG00000024026	1952	124	1549	162	1830	76	1663	101
Gm10644	ENSMUSG00000074219	30	3	13	5	21	6	18	7
Gm20619	ENSMUSG00000093482	85	17	53	4	91	12	73	11
Gm29170	ENSMUSG00000100455	84	16	48	12	73	11	60	14
Gm33543	ENSMUSG00000110353	38	12	18	6	30	9	24	10
Gm36827	ENSMUSG00000112327	289	42	195	41	280	51	188	31
Gm37829	ENSMUSG00000104453	1373	82	1009	161	1372	115	1141	137
Gm40604	ENSMUSG00000112800	20	10	8	4	11	3	8	3
Gm43672	ENSMUSG00000106019	412	67	247	76	363	75	322	88
Gm45012	ENSMUSG00000109052	77	14	44	18	83	8	50	6
Gm47547	ENSMUSG00000114196	4410	804	3053	766	4397	589	3371	749
Gm49083	ENSMUSG00000115354	278	31	195	62	260	7	202	31
Gm49130	ENSMUSG00000115234	52	12	23	9	54	13	29	9
Gm49477	ENSMUSG00000116066	359	37	242	50	366	16	279	49
Gm826	ENSMUSG00000074623	78	4	53	10	73	9	60	9
Gna12	ENSMUSG00000000149	3048	223	2381	429	3083	284	2577	277
Gnpat	ENSMUSG00000031985	4943	161	3883	434	4842	240	4332	164
Got1	ENSMUSG00000025190	12937	538	8755	1664	12760	639	10615	1253
Got2	ENSMUSG00000031672	15403	947	11510	1822	15607	1320	13207	1045

Gpd2	ENSMUSG00000026827	401	24	334	32	392	48	398	25
Gpn1	ENSMUSG00000064037	443	41	349	55	348	21	379	15
Gpr155	ENSMUSG00000041762	432	52	326	29	393	31	316	35
Gpr22	ENSMUSG00000044067	1368	315	717	244	1339	221	933	230
Gpr27	ENSMUSG00000072875	177	17	110	8	189	36	153	20
Gpt	ENSMUSG00000022546	376	38	239	62	389	47	298	58
Grb14	ENSMUSG00000026888	3020	238	2239	419	3074	220	2499	294
Gsta4	ENSMUSG00000032348	1102	100	835	91	1063	51	979	106
Gstk1	ENSMUSG00000029864	1045	99	635	177	1034	85	737	172
Gstm1	ENSMUSG00000058135	2900	228	2007	410	2765	233	2247	280
Gstm7	ENSMUSG00000004035	451	36	268	66	397	64	313	83
Gstp1	ENSMUSG00000060803	1530	60	1249	118	1564	76	1343	113
Gstt1	ENSMUSG00000001663	186	22	128	20	182	23	129	13
Gypc	ENSMUSG00000090523	589	35	453	36	560	48	489	47
Gys1	ENSMUSG00000003865	2308	136	1765	214	2462	310	2076	150
Gzmm	ENSMUSG00000054206	67	4	40	12	60	12	51	9
H2-Ke6	ENSMUSG00000073422	624	79	486	54	621	40	499	38
H2afv	ENSMUSG00000041126	582	47	479	48	560	29	511	53
Hadh	ENSMUSG00000027984	9800	579	6798	1554	9433	502	7758	928
Hadhb	ENSMUSG00000059447	37787	3939	27391	5861	37795	3637	30089	2124
Hbs1l	ENSMUSG00000019977	1596	64	1363	99	1582	55	1431	99
Hcn4	ENSMUSG00000032338	215	29	145	22	226	22	167	29
Hdhd2	ENSMUSG00000025421	925	39	718	100	905	51	782	86
Heatr5b	ENSMUSG00000039414	1026	107	806	74	965	134	837	66
Helt	ENSMUSG00000047171	27	9	8	2	21	5	16	10
Herc3	ENSMUSG00000029804	1105	188	784	144	1110	191	995	112
Hibadh	ENSMUSG00000029776	4598	354	3506	554	4704	186	4030	384
Hikeshi	ENSMUSG00000062797	431	18	351	14	437	49	395	29
Hk2	ENSMUSG00000000628	7061	664	5062	953	7161	711	6174	483
Hlf	ENSMUSG00000003949	702	180	377	124	587	150	396	181
Hmgcs2	ENSMUSG00000027875	274	33	184	30	225	17	152	50
Hnmt	ENSMUSG00000026986	222	26	145	18	233	31	181	38
Hopx	ENSMUSG00000059325	3360	233	2164	627	3438	319	2591	663
Hrc	ENSMUSG00000038239	19722	920	12511	2864	19104	720	15198	1393
Hsd17b10	ENSMUSG00000025260	1979	163	1487	242	1984	101	1624	232
Hsdl2	ENSMUSG00000028383	5524	757	4049	946	5605	677	4510	583
Hspa5	ENSMUSG00000026864	12991	1134	9421	1954	11342	633	10018	1044
Hspa9	ENSMUSG00000024359	16027	1269	12152	2141	15522	684	14164	919
Hspd1	ENSMUSG00000025980	8960	680	6784	1395	8344	426	7562	640
Htra1	ENSMUSG00000006205	1381	97	1023	118	1387	133	1174	135
Iars2	ENSMUSG00000026618	2409	99	1873	136	2398	100	2171	120
Idh2	ENSMUSG00000030541	25832	1566	18548	3665	26906	2049	21457	2197
Idh3a	ENSMUSG00000032279	10211	1028	7707	867	10029	629	8753	799
Idh3b	ENSMUSG00000027406	11455	1144	8621	1973	11227	459	9312	738
Ids	ENSMUSG00000035847	844	113	686	22	705	81	678	78
Idua	ENSMUSG00000033540	381	15	288	34	343	21	312	19
Il10rb	ENSMUSG00000022969	2740	90	2327	186	2766	76	2592	101
Immt	ENSMUSG00000052337	11137	723	8672	1433	10924	555	9873	547
Imp3	ENSMUSG00000032288	386	37	293	37	353	24	333	39
Insyn1	ENSMUSG00000066607	332	30	244	41	353	22	296	17
Isca1	ENSMUSG00000044792	5523	385	4556	545	5614	212	5191	315
Iscu	ENSMUSG00000025825	1538	101	1296	84	1533	74	1368	132
Isoc2a	ENSMUSG00000086784	619	51	458	90	619	73	511	50
Kars	ENSMUSG00000031948	2130	77	1818	133	2087	64	1922	99
Kcnb1	ENSMUSG00000050556	1529	86	1149	198	1362	171	1226	75
Kcnd3	ENSMUSG00000040896	419	52	304	54	437	49	338	38
Kcng2	ENSMUSG00000059852	1131	114	738	153	1222	266	957	140
Kcnip2	ENSMUSG00000025221	2873	266	2038	321	2700	318	2226	310
Kcnk3	ENSMUSG00000049265	4069	294	2872	660	4507	692	3530	463
Kctd9	ENSMUSG00000034327	1736	135	1293	231	1770	104	1588	97
Khdrbs3	ENSMUSG00000022332	876	67	638	92	898	73	738	119
Kif16b	ENSMUSG00000038844	1579	142	1284	141	1624	143	1458	52
Kif1c	ENSMUSG00000020821	13040	401	10864	1290	13509	1540	11810	481
Kif21a	ENSMUSG00000022629	911	67	703	90	899	40	823	111
Klf9	ENSMUSG00000033863	2073	327	1580	218	1929	244	1672	332
Klhl21	ENSMUSG00000073700	1596	96	1232	219	1629	106	1373	96
Klhl33	ENSMUSG00000090799	489	94	262	86	446	37	313	87

Kpna6	ENSMUSG00000003731	2025	137	1642	186	1978	88	1813	64
Ky	ENSMUSG00000035606	445	33	251	85	458	49	367	57
Kyat1	ENSMUSG00000039648	379	27	294	42	380	16	314	20
L2hgdh	ENSMUSG00000020988	1075	56	746	101	1069	51	876	104
Lclat1	ENSMUSG00000054469	3111	302	2399	376	3091	319	2588	234
Ldhb	ENSMUSG00000030246	22959	1639	15955	3136	22470	933	18647	1684
Letm1	ENSMUSG00000005299	2120	191	1513	340	2063	206	1803	214
Lias	ENSMUSG00000029199	931	93	751	90	903	34	834	53
Limch1	ENSMUSG00000037736	5052	422	3923	326	5049	205	4581	477
Lingo3	ENSMUSG00000051067	229	41	120	47	252	73	159	48
Lmbrd1	ENSMUSG00000073725	1143	53	960	76	1105	87	1035	39
Lmo7	ENSMUSG00000033060	8272	652	6224	773	7881	612	7367	993
Lonp1	ENSMUSG00000041168	2317	80	2025	163	2376	150	2252	87
Lonrf2	ENSMUSG00000048814	202	35	126	24	204	39	153	25
Lpin1	ENSMUSG00000020593	4444	676	2968	594	4769	532	3724	386
Lpl	ENSMUSG00000015568	90429	5374	71420	8174	93258	8317	80525	6094
Lrpprc	ENSMUSG00000024120	4237	451	2986	400	4181	326	3537	377
Lrrc3b	ENSMUSG00000045201	506	44	333	72	477	50	367	53
Lsm14b	ENSMUSG00000039108	972	35	742	85	914	50	896	76
Lynx1	ENSMUSG00000022594	5026	322	3722	716	5034	369	4308	264
Maf1	ENSMUSG00000022553	1093	59	891	57	1121	107	1002	47
Magi3	ENSMUSG00000052539	1616	181	1196	121	1556	176	1327	166
Magt1	ENSMUSG00000031232	1232	137	942	126	1216	90	994	120
Malsu1	ENSMUSG00000029815	281	28	208	15	260	23	236	30
Map10	ENSMUSG00000050930	149	21	98	15	151	17	112	10
Map1lc3a	ENSMUSG00000027602	4603	222	3864	305	4713	378	4049	447
Mapk8ip3	ENSMUSG00000024163	1609	148	1286	127	1571	128	1366	102
Mars2	ENSMUSG00000046994	370	47	284	34	364	34	304	25
Mccc1	ENSMUSG00000027709	2112	94	1589	261	2026	95	1662	118
Mdga1	ENSMUSG00000043557	283	40	180	25	255	46	211	43
Mdh2	ENSMUSG00000019179	16876	703	12970	2129	16369	1034	14499	734
Me1	ENSMUSG00000032418	2846	130	2349	226	2697	138	2438	237
Med12l	ENSMUSG00000056476	211	24	139	35	199	27	142	38
Med9	ENSMUSG00000061650	383	8	302	30	383	23	347	23
Mettl7a1	ENSMUSG00000054619	740	30	561	48	684	59	597	63
Mfn1	ENSMUSG00000027668	8217	628	6382	1129	8242	170	7059	753
Mfn2	ENSMUSG00000029020	15414	955	11184	2042	15810	1644	13241	705
Mgme1	ENSMUSG00000027424	350	28	255	46	337	12	267	30
Mgrn1	ENSMUSG00000022517	3397	191	2489	420	3480	322	2787	167
Mhrt	ENSMUSG00000097652	413	27	294	60	414	38	328	38
Miga1	ENSMUSG00000054942	412	61	323	8	409	40	383	35
Mipep	ENSMUSG00000021993	1364	63	983	167	1394	126	1103	71
Mllt6	ENSMUSG00000038437	1839	74	1515	116	1801	105	1533	96
Mlxip	ENSMUSG00000038342	1090	98	876	108	1082	85	963	38
Mmaa	ENSMUSG00000037022	603	23	460	70	572	47	517	37
Mmadhc	ENSMUSG00000026766	1760	184	1400	199	1684	61	1526	84
Mov10l1	ENSMUSG00000015365	912	70	518	82	908	73	735	108
Mpi	ENSMUSG00000032306	1513	105	1202	137	1517	24	1387	104
Mpped2	ENSMUSG00000016386	351	88	229	30	340	24	247	36
Mpst	ENSMUSG00000071711	347	22	269	21	344	35	280	23
Mpv17	ENSMUSG00000107283	1098	32	873	90	1118	80	934	104
Mrpl14	ENSMUSG00000023939	682	54	516	77	739	35	586	87
Mrpl16	ENSMUSG00000024683	961	36	784	75	930	52	848	97
Mrpl28	ENSMUSG00000024181	1210	39	882	132	1171	81	1020	121
Mrpl37	ENSMUSG00000028622	1184	43	904	135	1161	34	1046	74
Mrpl38	ENSMUSG00000020775	704	31	534	72	674	41	611	58
Mrpl39	ENSMUSG00000022889	1365	91	1053	180	1335	40	1087	56
Mrpl4	ENSMUSG00000003299	1028	38	792	138	1029	93	915	137
Mrpl45	ENSMUSG00000018882	1187	97	837	153	1100	58	1043	54
Mrps26	ENSMUSG00000037740	355	39	258	24	351	18	326	26
Mrps35	ENSMUSG00000040112	1312	76	991	219	1236	59	1132	63
Mrps6	ENSMUSG00000039680	361	20	285	16	354	19	300	4
Msrb2	ENSMUSG00000023094	1067	66	775	115	1023	25	865	63
Mtfp1	ENSMUSG00000004748	1636	82	1103	322	1626	114	1320	90
Mtfr1l	ENSMUSG00000046671	2563	65	2169	189	2622	122	2390	105
Mtg2	ENSMUSG00000039069	366	31	275	26	387	9	312	16
Mtmr4	ENSMUSG00000018401	533	95	415	52	508	47	429	38

Mto1	ENSMUSG00000032342	481	27	368	46	450	40	405	41
Mtr	ENSMUSG00000021311	3225	471	2012	528	2736	435	2132	259
Mtus2	ENSMUSG00000029651	2962	248	2267	102	3029	325	2381	425
Myadml2	ENSMUSG00000025141	267	6	178	37	256	34	227	25
Mybbp1a	ENSMUSG00000040463	1302	174	1041	48	1189	167	1099	79
Myh14	ENSMUSG00000030739	1825	190	1436	214	1992	381	1613	123
Myh6	ENSMUSG00000040752	416670	31586	295124	67491	432469	60382	365274	31724
Mylip	ENSMUSG00000038175	408	59	322	55	361	21	336	42
Myzap	ENSMUSG00000041361	8078	741	6069	777	8163	545	7067	721
Nampt	ENSMUSG00000020572	4880	529	3965	206	4631	388	3944	359
Nbas	ENSMUSG00000020576	829	71	596	58	720	104	703	64
Ndrg2	ENSMUSG00000004558	19416	536	16383	1310	19419	1235	17364	725
Ndufa10	ENSMUSG00000026260	9566	706	7436	1132	9920	331	8450	886
Ndufa8	ENSMUSG00000026895	4878	294	3771	635	4757	148	4279	331
Ndufa9	ENSMUSG00000000399	9480	996	7084	1337	9287	369	7867	703
Ndufs1	ENSMUSG00000025968	15975	1815	11734	2163	15650	1579	13236	1027
Ndufs2	ENSMUSG00000013593	16031	729	11634	1773	15858	991	13476	1336
Ndufs3	ENSMUSG00000005510	6047	349	4620	872	5917	121	5197	447
Ndufs7	ENSMUSG00000020153	3525	187	2485	513	3595	317	2923	370
Ndufv1	ENSMUSG00000037916	9773	762	6974	1216	9669	615	8148	626
Nectin2	ENSMUSG00000062300	413	36	310	34	417	41	335	50
Nek9	ENSMUSG00000034290	4497	135	3774	292	4482	346	4215	107
Nfe2l1	ENSMUSG00000038615	10454	393	8704	788	10418	916	9618	241
Nfs1	ENSMUSG00000027618	1552	137	1235	133	1562	117	1353	125
Ngrn	ENSMUSG00000047084	316	43	243	18	297	10	268	29
Nmnat3	ENSMUSG00000032456	318	32	227	37	294	26	237	22
Nomo1	ENSMUSG00000030835	2843	188	2392	196	2879	272	2584	198
Npepps	ENSMUSG00000001441	2325	152	1944	99	2270	128	2110	152
Nprl2	ENSMUSG00000010057	233	23	177	25	218	16	188	17
Nqo2	ENSMUSG00000046949	946	76	768	84	944	37	822	27
Nr1d1	ENSMUSG00000020889	2205	482	1594	392	2278	416	1686	114
Nr3c2	ENSMUSG00000031618	554	97	352	33	533	70	406	87
Nsmce1	ENSMUSG00000030750	572	26	425	55	506	32	480	49
Nsun4	ENSMUSG00000028706	668	48	514	69	656	35	592	35
Nt5c1a	ENSMUSG00000054958	221	21	132	21	214	28	180	22
Nt5dc3	ENSMUSG00000054027	1591	190	1169	296	1701	183	1426	165
Ntn1	ENSMUSG00000020902	1895	146	1435	153	2064	197	1793	251
Nudc	ENSMUSG00000028851	1219	28	997	119	1222	68	1116	61
Nudt3	ENSMUSG00000024213	2420	197	1955	179	2428	117	2144	103
Nudt6	ENSMUSG00000050174	240	31	183	19	270	13	214	24
Oat	ENSMUSG00000030934	4023	214	3428	322	3893	236	3570	97
Ogdh	ENSMUSG00000020456	39640	2492	29734	4328	41635	5116	35518	902
Oma1	ENSMUSG00000035069	520	27	382	67	517	40	433	46
Opa1	ENSMUSG00000038084	5644	514	4210	706	5735	550	5001	562
Oplah	ENSMUSG00000022562	803	117	550	66	755	147	645	45
Optn	ENSMUSG00000026672	2167	261	1708	294	2308	86	2033	94
Osbp	ENSMUSG00000024687	2871	112	2475	227	3049	216	2682	119
Osbpl2	ENSMUSG00000039050	924	46	750	55	906	59	792	22
Osbpl6	ENSMUSG00000042359	560	64	401	56	579	105	481	24
Osgep	ENSMUSG00000006289	680	53	499	68	589	47	573	41
Otud4	ENSMUSG00000036990	1969	261	1551	87	1916	144	1630	131
Oxa1l	ENSMUSG00000000959	1341	57	1036	150	1356	195	1142	95
Oxct1	ENSMUSG00000022186	26538	1570	20521	2412	25456	2358	23051	1272
Oxnad1	ENSMUSG00000021906	1147	104	870	148	1159	96	1055	90
Pacsin2	ENSMUSG00000016664	4608	491	3453	568	4770	416	3975	269
Pccb	ENSMUSG00000032527	1749	37	1306	178	1783	212	1458	93
Pcp4l1	ENSMUSG00000038370	3448	91	2643	293	3460	188	3005	313
Pde2a	ENSMUSG00000110195	1585	126	1282	143	1439	108	1257	211
Pdf	ENSMUSG00000078931	565	42	418	72	575	29	492	19
Pdk2	ENSMUSG00000038967	7045	426	4922	991	7351	661	5815	537
Pdss2	ENSMUSG00000038240	428	19	288	48	415	44	365	47
Pdzd2	ENSMUSG00000022197	1958	240	1387	312	1714	300	1447	149
Peg13	ENSMUSG00000106847	1266	135	996	129	1259	67	1062	102
Perm1	ENSMUSG00000078486	6061	537	4239	948	6388	499	5164	309
Pex10	ENSMUSG00000029047	216	17	157	21	236	16	189	19
Pex6	ENSMUSG00000002763	536	39	383	52	543	100	441	44
Pfkl	ENSMUSG00000020277	1893	156	1558	139	1871	165	1710	80

Pfkm	ENSMUSG00000033065	18225	1341	12907	1890	18352	1337	14993	396
Pgm5	ENSMUSG00000041731	2496	117	1972	181	2577	106	2310	218
Phf20	ENSMUSG00000038116	645	141	463	64	547	79	484	111
Phkg1	ENSMUSG00000025537	414	78	217	95	351	52	195	50
Phpt1	ENSMUSG00000036504	592	39	477	58	591	19	515	20
Phyh	ENSMUSG00000026664	9819	550	7600	1012	9908	282	8331	547
Pigg	ENSMUSG00000029263	357	15	277	13	360	32	317	44
Pigv	ENSMUSG00000043257	190	34	113	16	185	35	150	33
Pik3r4	ENSMUSG00000032571	510	63	421	32	453	52	451	38
Pim3	ENSMUSG00000035828	1043	153	638	156	989	141	713	162
Pip4k2c	ENSMUSG00000025417	839	38	718	27	859	49	781	35
Pitpnc1	ENSMUSG00000040430	2019	131	1451	306	1945	148	1677	225
Pitrm1	ENSMUSG00000021193	1183	91	917	87	1076	97	982	47
Plin4	ENSMUSG00000002831	3263	103	2596	321	3280	102	2808	366
Plpbp	ENSMUSG00000031485	1297	158	1038	125	1269	103	1218	88
Pmpca	ENSMUSG00000026926	2809	172	2292	228	2800	157	2538	100
Pnpla2	ENSMUSG00000025509	4617	330	3361	654	4556	476	3773	317
Poldip2	ENSMUSG00000001100	2037	140	1588	200	2103	131	1870	118
Polr2b	ENSMUSG00000029250	1491	97	1240	88	1445	75	1325	88
Polr2e	ENSMUSG00000004667	833	69	697	28	814	44	761	49
Polr2m	ENSMUSG00000032199	4202	139	3527	289	4270	294	3840	106
Ppara	ENSMUSG00000022383	730	69	463	119	639	77	525	69
Ppm1k	ENSMUSG00000037826	2243	337	1519	318	2006	174	1607	419
Ppp1r12b	ENSMUSG00000073557	7349	246	5778	1053	8073	1048	6703	244
Ppp1r26	ENSMUSG00000035829	123	33	63	14	99	6	69	7
Ppp1r3d	ENSMUSG00000049999	323	31	243	23	344	39	274	29
Ppp5c	ENSMUSG00000003099	1134	58	913	91	1135	59	1032	79
Ppt2	ENSMUSG00000015474	570	20	441	69	579	54	505	43
Prdx6	ENSMUSG00000026701	3412	145	2618	272	3250	102	2855	190
Prkaca	ENSMUSG00000005469	4064	153	3118	405	4060	252	3598	192
Prkag1	ENSMUSG00000067713	1480	97	1136	121	1449	53	1228	110
Prkce	ENSMUSG00000045038	1038	123	798	114	1083	139	932	92
Prpf19	ENSMUSG00000024735	3570	220	2865	389	3749	422	3240	103
Prpf8	ENSMUSG00000020850	3805	235	3158	312	3806	301	3465	198
Psap	ENSMUSG00000004207	20301	713	16734	1671	20510	1354	18075	1122
Psmd2	ENSMUSG00000006998	4504	254	3688	415	4294	293	4129	211
Ptcd3	ENSMUSG00000063884	3497	444	2527	522	3299	255	2951	253
Ptov1	ENSMUSG00000038502	994	34	794	32	947	73	875	61
Ptpn3	ENSMUSG00000038764	1516	118	1158	191	1433	94	1204	64
Pttg1	ENSMUSG00000020415	1218	104	917	152	1220	46	1000	82
Pxmp2	ENSMUSG00000029499	803	44	554	114	790	47	628	75
Pygm	ENSMUSG00000032648	19355	1268	13869	2475	19815	1520	16110	975
Qdpr	ENSMUSG00000015806	905	53	716	59	901	38	833	92
Qsox2	ENSMUSG00000036327	354	29	255	33	340	26	293	34
R3hdm4	ENSMUSG00000035781	843	29	689	82	840	52	717	45
Rab12	ENSMUSG00000023460	2932	232	2405	100	2976	158	2577	278
Rab28	ENSMUSG00000029128	1501	127	1261	120	1546	89	1421	64
Rab3a	ENSMUSG00000031840	556	31	385	62	576	29	459	63
Rai2	ENSMUSG00000043518	607	58	481	89	647	60	562	52
Ralgapa2	ENSMUSG00000037110	2617	245	1978	383	2581	274	2195	200
Ralgapb	ENSMUSG00000027652	1478	136	1208	120	1466	153	1287	73
Rapsn	ENSMUSG00000002104	164	14	112	16	166	20	118	21
Rasd2	ENSMUSG00000034472	85	16	52	8	87	5	67	16
Rbbp5	ENSMUSG00000026439	503	77	394	43	462	27	433	19
Rbfa	ENSMUSG00000024570	537	27	421	57	552	37	476	23
Rbm20	ENSMUSG00000043639	4345	353	3333	538	4405	474	3671	314
Rbm24	ENSMUSG00000038132	2769	152	2054	213	2701	211	2285	323
Rbpms	ENSMUSG00000031586	2091	178	1725	171	2143	145	1933	119
Rdm1	ENSMUSG00000010362	355	44	260	27	395	55	308	50
Retnla	ENSMUSG00000061100	77	26	28	12	70	10	47	28
Retsat	ENSMUSG00000056666	937	102	713	90	893	134	765	34
Rgs7	ENSMUSG00000026527	36	12	15	6	19	3	25	6
Rhobtb2	ENSMUSG00000022075	496	77	385	66	531	42	452	21
Rhot2	ENSMUSG00000025733	3078	130	2175	480	3032	157	2480	193
Rmnd1	ENSMUSG00000019763	878	53	704	63	926	55	800	97
Rnf114	ENSMUSG00000006418	1224	48	1047	46	1232	102	1117	38
Rpa1	ENSMUSG00000000751	1034	51	817	60	1036	89	910	49

Rpap1	ENSMUSG00000034032	295	38	221	36	319	44	271	27
Rpusd4	ENSMUSG00000032044	283	14	221	31	291	18	238	21
Rragd	ENSMUSG00000028278	3170	310	2413	232	3220	162	2839	371
Rrn3	ENSMUSG00000022682	1172	63	954	93	1149	64	1040	78
Rtn4ip1	ENSMUSG00000019864	1524	104	1118	239	1538	112	1315	140
Rufy1	ENSMUSG00000020375	709	60	558	43	649	12	610	48
Rxrg	ENSMUSG00000015843	1040	79	760	162	1102	94	896	96
Samm50	ENSMUSG00000022437	4316	204	3494	463	4136	107	3926	74
Sbk1	ENSMUSG00000042978	1288	124	1004	171	1329	89	1218	118
Scgb1c1	ENSMUSG00000038801	156	23	101	19	163	23	131	27
Scn4a	ENSMUSG00000001027	691	157	407	125	675	132	480	75
Scn4b	ENSMUSG00000046480	1055	187	607	202	976	267	669	172
Scrn3	ENSMUSG00000008226	772	46	634	36	772	89	686	45
Sdhb	ENSMUSG00000009863	12188	749	9065	1722	11819	503	9978	990
Sdhc	ENSMUSG00000058076	7127	66	5010	729	7101	434	5730	395
Sdr39u1	ENSMUSG00000022223	1168	90	858	177	1171	36	977	118
Sel1l	ENSMUSG00000020964	1913	94	1550	157	1805	94	1646	186
Sesn1	ENSMUSG00000038332	2443	189	2090	127	2405	179	2161	117
Sipa1l2	ENSMUSG00000001995	1430	165	1122	105	1540	153	1327	56
Sirt5	ENSMUSG00000054021	574	24	464	39	573	30	507	40
Slc16a7	ENSMUSG00000020102	227	56	144	27	216	67	189	39
Slc25a11	ENSMUSG00000014606	8077	561	5928	1242	7992	341	6986	662
Slc25a12	ENSMUSG00000027010	4184	351	2893	395	4194	379	3611	151
Slc25a13	ENSMUSG00000015112	2989	324	2199	145	3029	276	2574	268
Slc25a20	ENSMUSG00000032602	3086	349	2262	477	3063	140	2521	210
Slc25a3	ENSMUSG00000061904	25015	1211	18063	2479	25124	1400	20806	1485
Slc25a33	ENSMUSG00000028982	271	36	196	32	254	25	214	18
Slc26a6	ENSMUSG00000023259	159	5	105	24	133	7	122	15
Slc2a4	ENSMUSG00000018566	6195	156	4116	759	6192	600	4937	362
Slc36a2	ENSMUSG00000020264	315	40	215	36	309	9	239	36
Slc38a3	ENSMUSG00000010064	939	97	638	211	1037	121	746	126
Slc41a1	ENSMUSG00000013275	1492	118	1131	142	1374	124	1187	145
Slc4a4	ENSMUSG00000060961	964	160	696	87	861	146	774	132
Slc7a1	ENSMUSG00000041313	1469	249	990	213	1397	281	1151	217
Slc9a8	ENSMUSG00000039463	533	54	422	27	481	53	424	37
Slco3a1	ENSMUSG00000025790	1897	213	1577	239	1938	213	1809	168
Smco1	ENSMUSG00000046345	280	33	173	42	270	15	222	35
Smg5	ENSMUSG00000001415	1134	42	904	93	1081	79	1020	136
Smim11	ENSMUSG00000051989	669	30	545	65	628	41	590	28
Snai3	ENSMUSG00000006587	49	18	24	5	45	14	35	8
Snrpn	ENSMUSG00000102252	939	55	661	136	989	67	760	142
Sod1	ENSMUSG00000022982	3578	226	3002	251	3367	134	3215	151
Sod2	ENSMUSG00000006818	12266	831	8665	1885	11982	506	9979	664
Sord	ENSMUSG00000027227	4541	245	3023	680	4444	324	3516	336
Spr	ENSMUSG00000033735	665	53	505	56	683	71	580	44
Spsb1	ENSMUSG00000039911	519	41	383	46	499	68	431	61
Sptb	ENSMUSG00000021061	3814	253	2892	391	4015	448	3358	226
St3gal3	ENSMUSG00000028538	1047	50	840	121	1048	65	941	63
St6galnac6	ENSMUSG00000026811	1129	64	895	132	1127	72	981	112
Stard10	ENSMUSG00000030688	330	75	189	43	297	12	242	66
Stard7	ENSMUSG00000027367	3728	299	2766	387	3568	81	3253	316
Stk11	ENSMUSG00000003068	1448	37	1205	94	1447	59	1293	70
Ston2	ENSMUSG00000020961	279	82	178	41	317	59	217	35
Stub1	ENSMUSG00000039615	1096	59	905	66	1104	71	1014	92
Suclg1	ENSMUSG00000052738	6364	514	4599	1021	6012	279	5327	363
Suclg2	ENSMUSG00000061838	4105	321	2961	428	4037	119	3294	228
Suox	ENSMUSG00000049858	473	53	354	36	448	32	405	21
Susd6	ENSMUSG00000021133	2324	150	1943	183	2364	108	2034	109
Svip	ENSMUSG00000074093	948	93	733	96	873	98	769	67
Swsap1	ENSMUSG00000051238	126	10	90	15	118	11	105	11
Syt7	ENSMUSG00000024743	873	100	595	126	831	116	700	57
Tango2	ENSMUSG00000013539	2372	272	1812	121	2322	147	1927	149
Tango6	ENSMUSG00000041949	231	19	170	17	230	31	201	14
Tars2	ENSMUSG00000028107	667	59	489	66	606	23	549	66
Tarsl2	ENSMUSG00000030515	1088	105	839	102	1083	60	931	19
Taz	ENSMUSG00000009995	683	45	574	36	641	57	573	45
Tbrg4	ENSMUSG00000000384	909	70	673	136	897	113	763	112

Tcaim	ENSMUSG00000046603	1091	140	752	164	1078	129	865	134
Tcap	ENSMUSG00000007877	15023	5676	9524	3481	11808	3513	10365	2562
Tcp11l2	ENSMUSG00000020034	2121	130	1478	128	2131	148	1765	222
Tecrl	ENSMUSG00000049537	1305	197	1030	90	1191	128	1094	181
Tent4b	ENSMUSG00000036779	610	55	482	40	562	52	508	45
Tesc	ENSMUSG00000029359	1119	84	854	76	1160	95	937	103
Thrb	ENSMUSG00000021779	495	72	351	62	440	76	311	43
Tmbim6	ENSMUSG00000023010	3804	173	3339	158	3817	189	3501	211
Tmc7	ENSMUSG00000042246	203	26	121	17	221	36	169	29
Tmem135	ENSMUSG00000039428	983	73	725	115	985	79	815	63
Tmem143	ENSMUSG00000002781	1504	67	919	258	1446	221	1083	117
Tmem177	ENSMUSG00000036975	283	12	189	34	271	12	216	21
Tmem250-ps	ENSMUSG00000087679	1255	42	1017	136	1234	98	1140	53
Tmem38a	ENSMUSG00000031791	7478	344	5801	950	7515	482	6828	346
Tmem50b	ENSMUSG00000022964	927	63	728	73	909	32	769	105
Tmem70	ENSMUSG00000025940	1686	231	1310	188	1682	176	1404	135
Tmem82	ENSMUSG00000043085	271	51	168	43	257	20	187	17
Tmem94	ENSMUSG00000020747	1735	172	1255	258	1762	215	1313	178
Tmod1	ENSMUSG00000028328	6866	511	5456	542	6671	424	6180	413
Tmod4	ENSMUSG00000005628	378	37	254	60	361	43	317	59
Tnfaip8	ENSMUSG00000062210	1646	269	1190	267	1502	102	1310	232
Tnip3	ENSMUSG00000044162	52	11	27	13	47	4	42	4
Tnni3	ENSMUSG00000035458	63108	4479	42722	10485	61514	1321	48873	5226
Tnnt2	ENSMUSG00000026414	110161	8926	87055	9068	105408	3834	92241	8335
Tom1l2	ENSMUSG00000000538	2948	122	2426	287	2928	258	2694	186
Trabd2b	ENSMUSG00000070867	3038	207	2320	372	3187	181	2778	256
Tsc22d1	ENSMUSG00000022010	4439	536	3676	350	4405	178	4019	379
Tspan3	ENSMUSG00000032324	2417	116	2074	132	2505	165	2302	52
Tspyl4	ENSMUSG00000039485	376	62	276	18	382	85	298	73
Ttc19	ENSMUSG00000042298	902	120	684	71	896	69	748	78
Tufm	ENSMUSG00000073838	3132	215	2311	479	3147	191	2714	265
Twnk	ENSMUSG00000025209	604	21	452	67	607	59	505	23
Txnrd2	ENSMUSG00000075704	527	35	362	47	464	30	431	49
Ubac2	ENSMUSG00000041765	482	28	351	27	440	56	380	38
Ubl7	ENSMUSG00000055720	695	84	565	67	663	44	641	37
Ubr2	ENSMUSG00000023977	2578	83	1942	289	2546	196	2215	196
Ucp3	ENSMUSG00000032942	1264	355	659	227	1281	331	698	271
Unc45b	ENSMUSG00000018845	3432	81	2694	372	3138	382	2858	156
Uqcc1	ENSMUSG00000005882	3454	270	2476	553	3363	150	2909	228
Uqcrc1	ENSMUSG00000025651	15130	710	10190	2080	15168	907	12233	1293
Uqcrfs1	ENSMUSG00000038462	13197	914	10056	1838	12772	692	11183	890
Urod	ENSMUSG00000028684	800	46	616	62	756	47	678	29
Usf2	ENSMUSG00000058239	1261	122	1046	81	1203	37	1081	60
Vcp	ENSMUSG00000028452	9696	493	8255	496	9439	494	9118	332
Vdac1	ENSMUSG00000020402	17517	801	13326	1578	17010	633	14751	342
Vdac3	ENSMUSG00000008892	6247	446	4930	675	5939	119	5490	330
Vegfb	ENSMUSG00000024962	2831	191	2209	301	2977	265	2434	179
Vps26a	ENSMUSG00000020078	1810	210	1524	43	1767	104	1632	155
Vwa8	ENSMUSG00000058997	6184	159	4148	762	5901	635	4704	407
Wbp2	ENSMUSG00000034341	1379	72	1162	115	1438	101	1294	92
Wdtc1	ENSMUSG00000037622	1386	86	1042	217	1413	154	1245	104
Wfs1	ENSMUSG00000039474	1806	82	1416	252	1797	191	1634	135
Whrn	ENSMUSG00000039137	481	110	307	100	542	79	382	97
Wnk2	ENSMUSG00000037989	1441	166	821	252	1510	204	1013	246
Ybx1	ENSMUSG00000028639	11917	513	10296	764	11978	639	10588	490
Ywhae	ENSMUSG00000020849	5973	224	5142	422	5882	295	5570	239
Zadh2	ENSMUSG00000049090	1687	130	1330	146	1727	133	1433	161
Zfp113	ENSMUSG00000037007	207	26	137	20	206	31	160	32
Zfp536	ENSMUSG00000043456	134	18	70	15	120	18	92	13
Zfp629	ENSMUSG00000045639	654	44	510	45	651	90	565	56
Zfyve21	ENSMUSG00000021286	406	15	295	34	394	31	309	42
Znrf1	ENSMUSG00000033545	850	52	714	48	788	71	705	40

Supplementary Table S7. RNASeq analysis of effects of angiotensin II (AngII) on mRNA expression in hearts from PKN2Het vs WT littermates: mRNAs significantly upregulated by AngII in PKN2Het hearts.

Gene Symbol	Ensembl gene id	WT Vehicle		WT AngII		PKN2Het Vehicle		PKN2Het AngII	
		Mean	SD	Mean	SD	Mean	SD	Mean	SD
1500004A13Rik	ENSMUSG00000098912	28	5	38	8	20	7	48	20
4931406P16Rik	ENSMUSG00000066571	1213	197	1375	110	1210	171	1441	195
6430584L05Rik	ENSMUSG00000108228	2	1	7	2	3	1	69	99
AC165271.1	ENSMUSG00000116641	7	2	16	5	5	2	19	12
Adgrd1	ENSMUSG00000044017	191	51	269	56	169	19	249	49
Akap2	ENSMUSG00000038729	3768	234	4279	435	3521	379	4095	258
Aldh18a1	ENSMUSG00000025007	242	27	280	13	215	33	299	35
Ano6	ENSMUSG00000064210	778	83	929	100	694	56	876	34
Anp32b	ENSMUSG00000028333	1235	54	1423	104	1216	59	1418	76
Arhgef12	ENSMUSG00000059495	6349	600	6661	1008	6125	289	7218	254
Arrb1	ENSMUSG00000018909	877	101	1086	152	816	90	1086	112
Atp8b2	ENSMUSG00000060671	531	139	704	103	413	28	673	34
Atxn1l	ENSMUSG00000069895	724	111	785	64	708	86	852	141
B3gnt3	ENSMUSG00000031803	234	39	304	15	213	12	289	30
C5ar1	ENSMUSG00000049130	107	17	173	40	78	18	156	56
Capn2	ENSMUSG00000026509	2021	126	2295	152	1873	94	2199	142
Ccl12	ENSMUSG00000035352	28	12	76	30	20	5	61	36
Ccl2	ENSMUSG00000035385	56	15	106	46	42	6	111	80
Cd302	ENSMUSG00000060703	206	18	297	71	176	17	266	37
Cdc42ep4	ENSMUSG00000041598	293	84	306	18	242	49	312	39
Cdc42se1	ENSMUSG00000046722	468	71	617	41	442	53	554	68
Cebpa	ENSMUSG00000034957	116	16	166	28	115	23	164	26
Cenpi	ENSMUSG00000031262	14	6	28	12	6	3	28	9
Ces2e	ENSMUSG00000031886	82	18	89	16	65	19	108	34
Clic5	ENSMUSG00000023959	7647	453	8817	809	7809	595	9603	708
Cmklr1	ENSMUSG00000042190	507	144	618	103	422	100	579	89
Col4a3	ENSMUSG00000079465	203	49	299	93	199	27	307	35
Cyb561	ENSMUSG00000019590	237	62	314	54	200	32	299	78
Cyb5r1	ENSMUSG00000026456	363	22	410	23	351	26	444	45
Dbndd2	ENSMUSG00000017734	265	25	310	15	218	53	285	36
Ddah2	ENSMUSG00000007039	240	14	299	36	214	30	292	28
Ddr2	ENSMUSG00000026674	1034	140	1163	154	869	156	1130	83
Dpysl2	ENSMUSG00000022048	377	57	499	48	329	67	433	23
Dsn1	ENSMUSG00000027635	32	10	50	10	25	6	47	8
Eif4a1	ENSMUSG00000059796	3380	300	3664	494	3084	168	3826	261
Elmo1	ENSMUSG00000041112	303	38	395	22	278	56	438	50
Elovl1	ENSMUSG00000006390	286	23	361	51	246	37	316	43
Enc1	ENSMUSG00000041773	293	76	382	15	273	69	390	41
Erf	ENSMUSG00000040857	309	56	363	54	285	58	372	48
Fabp5	ENSMUSG00000027533	788	95	996	167	674	83	989	37
Fam102b	ENSMUSG00000040339	516	79	657	85	447	67	598	79
Fgf6	ENSMUSG00000000183	19	11	37	18	16	6	40	17
Fkbp5	ENSMUSG00000024222	356	106	469	65	289	55	412	74
Gba	ENSMUSG00000028048	219	25	275	46	194	36	270	21
Gm10275	ENSMUSG00000069682	2119	213	2280	303	1958	234	2331	159
Gm14005	ENSMUSG00000074813	25	6	32	5	23	3	43	5
Gm15542	ENSMUSG00000083396	41	20	74	30	32	14	92	41
Gm21188	ENSMUSG00000095609	24	11	52	38	12	6	39	17
Gm8430	ENSMUSG00000055093	747	208	891	177	614	182	1041	66
Gngt2	ENSMUSG00000038811	179	19	226	24	170	41	224	28
Hbegf	ENSMUSG00000024486	285	26	375	79	293	20	445	97
Hmox1	ENSMUSG00000005413	189	79	278	49	151	19	270	45
Hnrnpf	ENSMUSG00000042079	2929	181	3120	203	2667	95	3177	258
Hnrnpk	ENSMUSG00000021546	4424	263	4608	377	3979	132	4710	317
Hsp90aa1	ENSMUSG00000021270	4809	792	5821	774	4154	437	5635	799
Id3	ENSMUSG00000007872	547	96	629	46	503	70	613	81
Ier5	ENSMUSG00000056708	536	123	783	105	482	61	803	298
Ilk	ENSMUSG00000030890	1549	39	1714	90	1475	117	1685	57
Itpr2	ENSMUSG00000030287	666	151	771	158	613	94	776	101

Jpt1	ENSMUSG00000020737	841	76	987	49	790	37	1002	107
Jpt2	ENSMUSG00000024165	169	19	197	35	141	33	226	44
Lama2	ENSMUSG00000019899	3507	352	3968	325	3362	180	4015	379
Layn	ENSMUSG00000060594	73	12	116	39	72	19	117	25
Lima1	ENSMUSG00000023022	869	137	1044	86	750	124	1003	145
Ly6a	ENSMUSG00000075602	1892	294	2356	309	1658	287	2262	203
Mad2l1	ENSMUSG00000029910	111	20	150	33	90	23	154	23
Map6	ENSMUSG00000055407	108	31	142	36	87	9	129	34
Mcm10	ENSMUSG00000026669	14	6	30	10	8	7	26	18
Mcm3	ENSMUSG00000041859	156	21	214	41	148	27	240	53
Mcm5	ENSMUSG00000005410	84	12	162	45	83	19	169	35
Mcm6	ENSMUSG00000026355	230	32	397	77	216	18	363	70
Mgat5b	ENSMUSG00000043857	8	5	23	15	9	5	31	24
Mmrn2	ENSMUSG00000041445	1117	202	1171	66	966	115	1226	136
Mob3a	ENSMUSG00000003348	134	17	176	20	115	24	176	43
Mybl1	ENSMUSG00000025912	20	8	40	11	22	4	48	10
Myo1c	ENSMUSG00000017774	2939	140	3445	280	2807	136	3414	203
Ncf2	ENSMUSG00000026480	117	30	183	21	107	29	174	52
Nrp2	ENSMUSG00000025969	1954	236	2425	238	1836	224	2427	166
Nuak1	ENSMUSG00000020032	841	90	1047	199	819	26	1165	278
Nudt18	ENSMUSG00000045211	271	21	330	55	259	36	362	63
Numb	ENSMUSG00000021224	620	116	600	28	522	54	673	44
Nusap1	ENSMUSG00000027306	48	17	132	40	45	9	116	36
Ophn1	ENSMUSG00000031214	212	70	246	57	195	73	284	69
Pald1	ENSMUSG00000020092	474	36	537	61	417	42	544	37
Papln	ENSMUSG00000021223	369	42	478	84	367	37	494	57
Parva	ENSMUSG00000030770	1225	61	1409	154	1113	88	1320	96
Pcolce2	ENSMUSG00000015354	412	82	494	81	352	55	503	72
Pdlim1	ENSMUSG00000055044	1237	36	1524	182	1078	86	1607	176
Pecam1	ENSMUSG00000020717	4184	537	5065	334	3964	407	4742	324
Pid1	ENSMUSG00000045658	275	53	338	14	244	37	314	29
Pilra	ENSMUSG00000046245	31	5	52	15	24	9	52	20
Pole2	ENSMUSG00000020974	16	7	31	8	12	5	33	6
Prg4	ENSMUSG00000006014	323	89	484	130	321	33	596	281
Ptx3	ENSMUSG00000027832	9	5	35	23	8	2	42	47
Rab15	ENSMUSG00000021062	19	12	25	4	11	5	30	8
Rad54l	ENSMUSG00000028702	11	9	22	6	6	2	28	11
Ralb	ENSMUSG00000004451	600	34	674	33	559	34	691	29
Raph1	ENSMUSG00000026014	2057	244	2567	254	1871	214	2337	313
Rasa3	ENSMUSG00000031453	585	66	758	73	582	31	773	108
Rhog	ENSMUSG00000073982	297	22	355	35	256	29	324	32
Ripk3	ENSMUSG00000022221	56	11	90	28	34	8	84	24
Rpl3	ENSMUSG00000060036	3377	296	4471	607	3263	335	4320	344
Rpl39	ENSMUSG00000079641	2170	187	2489	536	1860	274	2471	431
Rps27l	ENSMUSG00000036781	985	107	1026	159	889	103	1140	141
Rras	ENSMUSG00000038387	425	64	533	56	410	35	523	72
Sema7a	ENSMUSG00000038264	731	163	865	32	659	93	882	92
Sh3gl1	ENSMUSG00000003200	234	18	296	44	217	29	272	16
Slc39a1	ENSMUSG00000052310	1324	29	1471	82	1256	48	1467	64
Slc3a2	ENSMUSG00000010095	496	50	522	83	462	51	597	75
Sorbs3	ENSMUSG00000022091	627	75	731	50	570	54	681	36
Spsb4	ENSMUSG00000046997	117	24	148	47	116	20	180	48
Srpx	ENSMUSG00000090084	56	2	104	41	56	6	108	42
Thsd1	ENSMUSG00000031480	201	35	239	30	176	24	246	27
Tinagl1	ENSMUSG00000028776	767	115	841	137	702	142	896	129
Troap	ENSMUSG00000032783	9	3	20	8	5	3	20	13
Tspan9	ENSMUSG00000030352	1563	131	1947	401	1621	98	2180	218
Tubb2a	ENSMUSG00000058672	572	55	703	94	505	25	707	148
Tubb6	ENSMUSG00000001473	337	29	445	93	318	39	459	50
Vsig4	ENSMUSG00000044206	33	11	57	21	28	10	77	29
Wdr1	ENSMUSG00000005103	2521	253	2721	220	2501	131	3026	227
Wfdc17	ENSMUSG00000069792	126	27	205	64	117	24	230	60
Ywhab	ENSMUSG00000018326	2036	61	2287	69	1970	113	2268	129
Ywhah	ENSMUSG00000018965	1422	86	1708	173	1342	151	1658	195

Supplementary Table S8. RNASeq analysis of effects of angiotensin II (AngII) on mRNA expression in hearts from PKN2Het vs WT littermates: mRNAs significantly downregulated by AngII in PKN2Het hearts.

Gene Symbol	Ensembl gene id	WT Vehicle		WT AngII		PKN2Het Vehicle		PKN2Het AngII	
		Mean	SD	Mean	SD	Mean	SD	Mean	SD
Abcb7	ENSMUSG00000031333	1213	155	1016	47	1337	171	1067	172
AC161607.1	ENSMUSG00000116903	73	23	44	12	85	11	52	12
Acaca	ENSMUSG00000020532	559	140	447	82	557	121	494	41
Antxr2	ENSMUSG00000029338	3138	199	2527	322	3402	377	2661	386
Arl8b	ENSMUSG00000030105	1872	115	1674	157	2035	143	1684	79
Art4	ENSMUSG00000030217	412	21	326	68	438	37	298	89
Atl2	ENSMUSG00000059811	1046	62	921	131	1052	45	882	97
Atxn2	ENSMUSG00000042605	1186	207	1021	195	1395	65	1084	156
Bicra	ENSMUSG00000070808	259	26	236	41	304	46	218	25
Carnmt1	ENSMUSG00000024726	1249	241	1074	205	1403	244	1098	142
Carns1	ENSMUSG00000075289	373	32	286	27	383	53	289	49
Cited2	ENSMUSG00000039910	544	99	394	73	713	201	447	51
Cog5	ENSMUSG00000035933	1358	209	1191	133	1404	218	1168	103
Dcaf12l1	ENSMUSG00000045284	80	13	49	12	91	12	54	16
Dgke	ENSMUSG00000000276	588	81	456	38	628	87	483	103
Dhtkd1	ENSMUSG00000025815	67	13	46	8	98	24	53	13
Eml5	ENSMUSG00000051166	129	41	106	30	156	42	80	35
Fam84a	ENSMUSG00000020607	17	9	9	4	31	21	9	5
Fam84b	ENSMUSG00000072568	367	45	332	38	416	37	306	21
Fbxo3	ENSMUSG00000027180	1943	149	1714	52	1982	135	1735	155
Fgd4	ENSMUSG00000022788	952	82	788	50	1006	124	808	116
Fnip1	ENSMUSG00000035992	1689	280	1464	146	1814	202	1491	143
Gab1	ENSMUSG00000031714	1294	55	1103	118	1362	98	1139	128
Gm47283	ENSMUSG00000096768	121	68	133	40	164	81	81	49
Hbp1	ENSMUSG00000002996	1726	184	1572	147	1798	134	1552	102
Hccs	ENSMUSG00000031352	997	84	858	98	1041	148	890	60
Hspa12a	ENSMUSG00000025092	841	91	689	87	938	90	730	91
Jmy	ENSMUSG00000021690	1390	158	1200	90	1500	137	1234	114
Kcnh2	ENSMUSG00000038319	847	124	668	110	912	148	635	80
Kcnj2	ENSMUSG00000041695	1344	337	915	199	1453	205	945	234
Laptm4b	ENSMUSG00000022257	953	46	801	57	990	41	803	90
Lysmd4	ENSMUSG00000043831	351	36	275	32	399	40	293	44
Mef2a	ENSMUSG00000030557	4209	341	3602	223	4401	428	3617	183
Mef2d	ENSMUSG00000001419	2886	195	2459	352	3285	449	2564	203
Mif4gd	ENSMUSG00000020743	399	41	345	38	434	39	328	28
mt-Nd2	ENSMUSG00000064345	563587	132806	437281	99302	600917	101879	466929	126476
mt-Nd5	ENSMUSG00000064367	810634	162252	639879	106231	854483	106054	682450	132819
Myo5c	ENSMUSG00000033590	102	18	90	31	150	35	66	21
Nnt	ENSMUSG00000025453	5147	1691	3697	588	6332	1498	4488	661
Osbpl1a	ENSMUSG00000044252	1430	127	1177	138	1477	68	1236	73
Pkd2l2	ENSMUSG00000014503	119	20	90	8	123	12	80	8
Plag1	ENSMUSG00000003282	111	11	86	16	110	13	71	14
Plin5	ENSMUSG00000011305	1427	167	1055	258	1589	193	1116	132
Plxnb3	ENSMUSG00000031385	47	14	32	7	53	9	26	4
Pnrc1	ENSMUSG00000040128	1394	186	1223	251	1670	151	1176	94
Prox1	ENSMUSG00000010175	2013	472	1483	298	2390	394	1685	308
Ptpru	ENSMUSG00000028909	76	23	56	21	65	20	34	14
Pygo1	ENSMUSG00000034910	734	66	583	66	821	99	642	73
Retreg1	ENSMUSG00000022270	4914	645	4621	579	5580	346	4633	468
Rorc	ENSMUSG00000028150	820	185	636	99	951	111	730	106
Rsbn1l	ENSMUSG00000039968	486	32	495	68	547	61	451	62
Sall4	ENSMUSG00000027547	38	19	25	17	54	31	20	2
Sc5d	ENSMUSG00000032018	453	51	403	59	480	34	405	25
Slc25a22	ENSMUSG00000019082	545	113	458	107	575	57	418	70
Slc25a46	ENSMUSG00000024259	1632	281	1428	165	1805	227	1528	184
Slc40a1	ENSMUSG00000025993	540	85	413	68	525	93	369	33
Slc5a6	ENSMUSG00000006641	210	13	153	42	237	30	148	21
Slc9a2	ENSMUSG00000026062	118	24	98	34	103	34	64	9

Slf1	ENSMUSG00000021597	700	128	591	68	734	132	561	98
Smarcd1	ENSMUSG00000023018	561	16	444	69	610	82	467	65
Sobp	ENSMUSG00000038248	528	23	434	59	594	52	453	47
Sorcs2	ENSMUSG00000029093	323	58	246	26	327	75	216	35
Sp4	ENSMUSG00000025323	262	26	223	14	286	22	209	16
Stk39	ENSMUSG00000027030	2038	169	1731	250	2027	268	1564	136
Tmem161a	ENSMUSG00000002342	695	40	602	58	708	61	579	56
Tmem170b	ENSMUSG00000087370	544	52	444	44	603	78	462	26
Tmem238	ENSMUSG00000030431	17	16	16	5	31	9	10	4
Tob1	ENSMUSG00000037573	692	101	631	35	830	74	668	42
Trmt2b	ENSMUSG00000067369	1295	120	1099	134	1300	53	1109	91
Ttc30a1	ENSMUSG00000075271	133	38	110	17	165	28	117	20
Ttc30b	ENSMUSG00000075273	431	88	337	29	476	71	363	72
Txnip	ENSMUSG00000038393	13919	3376	11722	2478	18708	3486	12475	1810
Ubc	ENSMUSG00000008348	13447	4055	10872	1508	16818	2949	12228	2263
Ube2b	ENSMUSG00000020390	4614	513	4036	428	4668	348	4142	342
Ulk1	ENSMUSG00000029512	1687	77	1424	156	1856	253	1452	92
Vps13a	ENSMUSG00000046230	1184	126	1087	104	1354	142	1101	159
Ythdf3	ENSMUSG00000047213	1491	106	1342	61	1508	114	1305	117
Zbtb18	ENSMUSG00000063659	834	109	672	89	963	89	675	105
Zfp292	ENSMUSG00000039967	872	86	749	89	898	77	715	84

Supplementary Table S9. RNASeq data: gene clusters.

Gene Symbol	Ensembl gene id	WT Vehicle		WT AngII		PKN2Het Vehicle		PKN2Het AngII	
		**Mean**	**SD**	**Mean**	**SD**	**Mean**	**SD**	**Mean**	**SD**
**Complement**									
C1qa	ENSMUSG00000036887	895	96	1523	250	883	97	1362	183
C1qb	ENSMUSG00000036905	826	111	1513	429	782	128	1330	245
C1qc	ENSMUSG00000036896	886	106	1454	199	845	131	1290	171
C1qtnf3	ENSMUSG00000058914	3	2	341	632	3	2	118	157
C1qtnf5	ENSMUSG00000079592	53	6	100	38	61	17	87	20
C1qtnf6	ENSMUSG00000022440	122	21	426	250	120	11	348	107
C1qtnf7	ENSMUSG00000061535	178	26	294	78	174	31	227	32
C3ar1	ENSMUSG00000040552	234	31	502	241	242	41	380	97
C4b	ENSMUSG00000073418	231	28	604	141	295	41	861	703
C5ar1	ENSMUSG00000049130	107	17	173	40	78	18	156	56
Cfb	ENSMUSG00000090231	36	7	140	61	33	15	114	78
**Extracellular matrix**									
Acan	ENSMUSG00000030607	4	3	66	49	3	1	32	34
Aspn	ENSMUSG00000021388	750	159	3970	5012	798	171	1825	1043
Bgn	ENSMUSG00000031375	5175	520	12514	5386	5152	464	9491	2127
Ccdc80	ENSMUSG00000022665	1816	295	3479	1261	1843	168	2847	551
Col11a1	ENSMUSG00000027966	2	3	52	76	2	2	12	11
Col12a1	ENSMUSG00000032332	47	12	495	623	42	10	315	288
Col14a1	ENSMUSG00000022371	466	75	1493	1179	377	75	1075	556
Col15a1	ENSMUSG00000028339	2833	383	5668	1135	3175	509	5335	1392
Col16a1	ENSMUSG00000040690	187	39	603	471	192	60	420	186
Col18a1	ENSMUSG00000001435	248	28	790	244	293	35	541	181
Col1a1	ENSMUSG00000001506	1941	334	10243	9888	2115	63	5954	3420
Col1a2	ENSMUSG00000029661	2657	250	11812	10867	2786	231	7551	4300
Col3a1	ENSMUSG00000026043	5494	744	25906	20347	5871	824	16747	9657
Col4a1	ENSMUSG00000031502	12856	2211	23909	1751	13123	855	20837	3837
Col4a2	ENSMUSG00000031503	9490	1105	15270	591	9814	526	14603	2361
Col4a3	ENSMUSG00000079465	203	49	299	93	199	27	307	35
Col4a4	ENSMUSG00000067158	345	73	497	114	364	54	494	37
Col4a5	ENSMUSG00000031274	763	91	1159	304	773	91	971	147
Col5a1	ENSMUSG00000026837	1205	173	3410	1633	1238	85	2682	1054
Col5a2	ENSMUSG00000026042	878	65	4055	3237	927	105	2738	1563
Col5a3	ENSMUSG00000004098	576	178	985	63	666	103	847	222
Col6a1	ENSMUSG00000001119	2071	252	3943	1376	2047	155	3242	704
Col6a2	ENSMUSG00000020241	2038	291	3824	981	2062	139	3256	743
Col6a3	ENSMUSG00000048126	1170	165	2685	1137	1119	203	1895	452
Col7a1	ENSMUSG00000025650	4	6	26	21	5	4	11	6
Col8a1	ENSMUSG00000068196	704	96	3461	2170	747	153	2473	1081
Col8a2	ENSMUSG00000056174	9	5	180	298	14	4	86	85
Col9a2	ENSMUSG00000028626	6	1	31	38	7	4	21	11
Comp	ENSMUSG00000031849	39	8	325	426	69	9	179	129
Crtap	ENSMUSG00000032431	384	57	511	72	379	32	471	67
Cthrc1	ENSMUSG00000054196	3	3	221	380	5	2	92	112
Ecm1	ENSMUSG00000028108	452	86	696	127	464	82	720	136
Egflam	ENSMUSG00000042961	374	53	240	63	279	55	262	40
Eln	ENSMUSG00000029675	512	176	2249	1534	591	55	1327	606
Fbln2	ENSMUSG00000064080	1827	201	3200	493	1966	214	3008	422
Fbn1	ENSMUSG00000027204	2671	543	7690	2514	3011	303	6532	2277
Fbn2	ENSMUSG00000024598	25	8	112	120	38	10	67	32
Fgl2	ENSMUSG00000039899	863	65	1693	575	912	106	1331	391
Flna	ENSMUSG00000031328	4014	455	5769	673	3783	774	4951	309
Fn1	ENSMUSG00000026193	1579	300	7830	6886	1494	199	4711	3367
Frem1	ENSMUSG00000059049	8	10	51	68	8	4	31	24
Has2	ENSMUSG00000022367	27	6	50	13	28	8	42	6
Hspg2	ENSMUSG00000028763	12506	1017	15279	1628	12610	707	14990	1190
Lama2	ENSMUSG00000019899	3507	352	3968	325	3362	180	4015	379
Lama4	ENSMUSG00000019846	2464	138	3226	192	2291	264	3050	270
Lamb1	ENSMUSG00000002900	2656	308	3500	191	2690	265	3282	467
Lamc1	ENSMUSG00000026478	4969	490	7157	380	5155	283	6688	486

Lox	ENSMUSG00000024529	89	9	1050	1321	94	21	476	436
Loxl1	ENSMUSG00000032334	618	97	1447	604	687	57	1181	257
Loxl2	ENSMUSG00000034205	724	133	1635	127	683	84	1403	383
Loxl3	ENSMUSG00000000693	104	36	311	233	87	12	196	70
Lum	ENSMUSG00000036446	1447	238	3824	2686	1553	192	2795	564
Matn2	ENSMUSG00000022324	417	57	667	192	429	35	559	39
Mfap2	ENSMUSG00000060572	20	2	69	61	23	4	41	14
Mfap3	ENSMUSG00000020522	454	25	541	33	431	20	503	22
Mfap3l	ENSMUSG00000031647	512	38	351	27	511	51	378	15
Mfap4	ENSMUSG00000042436	202	18	1249	1153	245	36	712	324
Mfap5	ENSMUSG00000030116	411	43	1634	1033	404	45	1132	430
Mgp	ENSMUSG00000030218	1671	257	3299	1832	1627	149	2424	267
Mxra7	ENSMUSG00000020814	353	43	549	114	334	33	485	81
Mxra8	ENSMUSG00000029070	431	61	782	329	511	145	714	119
Nid1	ENSMUSG00000005397	3926	530	6347	1387	3634	332	5323	725
Nid2	ENSMUSG00000021806	561	130	914	118	537	62	895	114
Ntn1	ENSMUSG00000020902	1895	146	1435	153	2064	197	1793	251
P3h1	ENSMUSG00000028641	217	25	313	43	242	18	308	55
P3h3	ENSMUSG00000023191	185	14	319	100	179	28	278	54
P4ha3	ENSMUSG00000051048	2	2	23	32	1	1	11	10
Pcolce	ENSMUSG00000029718	778	81	1236	178	675	91	1133	173
Pcolce2	ENSMUSG00000015354	412	82	494	81	352	55	503	72
Plod3	ENSMUSG00000004846	475	118	658	87	472	51	605	62
Postn	ENSMUSG00000027750	1288	354	16369	19036	1325	317	10262	8108
Prg4	ENSMUSG00000006014	323	89	484	130	321	33	596	281
Sparc	ENSMUSG00000018593	7512	635	18831	6579	7355	933	14409	2942
Vcan	ENSMUSG00000021614	730	115	1503	151	665	105	1354	355
**Interferon signalling**									
Ifi203	ENSMUSG00000039997	891	161	1230	255	880	117	940	84
Ifi204	ENSMUSG00000073489	206	34	433	95	196	40	334	43
Ifi209	ENSMUSG00000043263	52	15	126	69	50	17	92	45
Ifi211	ENSMUSG00000026536	185	36	351	104	178	18	256	21
Ifi27l2a	ENSMUSG00000079017	237	35	439	84	218	39	420	49
Ifi30	ENSMUSG00000031838	101	27	193	76	87	20	127	31
Ifih1	ENSMUSG00000026896	299	32	446	46	329	56	378	53
Ifit1	ENSMUSG00000034459	187	43	329	102	161	24	234	21
Ifit2	ENSMUSG00000045932	547	92	934	140	539	58	669	52
Ifit3	ENSMUSG00000074896	336	93	626	210	311	45	430	29
Ifit3b	ENSMUSG00000062488	122	30	208	57	123	22	150	9
Ifitm2	ENSMUSG00000060591	875	131	1176	80	841	117	1097	135
Ifitm3	ENSMUSG00000025492	1226	258	1689	136	1126	147	1515	182
Ifngr1	ENSMUSG00000020009	1109	126	1400	78	1101	128	1324	109
Irf5	ENSMUSG00000029771	73	13	148	22	78	12	119	35
Irf7	ENSMUSG00000025498	220	65	413	114	189	26	332	52
Irf8	ENSMUSG00000041515	118	13	229	76	128	21	193	26
Isg20	ENSMUSG00000039236	86	30	130	16	102	27	144	32
**Mitochondria**									
Acaa2	ENSMUSG00000036880	13252	1380	8259	2391	12324	1059	9258	1215
Aco2	ENSMUSG00000022477	41047	2512	30674	4778	41075	2487	35785	2269
Afg1l	ENSMUSG00000038302	1113	135	844	176	1027	95	958	51
Aldh2	ENSMUSG00000029455	3628	278	2621	303	3753	239	3023	178
Atp5a1	ENSMUSG00000025428	73890	5007	52519	8597	73310	4969	61155	3605
Atp5b	ENSMUSG00000025393	81983	4027	60525	7907	80175	3926	68956	5579
Atp5d	ENSMUSG00000003072	5810	313	4470	769	5730	539	5074	535
Atp5e	ENSMUSG00000016252	4682	460	3624	408	4707	229	4130	508
Atp5g3	ENSMUSG00000018770	18004	1331	13448	2291	18271	996	15327	1271
Atp5o	ENSMUSG00000022956	14204	841	11103	1882	13615	536	12332	665
Bcat2	ENSMUSG00000030826	1208	96	917	126	1160	104	1012	77
Cars2	ENSMUSG00000056228	540	40	408	58	563	48	458	53
Clpp	ENSMUSG00000002660	608	48	457	72	598	7	499	95
Clpx	ENSMUSG00000015357	2210	180	1776	257	2247	186	1932	167
Cluh	ENSMUSG00000020741	5745	385	4121	818	6093	799	5032	263
Coq10a	ENSMUSG00000039914	3593	171	2600	450	3601	150	3083	271
Coq2	ENSMUSG00000029319	1358	71	1066	109	1353	65	1154	62
Coq7	ENSMUSG00000030652	1122	100	786	155	1106	99	884	88

Coq8a	ENSMUSG00000026489	8867	811	6241	1021	9606	1491	7168	332
Coq9	ENSMUSG00000031782	6190	445	4627	906	6154	410	5544	503
Cox4i1	ENSMUSG00000031818	22284	812	17754	1611	22070	1141	19696	1055
Cox5a	ENSMUSG00000000088	11595	608	8951	1587	11127	414	10060	740
Cox5b	ENSMUSG00000061518	10424	672	8385	1343	10221	301	9039	577
Cox7a1	ENSMUSG00000074218	8954	697	6119	1540	8610	451	7040	827
Cox8b	ENSMUSG00000025488	6504	621	4961	1023	6394	477	5556	903
Cs	ENSMUSG00000005683	24899	2214	17482	3393	24790	1691	20831	1885
Decr1	ENSMUSG00000028223	8479	863	5924	1566	7835	344	6512	520
Diablo	ENSMUSG00000029433	1178	83	980	91	1146	35	1105	48
Dlst	ENSMUSG00000004789	11654	750	8617	1895	11270	555	10153	788
Echs1	ENSMUSG00000025465	4828	268	3779	465	4896	284	4167	357
Etfa	ENSMUSG00000032314	11167	800	7804	1484	10775	533	8929	812
Etfb	ENSMUSG00000004610	8827	431	6069	1637	8450	419	6927	725
Etfdh	ENSMUSG00000027809	13588	811	9757	2314	12812	772	10703	771
Fh1	ENSMUSG00000026526	7500	576	5659	1050	7018	180	6388	586
Fmc1	ENSMUSG00000019689	622	55	458	87	580	55	484	71
Gfm1	ENSMUSG00000027774	4053	240	3035	442	3973	430	3372	259
Gfm2	ENSMUSG00000021666	1266	144	1001	118	1229	127	1128	100
Got2	ENSMUSG00000031672	15403	947	11510	1822	15607	1320	13207	1045
Gpd2	ENSMUSG00000026827	401	24	334	32	392	48	398	25
Hccs	ENSMUSG00000031352	997	84	858	98	1041	148	890	60
Iars2	ENSMUSG00000026618	2409	99	1873	136	2398	100	2171	120
Idh2	ENSMUSG00000030541	25832	1566	18548	3665	26906	2049	21457	2197
Idh3a	ENSMUSG00000032279	10211	1028	7707	867	10029	629	8753	799
Idh3b	ENSMUSG00000027406	11455	1144	8621	1973	11227	459	9312	738
Idh3g	ENSMUSG00000002010	6376	459	4785	580	6418	422	4954	479
Immt	ENSMUSG00000052337	11137	723	8672	1433	10924	555	9873	547
Lonp1	ENSMUSG00000041168	2317	80	2025	163	2376	150	2252	87
Malsu1	ENSMUSG00000029815	281	28	208	15	260	23	236	30
Mars2	ENSMUSG00000046994	370	47	284	34	364	34	304	25
Mcub	ENSMUSG00000027994	41	14	77	15	37	4	50	10
Mdh2	ENSMUSG00000019179	16876	703	12970	2129	16369	1034	14499	734
Me3	ENSMUSG00000030621	1244	36	938	86	1271	81	1046	52
Mfn1	ENSMUSG00000027668	8217	628	6382	1129	8242	170	7059	753
Mfn2	ENSMUSG00000029020	15414	955	11184	2042	15810	1644	13241	705
Mgme1	ENSMUSG00000027424	350	28	255	46	337	12	267	30
Miga1	ENSMUSG00000054942	412	61	323	8	409	40	383	35
Mipep	ENSMUSG00000021993	1364	63	983	167	1394	126	1103	71
Mpv17	ENSMUSG00000107283	1098	32	873	90	1118	80	934	104
Mrpl14	ENSMUSG00000023939	682	54	516	77	739	35	586	87
Mrpl16	ENSMUSG00000024683	961	36	784	75	930	52	848	97
Mrpl28	ENSMUSG00000024181	1210	39	882	132	1171	81	1020	121
Mrpl37	ENSMUSG00000028622	1184	43	904	135	1161	34	1046	74
Mrpl38	ENSMUSG00000020775	704	31	534	72	674	41	611	58
Mrpl39	ENSMUSG00000022889	1365	91	1053	180	1335	40	1087	56
Mrpl4	ENSMUSG00000003299	1028	38	792	138	1029	93	915	137
Mrpl45	ENSMUSG00000018882	1187	97	837	153	1100	58	1043	54
Mrps26	ENSMUSG00000037740	355	39	258	24	351	18	326	26
Mrps35	ENSMUSG00000040112	1312	76	991	219	1236	59	1132	63
Mrps6	ENSMUSG00000039680	361	20	285	16	354	19	300	4
mt-Nd2	ENSMUSG00000064345	563587	132806	437281	99302	600917	101879	466929	126476
mt-Nd5	ENSMUSG00000064367	810634	162252	639879	106231	854483	106054	682450	132819
mt-Rnr2	ENSMUSG00000064339	356240	61989	247940	69255	362883	53161	276407	38517
Mtfp1	ENSMUSG00000004748	1636	82	1103	322	1626	114	1320	90
Mtfr1l	ENSMUSG00000046671	2563	65	2169	189	2622	122	2390	105
Mtg2	ENSMUSG00000039069	366	31	275	26	387	9	312	16
Mto1	ENSMUSG00000032342	481	27	368	46	450	40	405	41
Nadk2	ENSMUSG00000022253	1247	102	792	168	1254	222	893	160
Ndufa10	ENSMUSG00000026260	9566	706	7436	1132	9920	331	8450	886
Ndufa8	ENSMUSG00000026895	4878	294	3771	635	4757	148	4279	331
Ndufa9	ENSMUSG00000000399	9480	996	7084	1337	9287	369	7867	703
Ndufs1	ENSMUSG00000025968	15975	1815	11734	2163	15650	1579	13236	1027
Ndufs2	ENSMUSG00000013593	16031	729	11634	1773	15858	991	13476	1336
Ndufs3	ENSMUSG00000005510	6047	349	4620	872	5917	121	5197	447
Ndufs7	ENSMUSG00000020153	3525	187	2485	513	3595	317	2923	370
Ndufv1	ENSMUSG00000037916	9773	762	6974	1216	9669	615	8148	626

Ogdh	ENSMUSG00000020456	39640	2492	29734	4328	41635	5116	35518	902
Opa1	ENSMUSG00000038084	5644	514	4210	706	5735	550	5001	562
Oxa1l	ENSMUSG00000000959	1341	57	1036	150	1356	195	1142	95
Oxct1	ENSMUSG00000022186	26538	1570	20521	2412	25456	2358	23051	1272
Oxsm	ENSMUSG00000021786	592	47	470	26	633	71	520	20
Pdf	ENSMUSG00000078931	565	42	418	72	575	29	492	19
Pdha1	ENSMUSG00000031299	24973	2883	19882	1983	24844	2183	21298	1610
Pdk2	ENSMUSG00000038967	7045	426	4922	991	7351	661	5815	537
Pdk3	ENSMUSG00000035232	44	14	83	17	53	21	69	7
Pdp1	ENSMUSG00000049225	1052	157	827	97	1038	87	873	99
Pdp2	ENSMUSG00000048371	822	182	433	87	839	116	560	111
Pdpr	ENSMUSG00000033624	2409	176	1771	254	2523	330	2118	147
Pmpca	ENSMUSG00000026926	2809	172	2292	228	2800	157	2538	100
Sdha	ENSMUSG00000021577	26565	1713	17836	2991	26775	2129	20539	1624
Sdhb	ENSMUSG00000009863	12188	749	9065	1722	11819	503	9978	990
Sdhc	ENSMUSG00000058076	7127	66	5010	729	7101	434	5730	395
Sfxn3	ENSMUSG00000025212	216	19	310	29	224	5	272	31
Slc25a11	ENSMUSG00000014606	8077	561	5928	1242	7992	341	6986	662
Slc25a12	ENSMUSG00000027010	4184	351	2893	395	4194	379	3611	151
Slc25a13	ENSMUSG00000015112	2989	324	2199	145	3029	276	2574	268
Slc25a20	ENSMUSG00000032602	3086	349	2262	477	3063	140	2521	210
Slc25a22	ENSMUSG00000019082	545	113	458	107	575	57	418	70
Slc25a24	ENSMUSG00000040322	184	16	278	63	197	22	246	35
Slc25a3	ENSMUSG00000061904	25015	1211	18063	2479	25124	1400	20806	1485
Sod2	ENSMUSG00000006818	12266	831	8665	1885	11982	506	9979	664
Suclg1	ENSMUSG00000052738	6364	514	4599	1021	6012	279	5327	363
Suclg2	ENSMUSG00000061838	4105	321	2961	428	4037	119	3294	228
Tars2	ENSMUSG00000028107	667	59	489	66	606	23	549	66
Tcaim	ENSMUSG00000046603	1091	140	752	164	1078	129	865	134
Tufm	ENSMUSG00000073838	3132	215	2311	479	3147	191	2714	265
Twnk	ENSMUSG00000025209	604	21	452	67	607	59	505	23
Ucp3	ENSMUSG00000032942	1264	355	659	227	1281	331	698	271
Uqcc1	ENSMUSG00000005882	3454	270	2476	553	3363	150	2909	228
Uqcrc1	ENSMUSG00000025651	15130	710	10190	2080	15168	907	12233	1293
Uqcrfs1	ENSMUSG00000038462	13197	914	10056	1838	12772	692	11183	890
Vdac1	ENSMUSG00000020402	17517	801	13326	1578	17010	633	14751	342
Vdac3	ENSMUSG00000008892	6247	446	4930	675	5939	119	5490	330
**Structural**									
Acta1	ENSMUSG00000031972	6773	1395	24772	10833	8570	3863	28601	14894
Actb	ENSMUSG00000029580	7471	1804	10178	1677	7763	1384	9365	1200
Actg1	ENSMUSG00000062825	6824	1047	8916	1194	7413	1015	8405	1266
Actg2	ENSMUSG00000059430	11	6	37	14	12	6	20	12
Actn1	ENSMUSG00000015143	522	79	925	273	524	61	761	132
Actn4	ENSMUSG00000054808	2213	272	2742	211	2012	203	2417	171
Actr2	ENSMUSG00000020152	2099	200	2493	267	2101	71	2352	201
Actr3	ENSMUSG00000026341	2536	120	3001	264	2399	43	2908	199
Agrn	ENSMUSG00000041936	958	125	1295	154	947	79	1136	70
Aif1	ENSMUSG00000024397	63	12	133	73	51	11	95	13
Ank2	ENSMUSG00000032826	3236	377	2426	262	3245	406	2934	430
Ankrd1	ENSMUSG00000024803	20150	6483	57246	17002	19310	6244	49450	10874
Ankrd23	ENSMUSG00000067653	8223	1670	13521	3447	9104	579	14731	4085
Anks1	ENSMUSG00000024219	1418	123	1011	201	1347	177	1118	141
Anln	ENSMUSG00000036777	56	22	225	55	56	7	174	50
Arpc1b	ENSMUSG00000029622	814	81	1186	179	737	113	1071	150
Arpc2	ENSMUSG00000006304	2337	43	2757	185	2338	47	2652	121
Arpc3	ENSMUSG00000029465	1134	39	1490	168	1115	115	1392	88
Arpc5	ENSMUSG00000008475	864	45	1233	156	871	66	1121	40
Arpin	ENSMUSG00000039043	239	19	299	19	258	25	280	26
Cald1	ENSMUSG00000029761	1649	323	2616	191	1624	256	2205	422
Capg	ENSMUSG00000056737	183	29	364	36	182	36	328	70
Capza1	ENSMUSG00000070372	1251	78	1608	138	1166	148	1428	124
Cfl1	ENSMUSG00000056201	2052	87	2670	304	1872	236	2490	204
Cilp	ENSMUSG00000042254	294	76	3092	3273	401	134	2249	1515
Ckap2	ENSMUSG00000037725	33	16	131	46	25	5	95	45
Ckap2l	ENSMUSG00000048327	30	10	123	39	25	8	110	29
Ckap4	ENSMUSG00000046841	756	159	1094	75	815	103	1104	165

Cnn1	ENSMUSG00000001349	37	23	130	75	42	17	53	24
Cnn2	ENSMUSG00000004665	793	93	1039	52	856	80	1002	98
Cnn3	ENSMUSG00000053931	1009	125	1438	123	955	121	1319	119
Coro1a	ENSMUSG00000030707	175	29	270	73	171	24	228	26
Coro1b	ENSMUSG00000024835	688	47	858	53	695	47	802	76
Cotl1	ENSMUSG00000031827	234	40	369	66	208	50	335	22
Csrp1	ENSMUSG00000026421	868	80	1281	109	889	121	1075	145
Csrp2	ENSMUSG00000020186	180	37	542	354	165	20	315	127
Cttn	ENSMUSG00000031078	793	94	1056	113	811	56	962	95
Dbn1	ENSMUSG00000034675	170	35	385	71	169	25	330	74
Dbnl	ENSMUSG00000020476	472	46	575	30	501	31	573	37
Emilin1	ENSMUSG00000029163	473	106	835	204	519	28	812	91
Eml2	ENSMUSG00000040811	379	40	285	35	408	44	337	30
Eml5	ENSMUSG00000051166	129	41	106	30	156	42	80	35
Enah	ENSMUSG00000022995	2820	352	4077	860	3138	213	4243	467
Fscn1	ENSMUSG00000029581	817	47	1186	92	831	115	1140	241
Ift122	ENSMUSG00000030323	278	32	496	94	309	39	488	78
Ift81	ENSMUSG00000029469	1002	129	666	78	994	49	708	78
Jpt1	ENSMUSG00000020737	841	76	987	49	790	37	1002	107
Jpt2	ENSMUSG00000024165	169	19	197	35	141	33	226	44
Map10	ENSMUSG00000050930	149	21	98	15	151	17	112	10
Map1b	ENSMUSG00000052727	582	102	873	51	444	82	777	113
Map1lc3a	ENSMUSG00000027602	4603	222	3864	305	4713	378	4049	447
Map6	ENSMUSG00000055407	108	31	142	36	87	9	129	34
Mapre1	ENSMUSG00000027479	1639	151	1876	92	1651	91	1859	64
Msn	ENSMUSG00000031207	4381	327	6174	266	4286	400	5734	498
Mybpc2	ENSMUSG00000038670	292	36	901	354	321	110	773	309
Myh10	ENSMUSG00000020900	964	132	1462	186	988	96	1366	213
Myh14	ENSMUSG00000030739	1825	190	1436	214	1992	381	1613	123
Myh6	ENSMUSG00000040752	416670	31586	295124	67491	432469	60382	365274	31724
Myh7	ENSMUSG00000053093	1672	510	20844	27743	2490	1006	11438	8811
Myh9	ENSMUSG00000022443	4467	917	6229	813	4465	679	5733	609
Myl1	ENSMUSG00000061816	777	84	1354	639	674	113	1186	282
Myl6	ENSMUSG00000090841	3039	174	4606	743	2959	365	4098	492
Myl9	ENSMUSG00000067818	507	113	744	105	504	152	536	41
Mylip	ENSMUSG00000038175	408	59	322	55	361	21	336	42
Myo1c	ENSMUSG00000017774	2939	140	3445	280	2807	136	3414	203
Myo1d	ENSMUSG00000035441	367	44	489	47	360	56	474	39
Myo1e	ENSMUSG00000032220	382	52	541	82	377	40	487	59
Myo1f	ENSMUSG00000024300	97	11	170	34	88	10	163	43
Myo1g	ENSMUSG00000020437	50	9	89	21	55	12	90	33
Myo5a	ENSMUSG00000034593	359	55	615	165	371	102	518	152
Myo5c	ENSMUSG00000033590	102	18	90	31	150	35	66	21
Myof	ENSMUSG00000048612	346	89	640	167	312	56	541	78
Nes	ENSMUSG00000004891	1779	216	2677	688	1621	280	2378	347
Pfn1	ENSMUSG00000018293	2349	50	2712	75	2362	104	2642	191
Sgcb	ENSMUSG00000029156	2725	146	2351	41	2817	190	2512	150
Sgce	ENSMUSG00000004631	294	28	387	53	287	38	342	18
Smarcd1	ENSMUSG00000023018	561	16	444	69	610	82	467	65
Sntb2	ENSMUSG00000041308	632	76	913	145	638	68	853	99
Sptb	ENSMUSG00000021061	3814	253	2892	391	4015	448	3358	226
Tagln	ENSMUSG00000032085	483	160	931	216	458	122	542	128
Tagln2	ENSMUSG00000026547	1222	205	1863	181	1131	206	1710	250
Tcap	ENSMUSG00000007877	15023	5676	9524	3481	11808	3513	10365	2562
Tln1	ENSMUSG00000028465	3433	355	4129	304	3528	301	4361	274
Tmod1	ENSMUSG00000028328	6866	511	5456	542	6671	424	6180	413
Tmod4	ENSMUSG00000005628	378	37	254	60	361	43	317	59
Tnni3	ENSMUSG00000035458	63108	4479	42722	10485	61514	1321	48873	5226
Tnnt2	ENSMUSG00000026414	110161	8926	87055	9068	105408	3834	92241	8335
Tnnt3	ENSMUSG00000061723	6	2	29	39	5	3	17	17
Tpm2	ENSMUSG00000028464	338	60	565	58	358	58	477	79
Tpm3	ENSMUSG00000027940	1515	192	2133	211	1390	166	1854	103
Tpm4	ENSMUSG00000031799	3181	181	4770	635	3020	369	4360	322
Ttll1	ENSMUSG00000022442	921	104	531	110	965	54	680	86
Tuba1a	ENSMUSG00000072235	1839	223	2349	176	1762	189	2229	379
Tubb2a	ENSMUSG00000058672	572	55	703	94	505	25	707	148
Tubb2b	ENSMUSG00000045136	49	15	127	28	62	34	105	43

Tubb5	ENSMUSG00000001525	1852	114	2383	316	1680	188	2353	298
Tubb6	ENSMUSG00000001473	337	29	445	93	318	39	459	50
Vim	ENSMUSG00000026728	3763	407	7380	1381	3521	520	6206	983
Was	ENSMUSG00000031165	34	9	71	17	40	6	48	12
Whrn	ENSMUSG00000039137	481	110	307	100	542	79	382	97
Wipf1	ENSMUSG00000075284	572	54	783	47	562	70	635	43
Wisp1	ENSMUSG00000005124	27	8	174	241	30	12	82	66
Xirp2	ENSMUSG00000027022	23026	1933	46990	12463	24085	3291	47862	16560
Zyx	ENSMUSG00000029860	896	129	1214	76	876	143	1120	93

Supplementary Table S10. Body weights of adult mice. P values are for body weight after treatment with angiotensin II (AngII) for 7 d relative to baseline body weight (2-way ANOVA with Holm-Sidak's post-test) or for PKN2Het mice relative to wild-type (WT) mice at 42 weeks (t test). There were no significant differences between WT and PKN2Het mice at 12 weeks.

**12 weeks**	**Baseline body weight (g)**			**Body weight after 7 d AngII (g)**			**P value**
	**Mean**	**SEM**	**n**	**Mean**	**SEM**	**n**	
WT/Vehicle	28.18	0.75	5	28.24	0.72	5	P=0.071
PKN2Het/Vehicle	28.25	0.69	8	27.80	0.80	7	**P<0.001**
WT/AngII	28.96	0.72	5	28.74	0.56	5	P=0.6235
PKN2/AngII	29.42	0.76	8	28.47	0.72	7	P=0.1847
**42 weeks**	**WT**			**PKN2Het**			
	Mean	SEM	n	Mean	SEM	n	
	35.10	1.40	8	37.24	0.67	11	p=0.151

Supplementary Table S11. qPCR primers.

**Gene**	**Sense Primer (5'→3')**	**Antisense Primer (5'→3')**
Gapdh	TCACCACCATGGAGAAGGC	GCTAAGCAGTTGGTGGTGCA
Myh7	CATGCCAACCGTATGGCTG	GTTCCACGATGGCGATGTTC
Nppa	GATGGATTTCAAGAACCTGCTAGA	CTTCCTCAGTCTGCTCACTCA
Nppb	TCCAGCAGAGACCTCAAAATTC	CAGTGCGTTACAGCCCAAA
Tagln	GACTGCACTTCTCGGCTCAT	CCGAAGCTACTCTCCTTCCA

Supplementary references

60 Banyasz, T., Lozinskiy, I., Payne, C. E., Edelmann, S., Norton, B., Chen, B., Chen-Izu, Y., Izu, L. T. and Balke, C. W. (2008) Transformation of adult rat cardiac myocytes in primary culture. Exp Physiol. **93**, 370-382

61 Cerbai, E., Pino, R., Sartiani, L. and Mugelli, A. (1999) Influence of postnatal-development on I(f) occurrence and properties in neonatal rat ventricular myocytes. Cardiovasc Res. **42**, 416-423
